# Kinetic Trapping of Rylene Diimide Covalent Organic Cages

**DOI:** 10.1021/acs.joc.4c02547

**Published:** 2025-03-18

**Authors:** Sergey Fisher, Hsin-Hua Huang, Luise Sokoliuk, Alessandro Prescimone, Olaf Fuhr, Tomáš Šolomek

**Affiliations:** † Van’t Hoff Institute for Molecular Sciences, University of Amsterdam, Science Park 904, XH Amsterdam NL-1098, the Netherlands; ‡ Department of Chemistry, University of Basel, St. Johanns-Ring 19, Basel CH-4056, Switzerland; § Institute of Nanotechnology and Karlsruhe Nano Micro Facility, Karlsruhe Institute of Technology, Kaiserstraße 12, Karlsruhe DE-76131, Germany

## Abstract

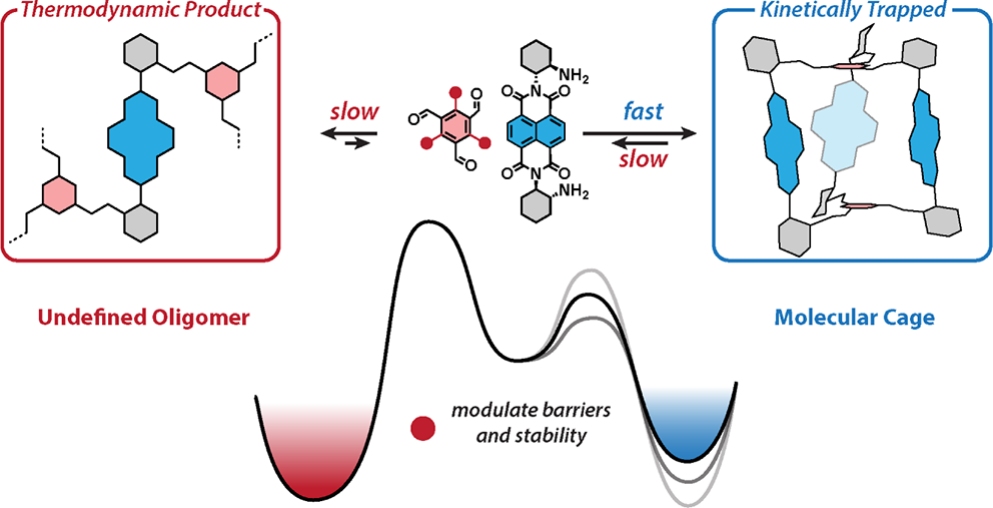

Formation of imine organic cages relies on the error correction of dynamic covalent chemistry. Here, we demonstrate kinetically trapped rylene diimide [2 + 3] cages formed in high yields, and we investigate the effect of substituents on their formation kinetics and stability. Thereby, we identified that alkoxy groups in 2,4,6-trialkoxy-1,3,5-triformylbenzene, which are used to stabilize covalent organic cages or COFs, act as stereoelectronic chameleons. They increase the electrophilicity of the tritopic aldehyde and the rate of the imine bond formation but simultaneously diminish its kinetic stability in solution. We also show that aldehydes present in the solution may have a detrimental effect on the cage’s kinetic stability. In addition, we observed [2 + 2] macrocycles as intermediates in the cage formation and decomposition. We propose that these intermediates represent interesting targets to explore the threshold at which an imine assembly with a rung structure may turn from thermodynamic to kinetic control. Generally, this work underscores critical factors governing the chemistry of kinetically trapped imine assemblies, such as steric bulk, (stereo)electronics, presence of catalysts, and water concentration.

## Introduction

Multicomponent molecular assembly guided by dynamic covalent chemistry (DCC) is a powerful tool to create intricate two- and three-dimensional structures, such as macrocycles,^1−3^ cages,^4,5^ molecular knots,^6^ or covalent organic frameworks^7^ in a single step. The extensive array of accomplished porous organic cages (POCs) promises that their structures can be tailored via DCC to achieve specific requirements. For example, POCs have found various applications in gas separation,^5,8−16^ molecular sieving,^17−21^ selective anion binding,^22−24^ chiral recognition,^25−27^ optoelectronics,^28−30^ or catalysis.^31−35^ Despite this versatility, accurately predicting the synthetic outcome in POC synthesis remains a considerable challenge in the field. The reversible nature of DCC implies that the products are formed under thermodynamic control,^36^ which in principle allows for predictions by comparing the heats of formation of POCs.^28,37−40^ Greenaway et al. demonstrated such computational workflows using density functional theory (DFT) that agreed well with the experiments and helped to rationalize the composition of complex product mixtures. Despite this success, there are cases that underscore the significance of the kinetics in the formation of POCs. Here, alternative reaction pathways may lead to unwanted byproducts, e.g., oligomers, or the POCs themselves might represent kinetically trapped products as observed previously by several groups using dynamic imine or alkyne metathesis chemistry.^4,41−47^ Furthermore, hard-to-predict circumstances, such as kinetic trapping through precipitation, can pose significant challenges in synthesis design. Computing kinetic pathways in POC formation is even more intricate than an accurate calculation of heat of formation. Yet, the kinetic factors could be leveraged to allow access to novel structures, such as asymmetric cages, and diverse dynamic covalent libraries that can be temporarily conserved out of equilibrium.^48,49^ Therefore, identification of POCs that are formed as kinetic traps in high yields is of considerable importance.^47^ They allow to study the factors that govern their formation and kinetic or thermodynamic stability informing future design. However, the lack of reversibility in the kinetic control fails to provide the error correction mechanism, which is typically detrimental to the reaction yield.

**Figure 1:**
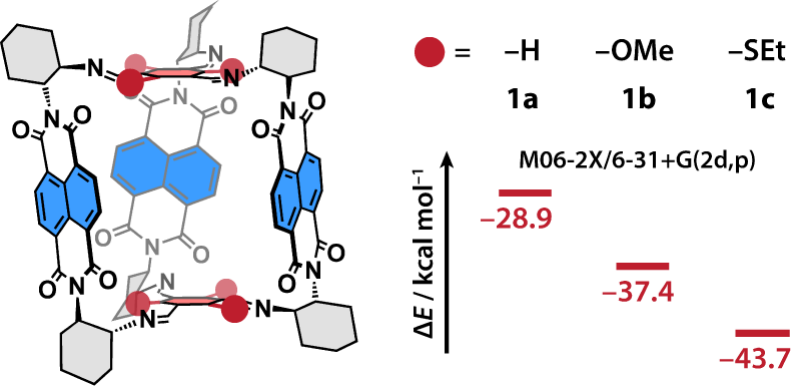
NDI cages with 1,3,5-iminoarene bridges with H (**1a**), Me–O (**1b**), and Et–S (**1c**) substituents. The heats of cage formation (red) are shown in kcal mol^–1^ (M06–2X/6–31+G(2d,p) level of theory).

Recently, we reported the high-yielding synthesis, textural, and optoelectronic properties of a family of [2 + 3] electron-poor rylene diimide POCs.^28,29,50^ Light excitation of naphthalene-1,4:5,8-bis(dicarbox-imide) (NDI) cage (**1a**, Figure 1) created a long-lived charge-separated state with an electron residing on one NDI unit and a hole on the bridge.^28,51^ Such long-lived states could be utilized in photocatalysis, which motivated us to synthesize analogous cages **1b** and **1c** with strong electron donors in the bridges to manipulate the excited state dynamics. In this work, we explored their synthesis and found that the rylene diimide cage formation is driven solely by kinetics. We show that electron-donating alkoxy substituents in the tritopic aldehydes used in the formation of POCs or COFs accelerate the imine formation but, at the same time, have a detrimental effect on the kinetic stability of the product. Our analysis of the cage formation underscores critical factors that govern the formation and stability of kinetically trapped imine POCs, such as steric bulk, (stereo)electronics, presence of catalysts, and water concentration.^52^

## Results and Discussion

**Scheme 1:**
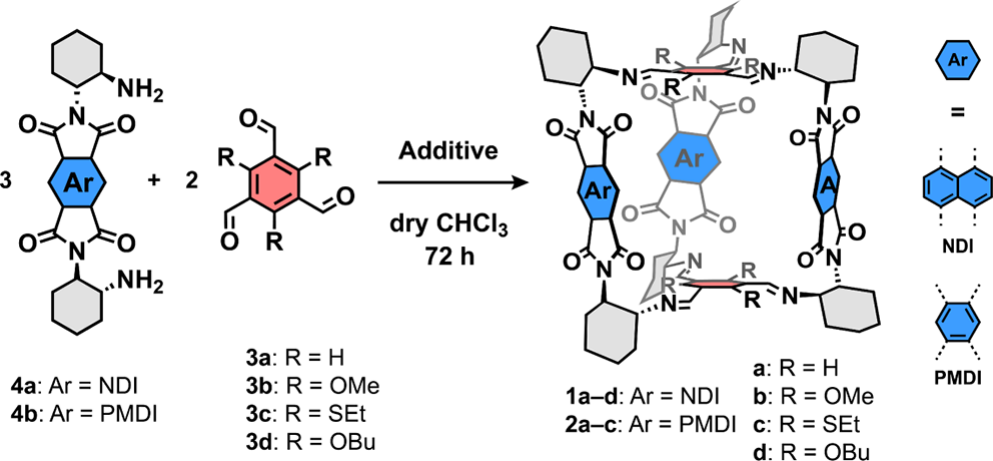
General Reaction of Diamines **4** with Aldehydes **3** Forming Cages **1a**–**d** and **2a**–**c**

The synthesis of **1a** was achieved by condensation of 1,3,5-triformylbenzene (**3a**, Scheme 1, Table 1) and the corresponding enantiomerically pure NDI diamine **4a** in good yield (60%; dry CHCl_3_: *c*_H2O_ = 7 ppm, 80 °C, 72 h). This and other structurally related cages^28,29^ could be successfully predicted in our previous work by the computational workflow used in recent literature^39,53,54^ (Figure 1). The same calculations also suggested that both **1b** and **1c** would be formed in high yields because their formation was markedly more exothermic than that of **1a**. We synthesized the required tritopic aldehydes **3** and reacted them with **4a** in dry CHCl_3_ at elevated temperature to establish the equilibrium (Scheme 1). Only a small amount of impure **1b** (Table 1, entry 1) and no **1c** could be detected even after a prolonged reaction time (14 days). To improve their synthesis, we added Sc(OTf)_3_ as a catalyst, or started the synthesis from the ditosylate salt of **4a** in anhydrous chloroform at 25 °C (Table 1, entries 2–3) to provide Brønsted acid catalysis. Similar to the initial attempts, no **1c** could be detected, and the yield of **1b** decreased. This suggests that the formation of the target cages might face a kinetic barrier to establish the equilibrium. Consequently, we attempted to overcome the reaction barrier by a combination of Sc(OTf)_3_ and elevated temperature (80 °C), but the reaction resulted in an unidentifiable product mixture with no sign of cage **1c**. We observed that the synthesis of **1b** from **4a** and **3b** at 25 °C in the absence of any Brønsted or Lewis acid catalyst markedly improved the purity of the crude product mixture and provided **1b** in 48% yield after purification (Table 1, entry 4). The outcome of this experiment was unexpected since cage **1a** can be formed at elevated temperatures in very good yields.^28^ We repeated the same set of experiments with PMDI diamine **4b** because we observed previously that **2a** formed in a process cleaner than that of **1a**. However, attempts to form **2b** paralleled the observations with **1b**, and the formation of **2b** exhibited 85% yield after purification by recycling gel permeation chromatography (rGPC) only at 25 °C without additives (Table 1, entry 5).^29^ Notably, a portion of the observed products were insoluble, likely oligomeric, byproducts that we could not characterize. All of our observations therefore imply that rylene diimide cages **1** and **2** probably represent kinetic rather than thermodynamic products in chloroform. Although the formation of **1c** and **2c** appears to proceed via a high barrier (Table 1, entries 6–7), all other cages could be isolated in good to high yields when synthesized at room temperature without the error-correction mechanism of DCC.

**Table 1:** Optimization of Reaction Conditions for the Synthesis of Cages **1a**–**d** and **2a**–**c**^a^

Entry	Cage^b^	Additive	*T*/°C	Yield^c^/%
1	**1b**	–	80	<20
2	**1b**	10 mol % Sc(OTf)_3_	25	8
3	**1b**	6 *p*-TsOH and exc. NEt_3_	25	<8
4	**1b**	–	25	48
5	**2b**	–	25	85
6	**1c**	–	25	–d
7	**2c**	–	25	–d
8	**1d**	–	25	45

a See the Supporting Information for the complete list of conditions.

b Synthesized from the corresponding trialdehyde **3** and diamine **4**.

c The yield of **1b** is based on isolated and dried product after purification by HPLC.

d No cage formation detected.

**Figure 2:**
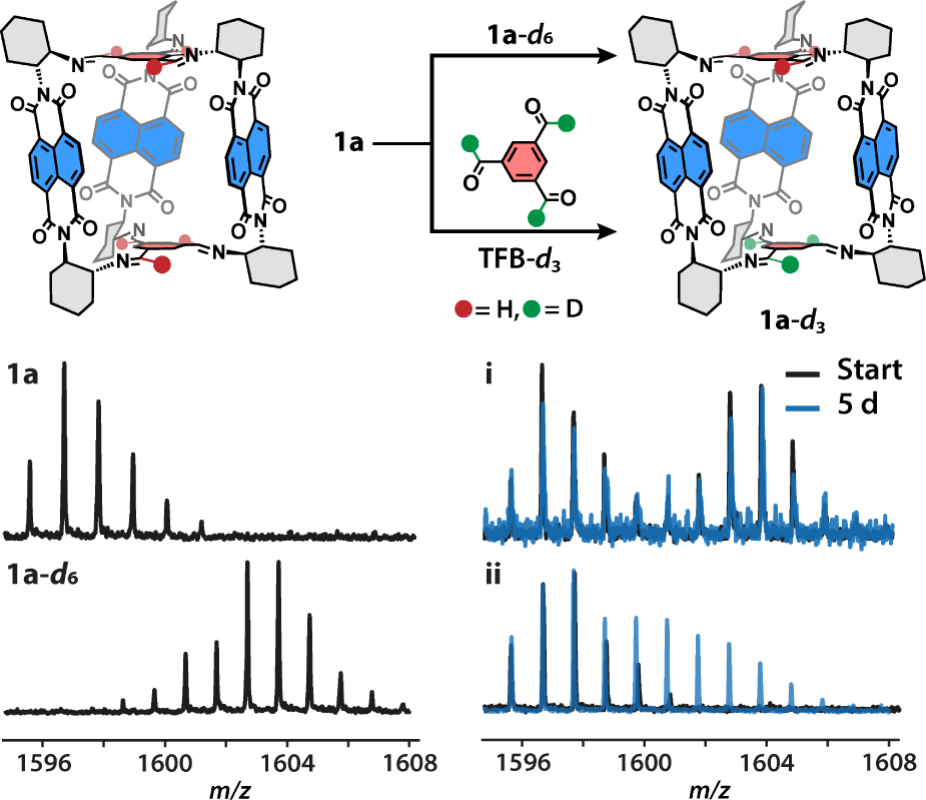
Scrambling of **1a** with isotopologue **1a**-*d*_6_ (top) or deuterated **3a** (bottom) to obtain mixed **1**-*d*_3_. MALDI-MS shows the isotopic pattern for **1a** and **1**-*d*_6_ and representative spectra upon (i) the scrambling of **1a** and **1**-*d*_6_ and (ii) the reaction of **1a** with **3a**-*d*_3_ in water-saturated CHCl_3_ with TFA at 60 °C.

To test the dynamic nature of the rylene cages, we synthesized the deuterated isotopologue **1a**-*d*_*6*_ with deuterium atoms in the imine positions in the bridge (Figure 2) and examined its scrambling with **1a**, similar to the previous work of Mastalerz et al.^41,42,44^ Both cages were mixed in CHCl_3_ for 96 h, and the reaction mixture was analyzed with matrix-assisted laser desorption ionization time-of-flight mass spectrometry (MALDI-MS). As can be seen in Table 2, regardless of the water content (7 and >700 ppm), temperature (25 and 60 °C), and the presence of an acid catalyst (TFA, 1 mol %), we did not observe the formation of **1a-***d*_3_ (Figures 2 and S50), the population of which should reach ∼50% in equilibrium. We noticed an increase in the signal-to-noise ratio over the course of the experiment, hinting at a slow decomposition of the cages under the selected conditions. The absence of the imine metathesis supports the hypothesis that cage **1a** is formed as a kinetic trap.

**Table 2:** Scrambling Conditions^a^ of **1a** with **1a**-*d*_6_ (Entries 1–6) or **3a**-*d*_3_ (Entries 7–8)

Entry	Reagents^b^	Solvent	*T*/°C	Result
1	**1a** + **1a**-*d*_6_	Anhyd. CHCl_3_	25	Decomp.
2	**1a** + **1a**-*d*_6_	Anhyd. CHCl_3_	60	Decomp.
3	**1a** + **1a**-*d*_6_ + TFA	Anhyd. CHCl_3_	25	Decomp.
4	**1a** + **1a**-*d*_6_	H_2_O sat. CHCl_3_	25	Decomp.
5	**1a** + **1a**-*d*_6_	H_2_O sat. CHCl_3_	60	Decomp.
6	**1a** + **1a**-*d*_6_ + TFA	H_2_O sat. CHCl_3_	25	Decomp.
7	**1a** + **3a**-*d*_6_	Anhyd. CHCl_3_	25	Decomp.
8	**1a** + **3a**-*d*_6_ + TFA	H_2_O sat. CHCl_3_	60	**1a**-*d*_3_^c^

a See the Supporting Information (Figure S50) for the corresponding MALDI-MS spectra.

b TFA was added as 1 mol % as an additive.

c Decomposition was observed. Note: decomp. = decomposition.

**Figure 3:**
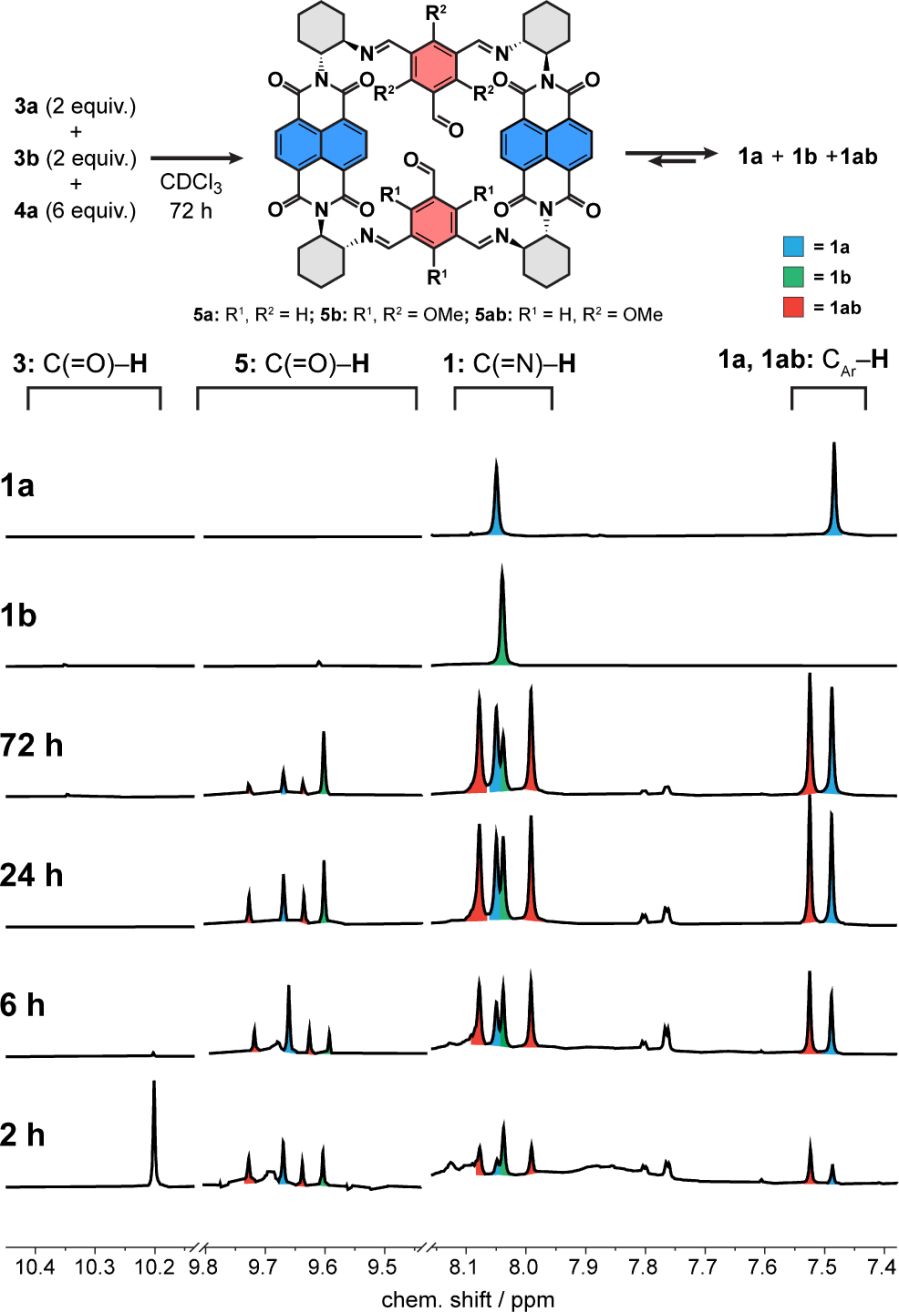
Kinetic self-sorting of **3a** and **3b** with **4a** in CDCl_3_. The intensities of the aldehydic resonances of macrocycles **5** (9.40–9.80 ppm) were enhanced 3-fold.

The substituents in the bridging units in **1** provide an opportunity to investigate the formation and stability of these kinetically trapped POCs. Previously, Bera and coworkers reported that the steric bulk and electronic properties of the MeO groups in **3b** endowed the analogous [6 + 4]-cage (CC3) with exceptional kinetic stability.^5,55,56^ However, cages **1b** and **2b** seem to be more sensitive to the environment than **1a** and **2a**. The electron-donating nature and the size of MeO and EtS groups (Hammett *σ*_p_ < 0) suggest that they increase the barrier of imine formation required to assemble the cage, and they also make the resulting cage thermodynamically more stable.^57^ The former assumes an amine nucleophilic attack as the rate-limiting step, as showed previously when imine formation and metathesis occur in a dry organic solvent without a catalyst.^58,59^ The latter is reflected in the computed heats of formation (Figure 1). The electronic and steric factors clearly prevent the formation of **1c** or **2c** as supported by our DFT calculations of the geometry of **3c** (see Figure S74 for further discussion). However, replacing MeO with bulkier BuO groups in the trialdehyde (**3d**) did not prevent formation of the corresponding cage **1d** at room temperature (Table 1, entry 8) despite a markedly stronger electron donor and larger steric bulk than the EtS group. X-ray diffraction analysis of single crystals of **1b** and **1d** revealed that all three alkoxy substituents are significantly rotated (>74°, Figures S47 and S48) out of the arene bridge plane. This is confirmed by DFT calculations that show a similar effect in trialdehyde **3b** (83.4°, Figure S49). Note that the rotation not only dimishes the electron-donating ability of MeO groups, it, in fact, turns them into electron acceptors. Such stereoelectronic effect has been observed and applied to control reactivity before.^60−62^ Briefly, alkoxy groups display spatial anisotropy by a simple change of their orientation in space, reversing their donor–acceptor properties because the oxygen lone pair loses conjugation with the π-system. Instead, the C–O σ* antibonding molecular orbital (MO) can mix with the MOs of the π-system and diminish its electron density effectively serving as an acceptor.^62,63^ We tested this notion in a kinetic competition experiment by mixing 2 equiv of **3a** and **3b** each and 6 equiv of **4a** in CDCl_3_ and monitoring the reaction progress by ^1^H NMR (Figure 3). While **3b** was consumed entirely within 2 h of the reaction, traces of **3a** were still present after 6 h. This agrees with the kinetic profiles of **1a** and **1b** measured independently (Figure S57). Small amounts of **1a** and **1b** were already detected after 2 h together with a product that we assigned to a cage having both type of bridges (**1ab**, see Figure 3 and Scheme S1). Attempts to isolate **1ab** from the mixture by rGPC failed because hydrodynamic radii of **1a**, **1b**, and **1ab** are very similar. Early on, the number of formed cages followed the number of **3b** incorporated in the cage: **1b** > **1ab** > **1a**. In addition, a set of four resonances (9.60–9.75 ppm) suggests the presence of macrocyclic [2 + 2] condensates **5** (Figure 3) that we tentatively assigned following previously identified **5a** observed upon partial hydrolysis of **1a** (Figures S54 and S55).^28^ Further evidence corroborating their presence was obtained by HRMS analysis of the reaction mixture (Figure S56). Their concentration remained low during the 120 h experimental window, except for **5b** (Figures S51–S53). The concentration of all cages steadily increased over time, but only that of **1b** reached a maximum (∼24 h), after which it slowly decomposed with concomitant increase of **5b** (Figures S52 and S53). Cages **1a** and **1ab** remained stable until the end of the experiment (Figure S52). The same observation can be made when **3a** and **3b** are used in excess to **4a** (Figures S58 and S59), although the rate of formation of **1a** from the moment **1b** starts decomposing is higher than that of **1ab**. The reaction mixture turned slightly turbid after 48 h, likely due to the formation of insoluble oligomers. Independent samples of **1a** and **1b** in dry or wet CDCl_3_ (Figure S69) did not show any sign of cage decomposition in 30 days.^28^ A few key conclusions can thus be drawn: (i) **3b** is indeed more electrophilic than **3a**, (ii) a single MeO bridge does not compromise the cage stability, and (iii) decomposition of **1b** to **5b** must be catalyzed. It follows from (ii) that cage hydrolysis does not proceed via removing the bridge, but by opening a cage rung and releasing **4a**, i.e., the [2 + 2] macrocycles **5** are intermediates in cage formation.^28^

**Figure 4:**
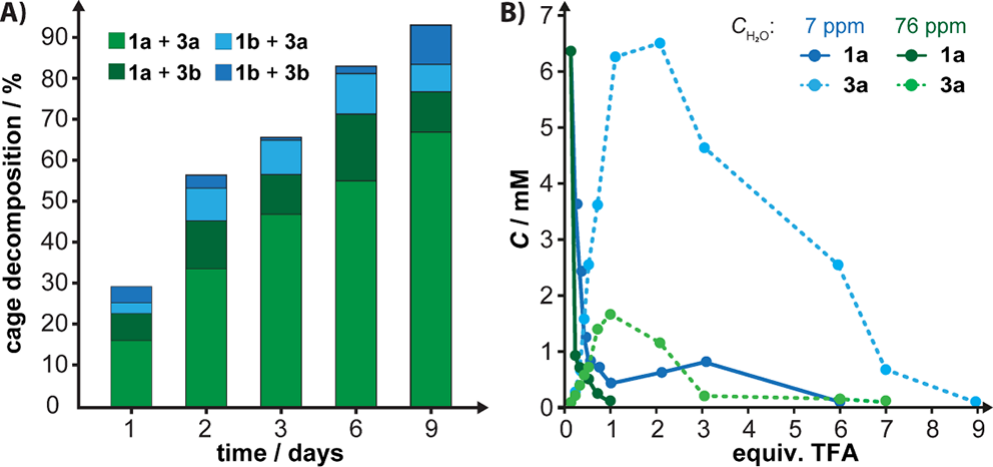
Cage decomposition in CDCl_3_ upon the addition of (A) **3** or (B) 2,2,2-trifluoroacetic acid.

The corresponding decomposition process requires hydrolyzing only two imine bonds, which has been observed previously by Wagner and coworkers for dynamic salicylimine cubes that underwent quadruple catenation.^44^ We probed if the aldehydes present in the solution could cap a free amine after one of the cage imines gets hydrolyzed, preventing it from reforming the cage. Thereby, aldehydes would serve as activators, increasing the overall rate of cage decomposition, formally releasing **4a**–aldehyde condensates that might be dynamic due to the absence of a rung. We compared the reaction progress of solutions of **1a** and **1b** upon the addition of 6 equiv of **3a** or **3b** in CDCl_3_ (Figure 4A, *c*_H2O_ ∼ 70 ppm, 25 °C). We observed that in all cases, the cages decomposed over the course of days, but all at different relative rates: **1b**, **3b** ≈ **1b**, **3a** > **1a**, **3b** > **1a**, **3a**. The observed trend reinforces that **3b** is more electrophilic than **3a** and confirms that **1a** is kinetically more stable than **1b** because it incorporates two bridges from a less electrophilic trialdehyde. The decomposition also proceeds with a catalytic amount of **3**, although at a slower rate (Figures S63–S65). We do not observe the formation of **1ab** when **1a** (or **1b**) was reacted with **3b** (**3a**) irrespective of the water content in CDCl_3_ (Figures S66–S68). We only observe the expected formation of **5a** that is continuously converted to **5b** and **5ab** (Figures S65–S67, see Figure 3 or Scheme S1 for the structure of **5ab**). The excess of aldehydes scavenges released **4a**, preventing cage reformation under the reaction conditions. Increasing the temperature to 60 °C and the addition of TFA to a water-saturated CHCl_3_ solution of **1a** with excess of **3a**-*d*_3_ was necessary to observe a slow formation of **1a**-*d*_3_ as revealed by MS analysis (Figure 2). Cage **1a** decomposes by a stepwise addition of TFA (probed at ∼10 min after the addition; Figure 4B) that promotes DCC. TFA initiated the formation of **5a** and additional **4a**-aldehyde condensates that we did not identify. Their concentration was relatively independent of the added TFA (<2 equiv); however, that of **1a** gradually decreased (Figures S70–S71). Simultaneously, the concentration of **3a** increased, reaching a maximum at ∼1–2 equiv of TFA before gradually declining. Consumption of **3a** correlated with the formation of precipitates, suggesting its incorporation into insoluble oligomers. The reaction progress critically depended on water. Under dry conditions (7 ppm of H_2_O), a catalytic amount of TFA did not fully decompose **1a** (even after 24 h, Figure S72) and produced **3a** at a higher concentration than in the water-enriched (76 ppm) sample. A few equivalents of TFA were necessary to fully consume **1a** in dry CDCl_3_, while the process appeared to be catalytic at higher water content. In **1b**, 0.1 equiv of TFA resulted in its complete decomposition, even under anhydrous conditions (Figure S73). These experiments underscore the unusual kinetic stability of **1a** and show that alkoxy groups in **3** may protect a cage kinetically only in a solid sample, i.e., preventing such a cage from being reached by water and acid/base catalysts, unlike in a homogeneous solution where the cage is fully available to nucleophiles and catalysts. We also argue that water concentration, an often underestimated parameter in POC synthesis, may critically affect reaction kinetics and possibly establish the kinetic or thermodynamic control of the process.

## Conclusion

In conclusion, we demonstrated that [2 + 3] rylene diimide cages are kinetically trapped, yet formed in high yields despite the absence of the error-correction mechanism of imine DCC. We showed how their rate of formation and kinetic stability were affected by the electronic and steric properties of the substituted bridge, the presence of aldehyde or acid catalysts, or the presence of water. This helped us identify [2 + 2] macrocyclic intermediates in their formation/decomposition. These macrocycles might equally be kinetically trapped, representing exciting targets to investigate as key intermediates to accomplish the synthesis of unprecedented kinetically trapped imine assemblies in high yields and selectivity. This study thus uncovers factors that may critically govern their formation and stability, which will inform the design of future three-dimensional imine systems locked out-of-equilibrium.

## Experimental Section

### General Information

All reactions with reagents that are easily oxidized or hydrolyzed were performed under argon (Ar) using Schlenk techniques with anhydrous solvents in glassware that was dried prior to use. NMR experiments were performed on Bruker Avance III NMR spectrometers operating at 300, 400, 500, or 600 MHz proton frequencies. The chemical shifts (δ) are reported in ppm relative to the residual solvent peak, and the coupling constants (*J*) are given in Hz (±0.1 Hz). FTIR spectroscopy was performed by using a PerkinElmer Frontier spectrometer. IR spectra were recorded in four scans at a resolution of 1 cm^–1^. A Shimadzu LC-20AT HPLC instrument was used, equipped with a diode-array UV/vis detector (SPDM20A VP from Shimadzu, *l* = 300–450 nm) and a Shimadzu CTO-20AC column oven at 25 °C. A Chiralpak IA column, 5 μm, 4.6 × 250 mm by Daicel Chemical Industries Ltd., was used for purification. High-resolution mass spectra (HR-MS) measurements were performed on a maXisTM 4G instrument from Bruker. The previously reported compounds (1*R*,2*R*)-cyclohexane-1,2-diamine,^64^ 1,3,5-triformyl benzene^65,66^ (**3a**), 2,4,6-trimethoxybenzene-1,3,5-tricarbaldehyde^67^ (**3b**), 2,4,6-tribromobenzene-1,3,5-tricarbaldehyde,^67^ diamine **4a**,^68^ diamine **4b**,^68^ cage **1a**,^28,29^ 1,3,5-tributoxybenzene,^69^ and 1,3,5-tris(bromomethyl)-2,4,6-tributoxybenzene^8^ were prepared following the reported procedures.

### Procedures for the Synthesis of Bridging Trialdehydes **3**

#### 1,3,5-Tris(hydroxy(^2^H_6_)methyl)benzene

The compound was synthesized previously.^65,67^ A solution of trimethyl-1,3,5-benzenetricarboxylate (0.81 g, 3.70 mmol) in anhydrous THF (10 mL) was added dropwise to a solution of LiAl^2^H_4_ (0.50 g, 11.70 mmol, 98 atom % ^2^H) in anhydrous THF (8 mL) at 0 °C. The mixture was allowed to warm up to room temperature under stirring and then stirred under reflux overnight. Afterward, water (50 mL) was added carefully. The heterogeneous mixture was filtered, and the filtrate was concentrated under reduced pressure. The product 1,3,5-tris(hydroxy(^2^H_6_)methyl)benzene was isolated as a white powder (545 mg, 3.08 mmol, 96%). ^1^H NMR (400 MHz, MeOD, 25 °C): δ = 7.29 (s, 6H); ^13^C{^1^H} NMR (75 MHz, MeOD, 25 °C): δ = 140.9, 125.7, and 63.2 ppm. HR-EI-MS *m*/*z*: [*M*]^+^, calcd. for C_9_H_6_D_6_O_3_: 174.1171; found: 174.1163.

#### 1,3,5-Tri(^2^H_3_)formylbenzene (**3a**-*d*_3_)

The conditions for **3a**-*d*_3_ were adapted from previous literature reports.^65,67^ 1,3,5-tris(hydroxy(^2^H_6_)methyl)benzene (0.55 g, 3.08 mmol) and Dess–Martin periodinane (4.65 g, 11.00 mmol) were suspended in anhydrous CH_2_Cl_2_ (50 mL) and stirred vigorously for 15 h. Diethyl ether (50 mL) was added, and the solution was washed with a solution of Na_2_S_2_O_3_ (12.4 g, 50 mmol) in sat. NaHCO_3_ (50 mL) followed by washing with sat. NaHCO_3_ solution (50 mL). The combined aqueous layers were extracted with CH_2_Cl_2_ (3 × 50 mL). The combined organic layers were then dried with Na_2_SO_4_ and the solvent was removed under reduced pressure to afford a slightly yellow powder. The crude product was purified in small portions by automated flash column chromatography (SiO_2_; cyclohexane:EtOAc, 60:40). 1,3,5-Tri(^2^H_3_)formylbenzene was isolated as a white solid (380 mg, 2.30 mmol, 73%). ^1^H NMR (500 MHz, CDCl_3_, 25 °C): δ 10.21 (s, 3H, residual ^1^H resonance >1%), 8.65 ppm (s, 3H); ^13^C{^1^H} NMR (126 MHz, CDCl_3_, 25 °C): δ 189.9, 137.9, 134.9 ppm. HR-EI-MS: *m*/*z* (%): 165.0505 (100, [*M*]^+^, calcd. for C_9_H_3_D_3_O_3_: 174.1171), 163.0380 (90, [*M*]^+^, calcd. for C_9_H_5_D_1_O_3_: 163.0364); HR-EI-MS *m*/*z*: [*M*]^+^, calcd. for C_9_H_3_D_3_O_3_: 165.0505; found: 165.0504, [M]^+^, calcd. For C_9_H_5_D_1_O_3_: 163.0380; found: 163.0364. IR (ATR, cm^–1^): ν_max_ = 3057, 2142, 2096, 1687, 1663, 1592, 1442, 1265, 1160, 1061, 943, 912, 816, 737, 667, 631, and 473.

#### 2,4,6-Triethylthioether-1,3,5-tricarbaldehyde

Under an inert atmosphere, sodium ethanethiolate (655 mg, 7.78 mmol) and 2,4,6-tribromobenzene-1,3,5-tricarbaldehyde^67^ (1.00 g, 2.51 mmol) were dissolved in DMF (25 mL) at 0 °C. The resulting mixture was stirred at room temperature for 24 h, and subsequently, Et_2_O (10 mL) was added. The precipitating salts were separated, and the filtrate was evaporated to dryness to afford 2,4,6-triethylthioether-1,3,5-tricarbaldehyde as a yellow powder (143 mg, 0.34 mmol, 81%). ^1^H NMR (500 MHz, CDCl_3_, 25 °C): δ 10.50 (s, 3H), 2.85 (*q*, *J* = 7.4 Hz, 6H), and 1.21 ppm (*t*, *J* = 7.4 Hz, 9H). ^13^C{^1^H} NMR (126 MHz, CDCl_3_, 25 °C): δ 191.4, 147.4, 137.3, 33.8, and 14.6 ppm. HR-EI-MS *m*/*z*: [*M*]^+^, calcd. for C_15_H_18_O_3_S_3_: 342.0418; found: 342.0426, [*M* – C_2_H_6_]^+^, calcd. for C_13_H_13_O_3_S_3_: 313.0027; found: 313.0030. IR (ATR, cm^–1^): ν_max_ = 2965, 2929, 2864, 1694, 1518, 1438, 1375, 1263, 1056, 991, 974, 944, 783, 667, 588, 537, and 500.

#### (2,4,6-Tributoxybenzene-1,3,5-triyl)tris(methylene) Triacetate

1,3,5-Tris(bromomethyl)-2,4,6-tributoxybenzene^8^ (8.48 g, 14.8 mmol) was dissolved in glacial acetic acid (180 mL), followed by the addition of sodium acetate (21.7 g, 221.0 mmol). The mixture was heated to reflux for 16 h. After the mixture was cooled to 25 °C, the suspension was poured into CH_2_Cl_2_ (200 mL) and filtered. The crude product was dissolved in ethyl acetate (400 mL), washed with saturated aq. NaHCO_3_ solution, water, and brine, and dried with Na_2_SO_4_. The solvent was removed under reduced pressure, and the crude mixture was purified by flash column chromatography (SiO_2_, cyclohexane/EtOAc 3:1) to obtain (2,4,6-tributoxybenzene-1,3,5-triyl)tris(methylene) triacetate as a white solid (7.3 g, 14.2 mmol, 96%). ^1^H NMR (500 MHz, CDCl_3_, 25 °C): δ 5.13 (s, 6H), 3.89 (*t*, *J* = 7.5 Hz, 6H), 2.07 (s, 9H), 1.78–1.72 (m, 6H), 1.49–1.41 (m, 6H), and 0.96 ppm (*t*, *J* = 7.5 Hz, 9H). ^13^C{^1^H} NMR (126 MHz, CDCl_3_, 25 °C): δ 170.9, 161.5, 119.8, 57.3, 32.3, 21.2, 19.3, and 14.0 ppm.

#### 2,4,6-Tributoxy-1,3,5-benzenetrimethanol

(2,4,6-Tributoxybenzene-1,3,5-triyl)tris(methylene) triacetate (6.99 g, 13.7 mmol) was dissolved in ethanol (80 mL), and aq. NaOH (8.21 g, 205 mmol, 80 mL of water) was added. The mixture was refluxed for 16 h and cooled to room temperature; ethanol was removed under reduced pressure, and the remaining aqueous mixture was neutralized with 1 M aq. HCl and concentrated under reduced pressure. The crude solid was washed with EtOAc (2 × 100 mL) and filtered. The filtrate was dried with Na_2_SO_4_ and concentrated under reduced pressure to obtain (2,4,6-tributoxybenzene-1,3,5-triyl)trimethanol as a white solid (5.00 g, 13.0 mmol, 95%). ^1^H NMR (500 MHz, DMSO-*d*_6_, 25 °C): δ 4.59 (*t*, *J* = 5.0 Hz, 3H), 4.45 (*d*, *J* = 5.0 Hz, 6H), 4.01 (*t*, *J* = 5.0 Hz, 6H), 1.76–1.71 (m, 6H), 1.52–1.45 (m, 6H), and 0.95 ppm (*t*, *J* = 7.5 Hz, 9H). ^13^C{^1^H} NMR (126 MHz, DMSO-*d*_6_, 25 °C): δ 158.3, 124.3, 75.8, 53.2, 31.9, 18.7, and 13.9 ppm.

#### 2,4,6-Tributoxybenzene-1,3,5-tricarbaldehyde

2,4,6-Tributoxy-1,3,5-benzenetrimethanol (6.00 g, 15.6 mmol) was dissolved in dry CH_2_Cl_2_ (120 mL) and Dess-Martin periodinane (26.40 g, 62.3 mmol) was added. The resulting suspension was stirred under an Ar atmosphere for 16 h. Next, the reaction mixture was diluted with Et_2_O (100 mL) and quenched with a solution of Na_2_S_2_O_3_ (13.8 g, 87.5 mmol) in sat. aq. NaHCO_3_ solution (100 mL). The organic phase was separated and washed with saturated aq. NaHCO_3_ solution (100 mL). The aqueous layers were extracted with CH_2_Cl_2_ (3 × 100 mL). The combined organic layers were dried with Na_2_SO_4_, and concentrated to obtain a brown, oily residue. The crude mixture was purified by column flash chromatography (SiO_2_, cyclohexane/EtOAc 1:1) to afford the pure 2,4,6-tributoxybenzene-1,3,5-tricarbaldehyde as a colorless oil (2.4 g, 10.9 mmol, 70%). ^1^H NMR (500 MHz, CDCl_3_, 25 °C): δ 10.33 (s, 3H), 4.08 (*t*, *J* = 7.5 Hz, 6H), 1.86–1.80 (m, 6H), 1.50–1.43 (m, 6H), and 0.96 (*t*, *J* = 7.5 Hz, 9H). ^13^C{^1^H} NMR (126 MHz, CDCl_3_, 25 °C): δ 187.5, 169.1, 120.3, 79.1, 32.1, 19.1, and 13.9 ppm; IR (ATR, cm^–1^): ν_max_ = 2925, 2855, 1733, 1589, 1447, 1377, 1354, 1222, 1118, 1019, 950, 799, 723, 636, 605, 594, 558, and 453.

### General Procedure for the Synthesis of Cages

Diamine **4** (1 equiv) was suspended in a solution of the appropriate aldehyde (0.66 equiv) in dry CHCl_3_. The mixture was then stirred at room temperature for 48 h, after which the solution was filtered through a disposable syringe filter. The filtrate was concentrated under reduced pressure to obtain a solid crude product. A small amount of MeOH was added, and the suspension was sonicated at room temperature for 30 min and filtered. The filter cake was washed with additional MeOH followed by a portion of diethyl ether and dried in the air. The crude product was purified by HPLC using CH_2_Cl_2_/*n*-heptane (7:3) as eluent to yield a pure cage.

#### Cage **1b**

According to the general procedure, **1b** was prepared from 2,4,6-trimethoxybenzene-1,3,5-tricarbaldehyde **3b** (77 mg, 0.30 mmol) and (*R*)-**4a** (210 mg, 0.46 mmol) in dry CHCl_3_ (46 mL) as a pale yellowish solid (130 mg) in 48%. ^1^H NMR (500 MHz, CDCl_3_, 25 °C): δ 8.64 (*d*, *J* = 10.0 Hz, 6H), 8.58 (*d*, *J* = 10.0 Hz, 6H), 8.04 (s, 6H), 5.43–5.38 (m, 6H), 4.21–4.16 (m, 6H), 3.56 (s, 18H), 2.69–2.62 (m, 6H), 1.93–1.91 (m, 6H), 1.84–1.79 (m, 12H), 1.73–1.71 (m, 6H), 1.68–1.63 (m, 6H), 1.63–1.58 (m, 6H), and 1.50–1.48 ppm (m, 6H). ^13^C{^1^H} NMR (126 MHz, CDCl_3_, 25 °C): δ 163.4, 163.1, 160.9, 154.8, 131.2, 130.8, 127.0, 126.5, 126.4, 122.0, 69.8, 64.2, 58.1, 34.9, 27.3, 25.9, and 24.3 ppm. HR-EI-MS *m*/*z*: [*M* + 2H]^2+^, calcd. for C_102_H_98_N_12_O_18_: 889.3556; found: 889.3564, [*M*]^+^, calcd. for C_102_H_97_N_12_O_18_: 1777.7038; found: 1777.7009. Mp (°C): > 300, decomposition before melting. IR (ATR, cm^–1^): ν_max_ = 2927, 2856, 1703, 1662, 1579, 1558, 1451, 1372, 1324, 1258, 1244, 1215, 1189, 1124, 1098, 1003, 976, 877, 812, 768, 731, 634, 582, 502, 477, and 457.

#### Cage **2b**

According to the general procedure, **2b** was prepared from 2,4,6-trimethoxybenzene-1,3,5-tricarbaldehyde **3b** (87 mg, 0.35 mmol) and (*R*)-**4b** (221 mg, 0.52 mmol) in dry CHCl_3_ (52 mL) as a white solid (245 mg, 0.30 mmol, 85%). ^1^H NMR (500 MHz, CDCl_3_, 25 °C): δ 8.17 (s, 6H), 8.08 (s, 6H), 8.04 (s, 6H), 4.49–4.43 (m, 6H), 3.85–3.82 (m, 6H), 3.81 (s, 18H), 2.48–2.39 (m, 6H), 1.92–1.89 (m, 6H), 1.81–1.79 (m, 12H), 1.74–1.71 (m, 6H), 1.67–1.57 (m, 6H), 1.52–1.47 (m, 6H), and 1.44–1.39 ppm (m, 6H). ^13^C{^1^H} NMR (126 MHz, CDCl_3_, 25 °C): δ 166.6, 165.6, 161.1, 155.2, 137.1, 136.6, 122.1, 118.0, 70.3, 64.4, 56.0, 34.3, 28.9, 25.5, and 24.0 ppm. HR-EI-MS *m*/*z*: [*M* + 2H]^2+^, calcd. for C_90_H_92_N_12_O_18_: 814.8333; found: 814.8342, [*M*]^+^, calcd. for C_90_H_92_N_12_O_18_: 1628.6653; found: 1628.6567. Mp (°C): > 300, decomposition before melting (see Figures S75 and S76). IR (ATR, cm^–1^): ν_max_ = 2926, 1769, 1706, 1635, 1564, 1451, 1344, 1196, 1154, 1092, 1003, 936, 851, 823, 728, 631, 562, and 502.

#### Cage **1a-***d*_**6**_

According to the general procedure, **1a**-*d*_6_ was prepared from 1,3,5-tri(^2^H_3_)formylbenzene (33.0 mg, 0.30 mmol) and (*R*)-**4a** (138 mg, 0.30 mmol) in dry CHCl_3_ (46 mL) as a pale yellowish solid (128.0 mg, 0.08 mmol, 79%). ^1^H NMR (500 MHz, CD_2_Cl_2_, 25 °C): δ 8.62 (*d*, *J* = 7.6 Hz, 6H), 8.52 (*d*, *J* = 7.6 Hz, 7H), 7.70 (s, 6H), 5.34 (ddd, *J* = 12.6, 10.1, 4.1 Hz, 6H), 4.35 (*q*, *J* = 8.3 Hz, 6H), 2.50–2.40 (m, 6H), 1.91–1.44 ppm (m, 24H). ^13^C{^1^H} NMR (126 MHz, CDCl_3_, 25 °C): δ 163.9, 163.7, 137.5, 131.5, 130.8, 129.2, 127.5, 127.0, 126.8, 69.3, 59.2, 36.3, 29.6, 26.4, 24.9, 21.61 ppm. HR-EI-MS *m*/*z*: [*M* + 3H]^3+^, calcd. for C_96_H_81_D_6_N_12_O_12_: 535.2321; found: 535.2309, [*M* + 2H]^2+^, calcd. for C_96_H_80_D_6_N_12_O_12_: 802.3436; found: 802.3427, [*M* + H]^+^, calcd. for C_96_H_79_D_6_N_12_O_12_: 1603.6288; found: 1603.6288. Mp (°C): > 300, decomposition before melting. IR (ATR, cm^–1^): ν_max_ = 2927, 1706, 1665, 1580, 1453, 1325, 1260, 1246, 1217, 1190, 1103, 977, 880, 769, 734, 686, 635, 584, and 472.

#### Cage **1d**

According to the general procedure, **1d** was prepared from aldehyde **3d** (37.3 mg, 0.0986 mmol) and **4a** (68 mg, 0.148 mmol) in dry CHCl_3_ (15 mL) as a pale yellowish solid (45 mg, 0.022 mmol, 45%). ^1^H NMR (500 MHz, CDCl_3_, 25 °C): δ 8.59–8.54 (m, 12H), 8.50 (*d*, *J* = 8.3 Hz, 6H), 5.37–5.33 (m, 6H), 4.44 (m, 6H), 3.75 (m, 6H), 3.16 (m, 6H), 2.25–2.21 (m, 6H), 1.85–1.82 (m, 12H), 1.74–1.72 (m, 6H), 1.67–1.60 (m, 12H), 1.54–1.50 (m, 18H), 1.34–1.13 (m, 18H), and 0.94–0.88 ppm (m, 18H). ^13^C{^1^H} NMR (126 MHz, CDCl_3_, 25 °C): δ 163.30, 162.88, 161.99, 156.06, 131.06, 130.38, 127.08, 126.54, 126.40, 120.61, 75.78, 71.66, 59.40, 36.68, 31.61, 28.69, 25.93, 24.62, 19.18, and 14.24 ppm. HR-EI-MS *m*/*z*: [M]^2+^, calcd. for [C_120_H_134_N_12_O_18_]^2+^: 1015.4964; found: 1015.4976. Mp (°C): > 300, decomposition before melting. IR (ATR, cm^–1^): ν_max_ = 2928, 2857, 1704, 1661, 1579, 1452, 1373, 1325, 1258, 1244, 1216, 1188, 1099, 1016, 975, 881, 859, 768, 730, 636, 582, and 474.

## Data Availability

The data underlying this study are available in the published article and its Supporting Information.

## Supporting Information Available

The Supporting Information is available free of charge on the ACS Publications Web site. Experimental procedures, characterization data, computational details, and crystal data (PDF). Structural assignments were made with additional information from gCOSY, gHSQC, and gHMBC experiments”, as described in the “General Methods” of the Experimental Section in the manuscript (PDF)
