# Supplementary material for: Kinetic Trapping of Rylene Diimide Covalent Organic Cages
Source: J Org Chem. 2025 Mar 18;90(12):4158–66. doi: 10.1021/acs.joc.4c02547 (PMC11959530; doi:10.1021/acs.joc.4c02547)
Supplement: Supplementary file 1 — jo4c02547_si_001.pdf [file jo4c02547_si_001.pdf]

Supporting Information for

Kinetic Trapping of Rylene Diimide Covalent Organic Cages

Sergey Fisher[a‡], Hsin-Hua Huang[b‡], Luise Sokoliuk[b], Alessandro Prescimone[b], Olaf Fuhr[c], Tomáš Šolomek*[a,b]

[a] Van 't Hoff Institute for Molecular Sciences, University of Amsterdam, Science Park 904, NL-1098 XH Amsterdam, The Netherlands.

[b] Department of Chemistry, University of Basel, St. Johanns-Ring 19, CH-4056 Basel, Switzerland.

[c] Institute of Nanotechnology and Karlsruhe Nano Micro Facility, Karlsruhe Institute of Technology, Kaiserstraße 12, DE-76131 Karlsruhe, Germany.

Email: t.solomek@uva.nl

Table of Contents

1. Synthesis and characterization.......... S2
1.1. NMR Analysis.......... S5
1.2. High-resolution mass spectrometry analysis.......... S21
1.3. FT-IR analysis.......... S28
2. Crystallographic details.......... S32
3. Exchange experiments.......... S35
3.1 Isotopic purity of deuterated compounds.......... S35
3.2. MALDI MS experiments.......... S35
3.3. ^{1}H NMR experiments.......... S38
3.4. Titration experiments.......... S50
4. Computational methods.......... S53
4.1. Cartesian coordinates.......... S53
4.2. Geometries of bridges.......... S68

1. Synthesis and characterization

General Remarks

All commercially available compounds were purchased from Sigma-Aldrich, Acros, Apollo Scientific, Alfa Aesar or Fluorochem and used without further purification. Anhydrous solvents were purchased from Sigma-Aldrich or Acros and stored over molecular sieves (4 Å). Flash column chromatography was performed on silica gel P60 (40-63 μm) from SilicycleTM, and the solvents were technical grade. Thin-layer chromatography (TLC) was performed with silica gel 60 F254 aluminum sheets with a thickness of 0.25 mm purchased from Merck. All reactions with reagents that are easily oxidized or hydrolyzed were performed under argon (Ar) using Schlenk techniques with anhydrous solvents in glassware, which was dried prior to use. NMR solvents were obtained from Cambridge Isotope Laboratories, Inc. (Andover, MA, USA). NMR experiments were performed on Bruker Avance III NMR spectrometers operating at 300, 400, 500 or 600 MHz proton frequencies. The instruments were equipped with a direct-observe 5 mm BBFO smart probe (400 and 600 MHz), an indirect detection 5 mm BBI probe (500 MHz), or a five-channel cryogenic 5 mm QCI probe (600 MHz). All probes were equipped with actively shielded z-gradients (10 A). The chemical shifts (*δ*) are reported in ppm relative to the residual solvent peak and the coupling constants (*J*) are given in Hz (±0.1 Hz). Standard Bruker pulse sequences were used, and the data was processed on Topspin 3.2 (Bruker) using twofold zero-filling in the indirect dimension for all 2D experiments. FTIR spectroscopy was performed using a PerkinElmer Frontier spectrometer. IR spectra were recorded in four scans at a resolution of 1 cm^{-1}. Each measurement was performed at 25 °C by recording a background. Subsequently, a sample was then placed on the ATR crystal and measured immediately. A Shimadzu LC-20AT HPLC was used equipped with a diode-array UV/vis detector (SPDM20A VP from Shimadzu, l = 300 - 450 nm) and a column oven Shimadzu CTO-20AC at 25 °C. Chiralpak IA column, 5 μm, 4.6 × 250 mm by Daicel Chemical Industries Ltd was used for purification. High-resolution mass spectra (HR-MS) measurements were performed on a maXisTM 4G instrument from Bruker. MALDI-ToF mass spectra were measured on a Bruker microflex™ mass spectrometer, calibrated with CsI_3. A solution of a matrix, anthracene in CH_2Cl_2, was drop-casted onto a sample plate, and a solution of an analyte was drop-casted directly onto the matrix. The previously reported compounds (1*R*,2*R*)-cyclohexane-1,2-diamine[1], 1,3,5-triformyl benzene[2,3] (**3a**), 2,4,6-trimethoxybenzene-1,3,5-tricarbaldehyde[4] (**3b**), 2,4,6-tribromobenzene-1,3,5-tricarbaldehyde[4], diamine **4a**[5], diamine **4b**[5], cage **1a**[6,7], 1,3,5-tributoxybenzene[8], and 1,3,5-tris(bromomethyl)-2,4,6-tributoxybenzene[9] were prepared following the reported procedures.

General Procedure for Method A

Diamine ditosylate salt **4a**·2TsOH (1 equiv) was suspended in anhydrous $CHCl_3$ and the appropriate aldehyde (0.66 equiv) in anhydrous $CHCl_3$ was added in one portion. Subsequently, triethyl amine (7 equiv) was added quickly in one portion to the reaction mixture at room temperature. The mixture was stirred for 48 hours at 80 °C in a pressure vessel. The reaction mixture was allowed to cool down to room temperature and filtered. The filtrate was concentrated under reduced pressure to obtain a solid product. A small amount of methanol was added and the solids were sonicated at room temperature for ~30 minutes and filtered. The filter cake was washed methanol and diethyl ether. Cage **1** was obtained after drying in air.

General Procedure for Method B

A solution of $Sc(OTf)_3$ (0.1 equiv) in CH_3CN (4.0 mg mL^{-1}) was added dropwise to a solution of **4a** (1 equiv) and the appropriate aldehyde (0.66 equiv.) in $CHCl_3$. The yellow solution was stirred at room temperature for 48 h, and the resulting solution was filtered and concentrated to give cage **1**.

General Procedure for Method C

Diamine **4** (1 equiv) was suspended in a solution of the appropriate aldehyde (0.66 equiv) in dry $CHCl_3$. The mixture was then stirred at room temperature for 48 h after which the solution was filtered through a disposable syringe filter. The filtrate was concentrated under reduced pressure to obtain a solid crude product. A small amount of MeOH was added and the suspension was sonicated at room temperature for 30 minutes and filtered. The filter cake was washed with additional MeOH followed by a portion of diethyl ether and dried in air. The crude product was purified by HPLC using CH_2Cl_2/n-Heptane (7:3) as eluent to yield a pure cage.

Table S1. Tested reaction conditions for formation of cages **1b** and **2b**.

Entry	Cage	Conc. / mM	Method	T / °C	Yield[a] / %
1	**1b**[d]	10	A[b]	80	–
2	**1b**	4.5	A	80	–
3	**1b**	10	A	25	< 8
4	**1b**	4.5	A	25	< 8
5	**1b**	4.5	B	25	8
6	**1b**	4.5	B	80	13
7	**1b**	4.5	C	80	26
8	**1b**	4.5	C	25	39
9	**1b**	10	C	80	39
10	**1b**	10	C	25	48
11	**1b**	2	C	80	< 20
12	**1b**	2	C	25	< 20
13[c]	**2b**[e]	10	A	80	20
14[c]	**2b**	10	C	25	85

[a] Isolated yields after purification with HPLC; [b] The reaction time was 14 days; [c] Entries 13 and 14 are for **2b**. [d] Cage **1b** was produced from aldehyde **3b** and diamine **4a**. [e] Cage 2b was produced from aldehyde **3b** and diamine **4b**.

1.1. NMR Analysis

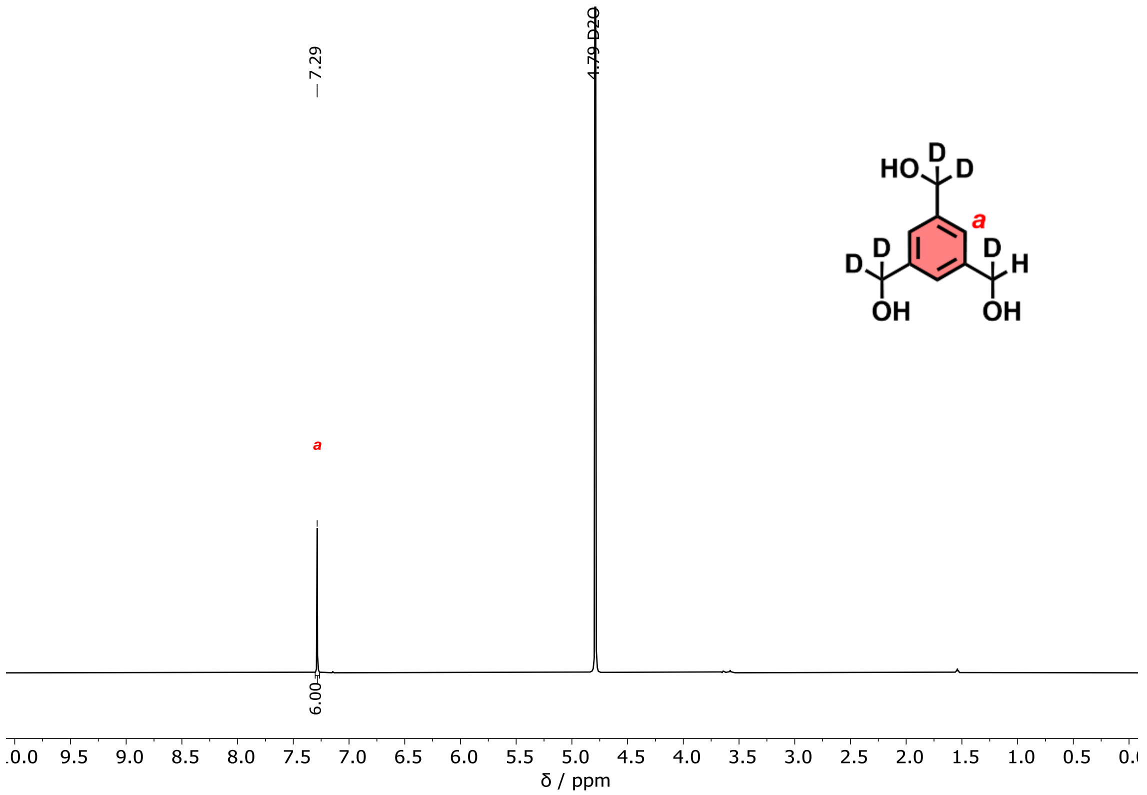

Fig. S1 ^{1}H NMR (500 MHz, D_2O, 298 K) spectrum of 1,3,5-tris(hydroxy($^{2}H_6$)methyl)benzene.

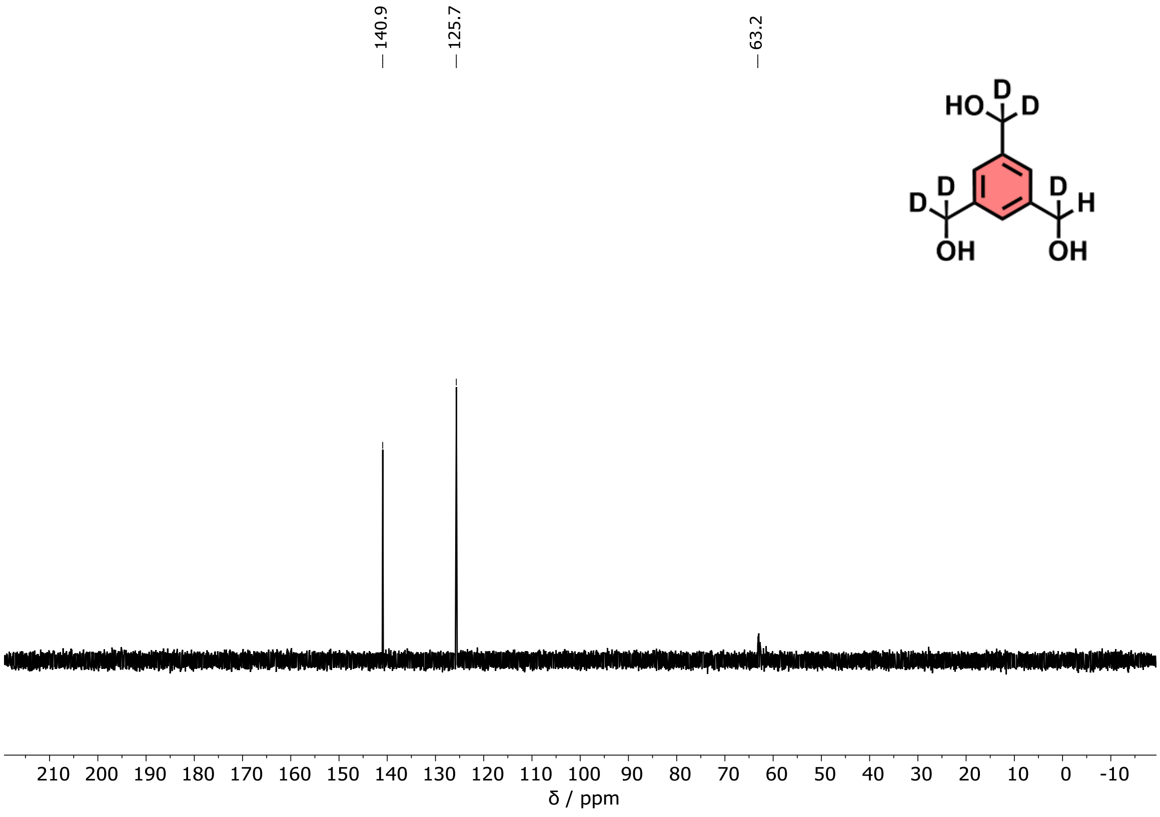

Fig. S2 $^{13}C\{^{1}H\}$ NMR (126 MHz, D_2O, 298 K) spectrum of 1,3,5-tris(hydroxy($^{2}H_6$)methyl)benzene.

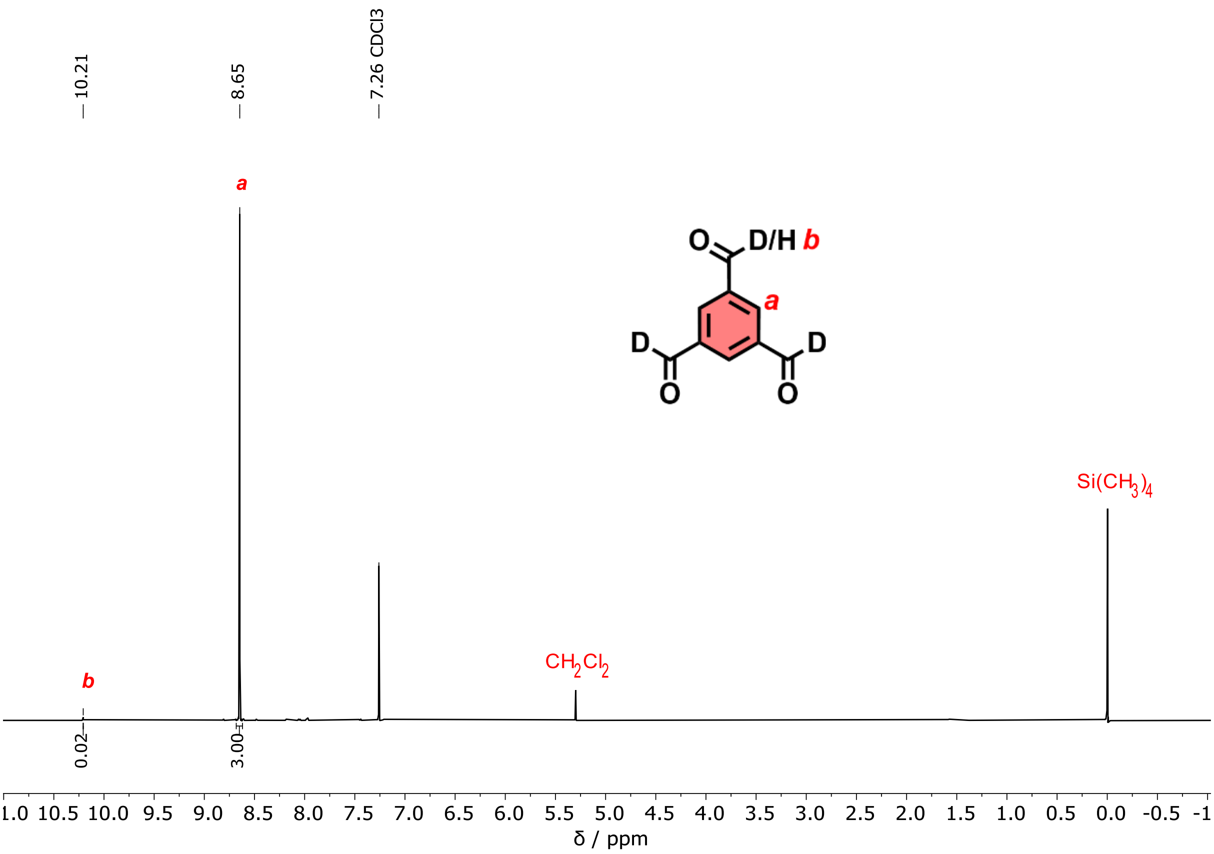

Fig. S3 ^{1}H NMR (500 MHz, $CDCl_3$, 298 K) spectrum of 1,3,5-tri($^{2}H_3$)formylbenzene. Solvent impurities from methylene chloride are highlighted with an asteriks.

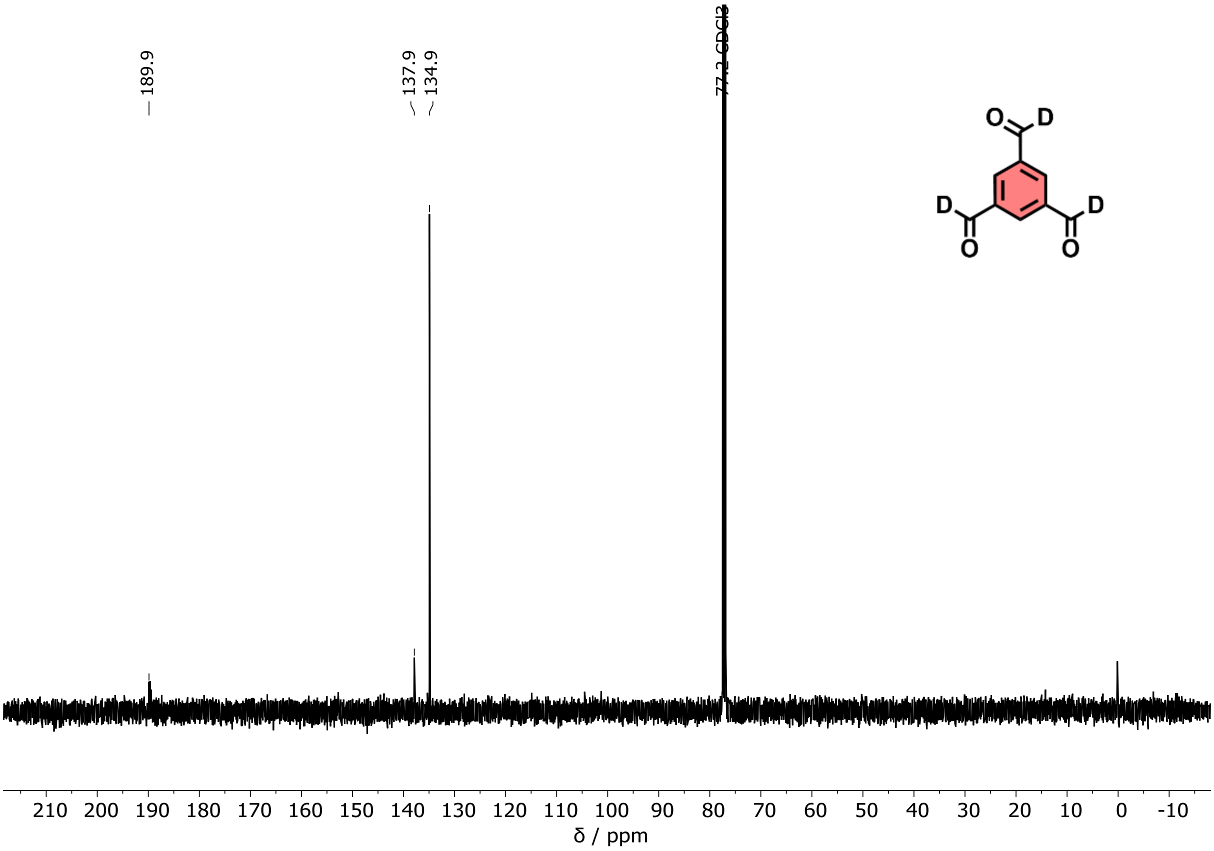

Fig. S4 $^{13}C\{^{1}H\}$ NMR (126 MHz, $CDCl_3$, 298 K) spectrum of 1,3,5-tri($^{2}H_3$)formylbenzene.

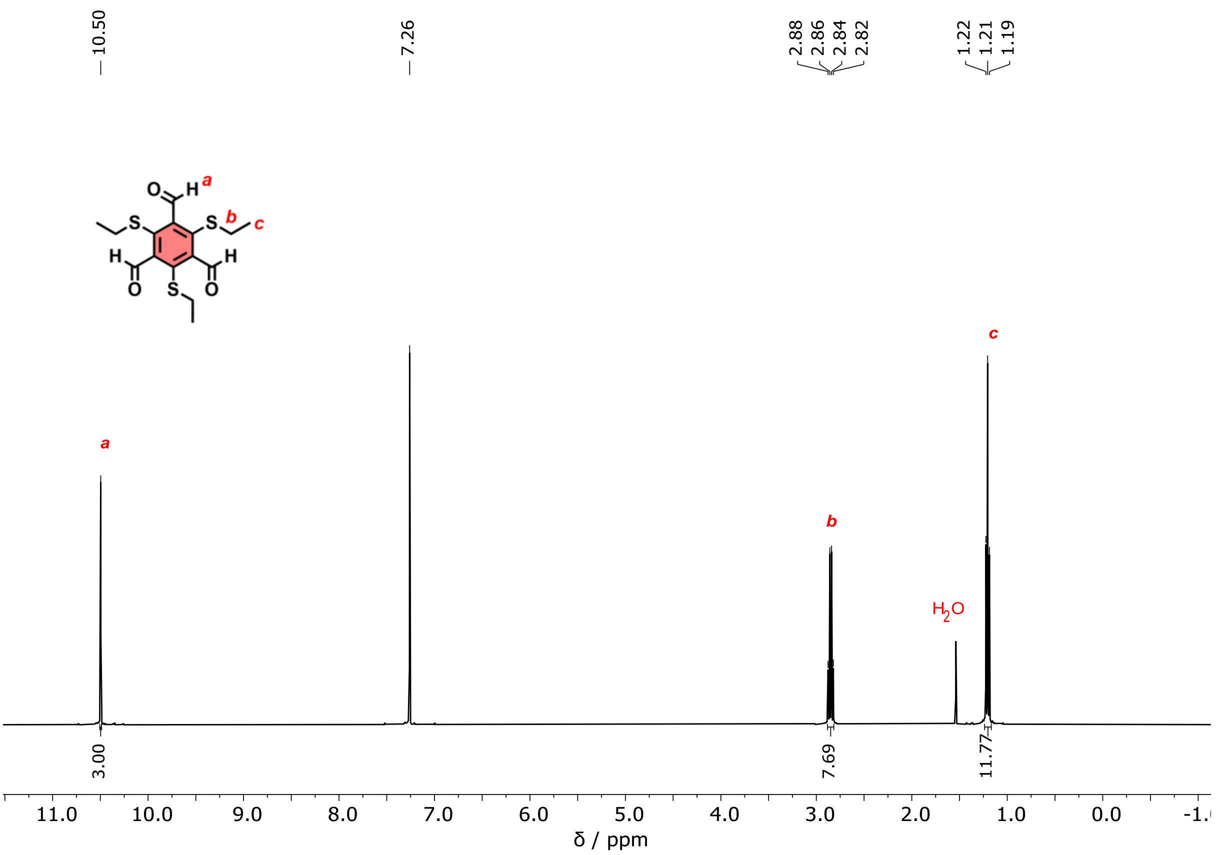

Fig. S5 ^{1}H NMR (500 MHz, $CDCl_3$, 298 K) spectrum of 2,4,6-triethylthioether-1,3,5-tricarbaldehyde.

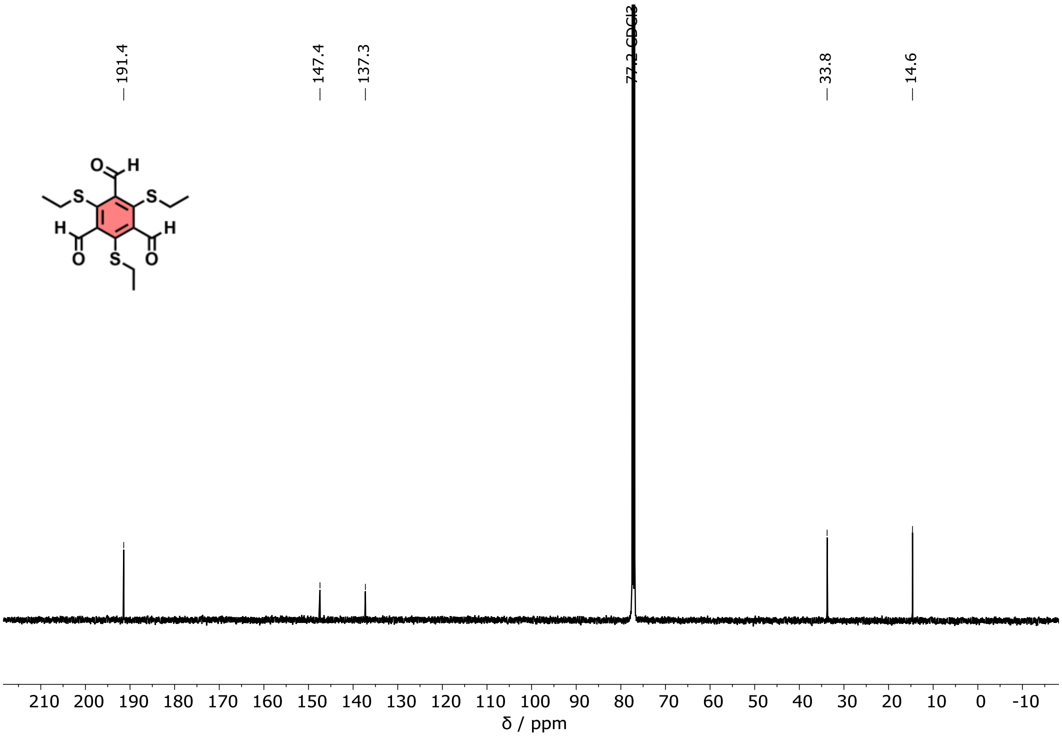

Fig. S6 $^{13}C\{^{1}H\}$ NMR (126 MHz, $CDCl_3$, 298 K) spectrum of 2,4,6-triethylthioether-1,3,5-tricarbaldehyde.

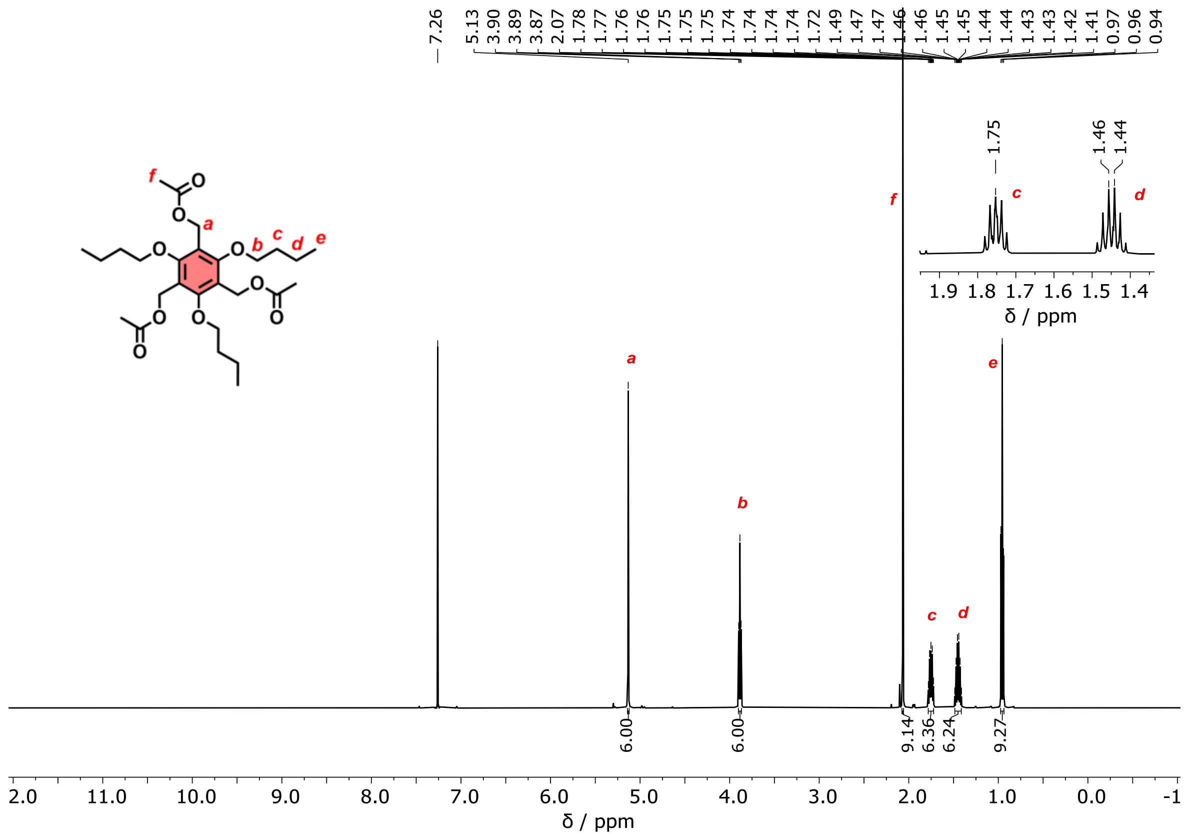

Fig. S7 ^{1}H NMR (500 MHz, $CDCl_3$, 298 K) spectrum of (2,4,6-tributoxybenzene-1,3,5-triyl)tris(methylene) triacetate.

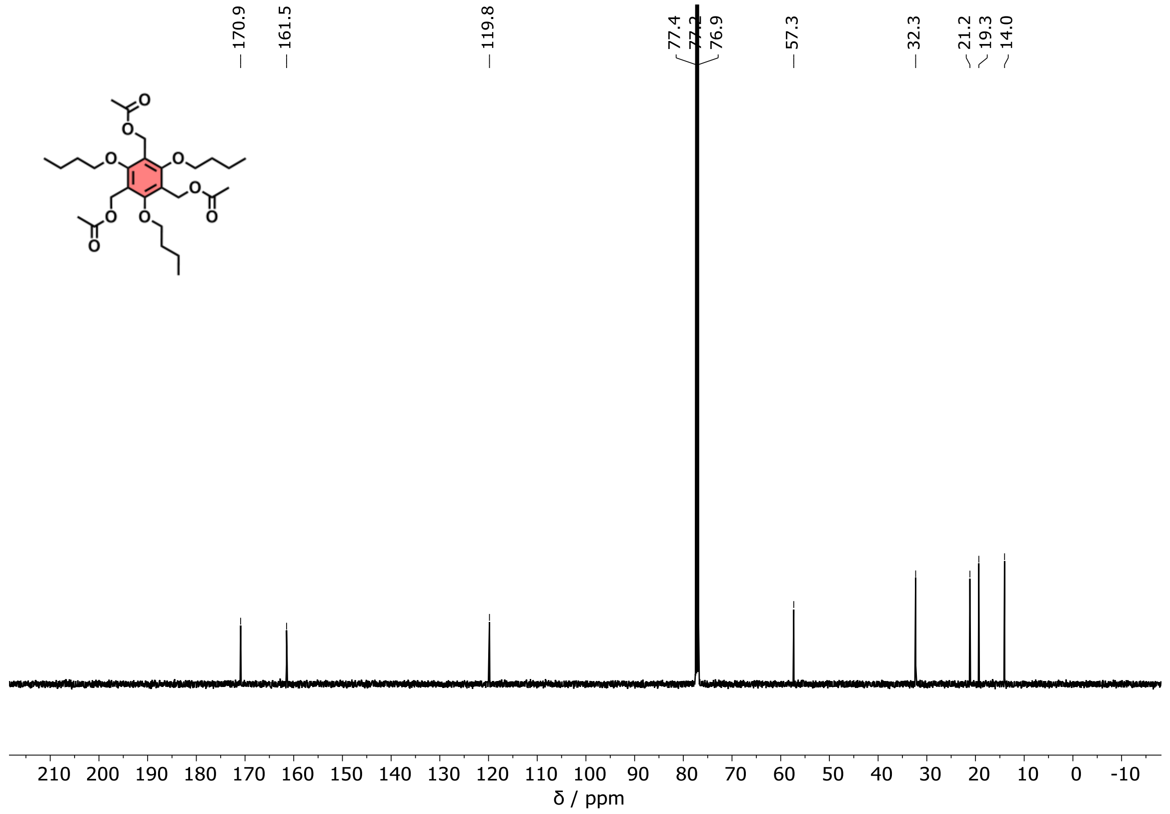

Fig. S8 $^{13}C\{^{1}H\}$ NMR (126 MHz, $CDCl_3$, 298 K) spectrum of (2,4,6-tributoxybenzene-1,3,5-triyl)tris(methylene) triacetate.

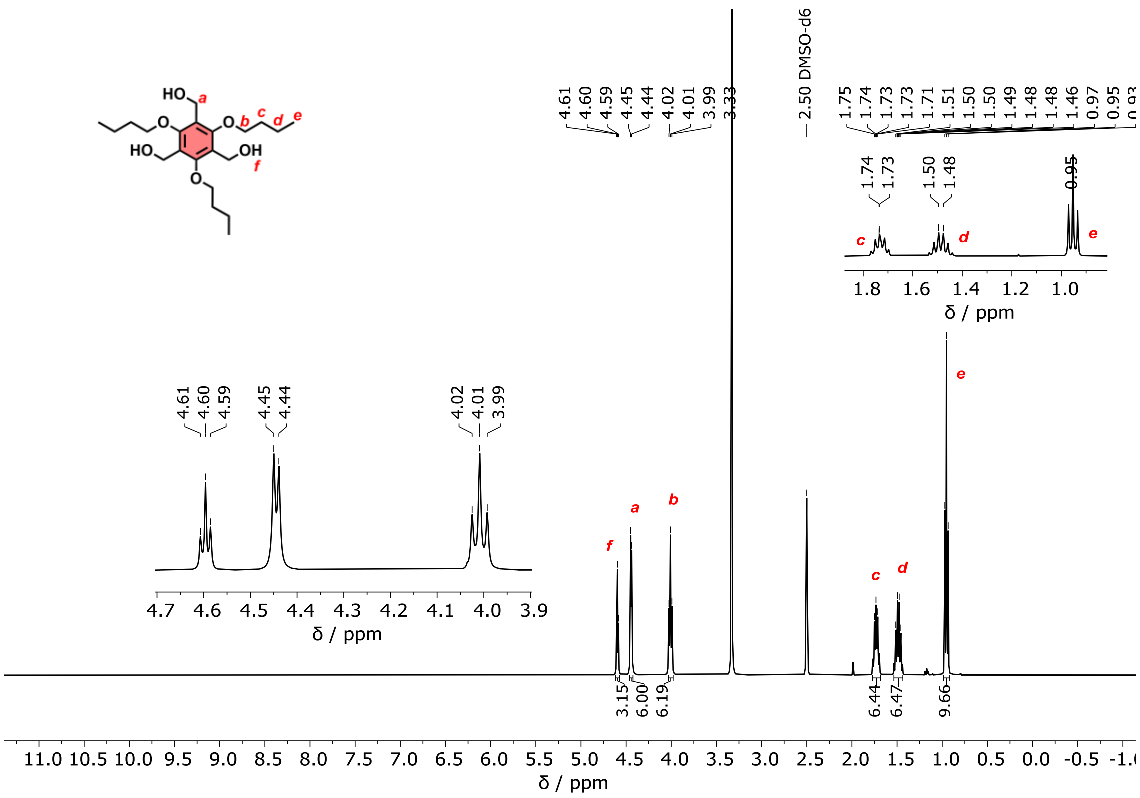

Fig. S9 ^{1}H NMR (500 MHz, DMSO-d_6, 298 K) spectrum of 2,4,6-tributoxy-1,3,5-benzenetrimethanol.

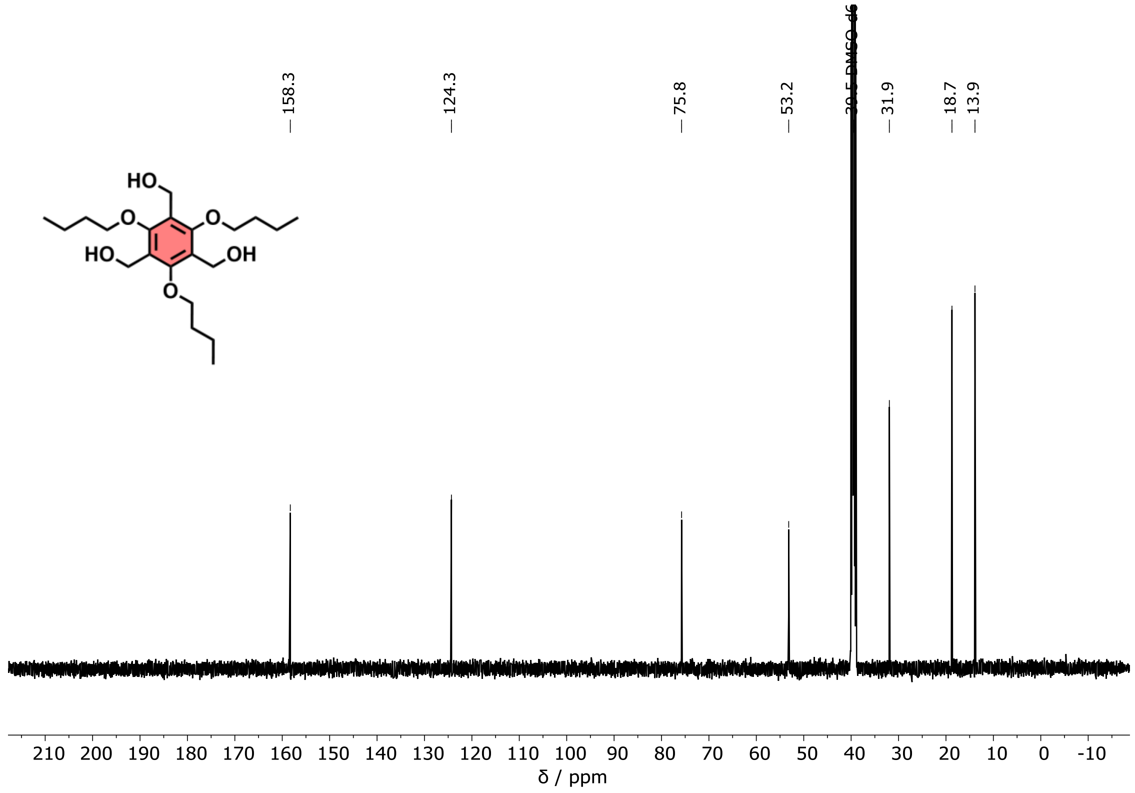

Fig. S10 $^{13}C\{^{1}H\}$ NMR (126 MHz, DMSO-d_6, 298 K) spectrum of 2,4,6-tributoxy-1,3,5-benzenetrimethanol.

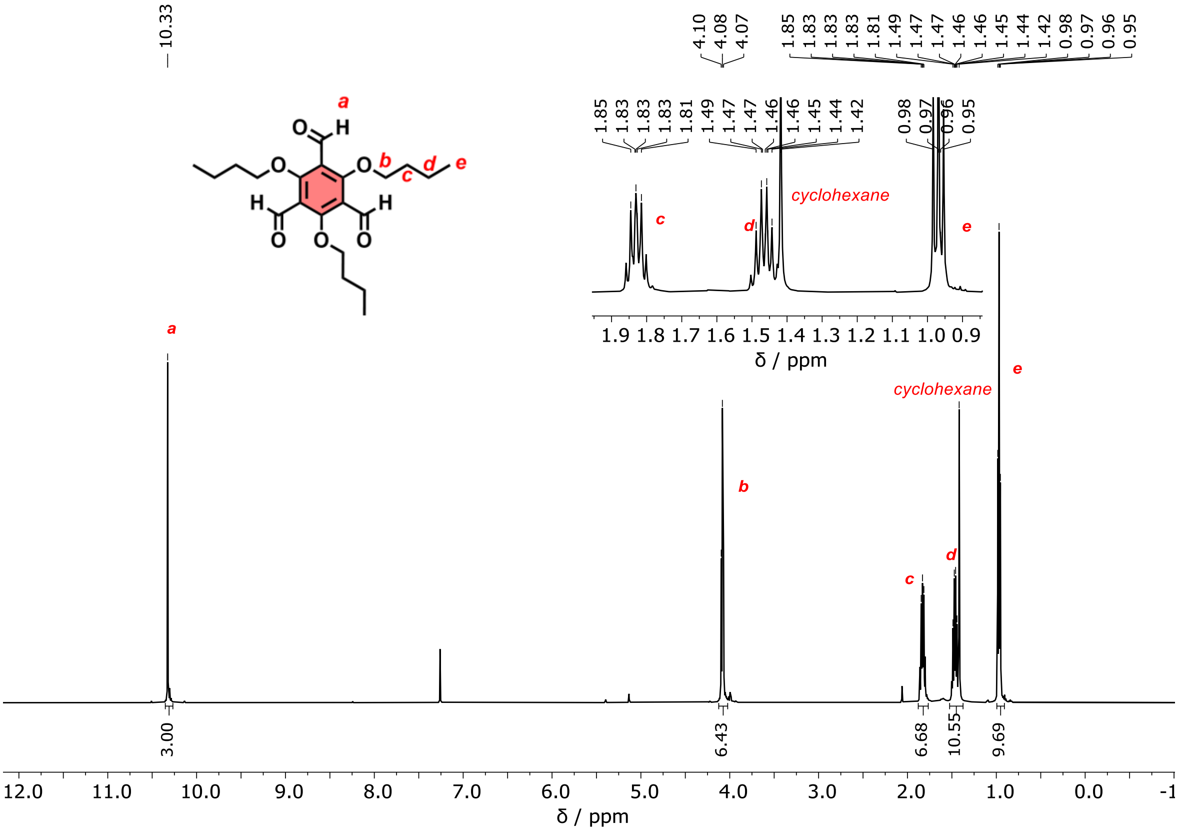

Fig. S11 ^{1}H NMR (500 MHz, $CDCl_3$, 298 K) spectrum of 2,4,6-tributoxybenzene-1,3,5-tricarbaldehyde.

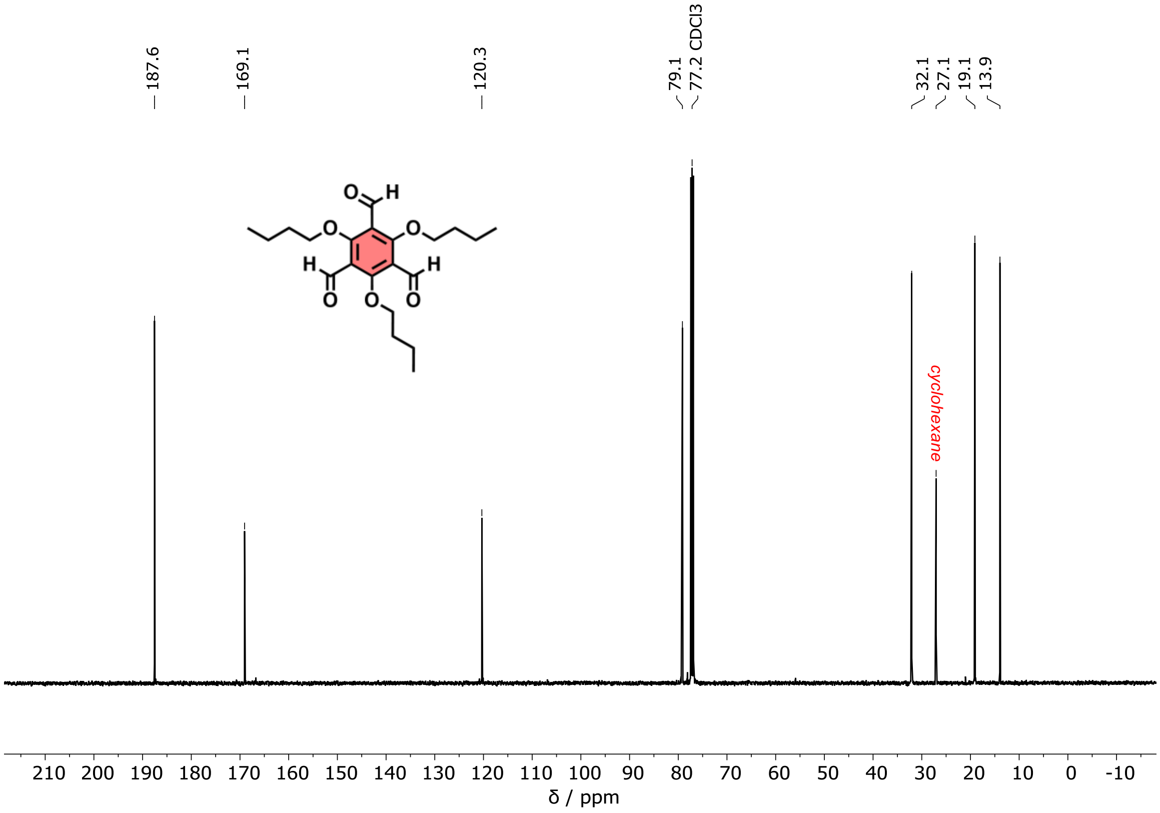

Fig. S12 $^{13}C\{^{1}H\}$ NMR (126 MHz, $CDCl_3$, 298 K) spectrum of 2,4,6-tributoxybenzene-1,3,5-tricarbaldehyde.

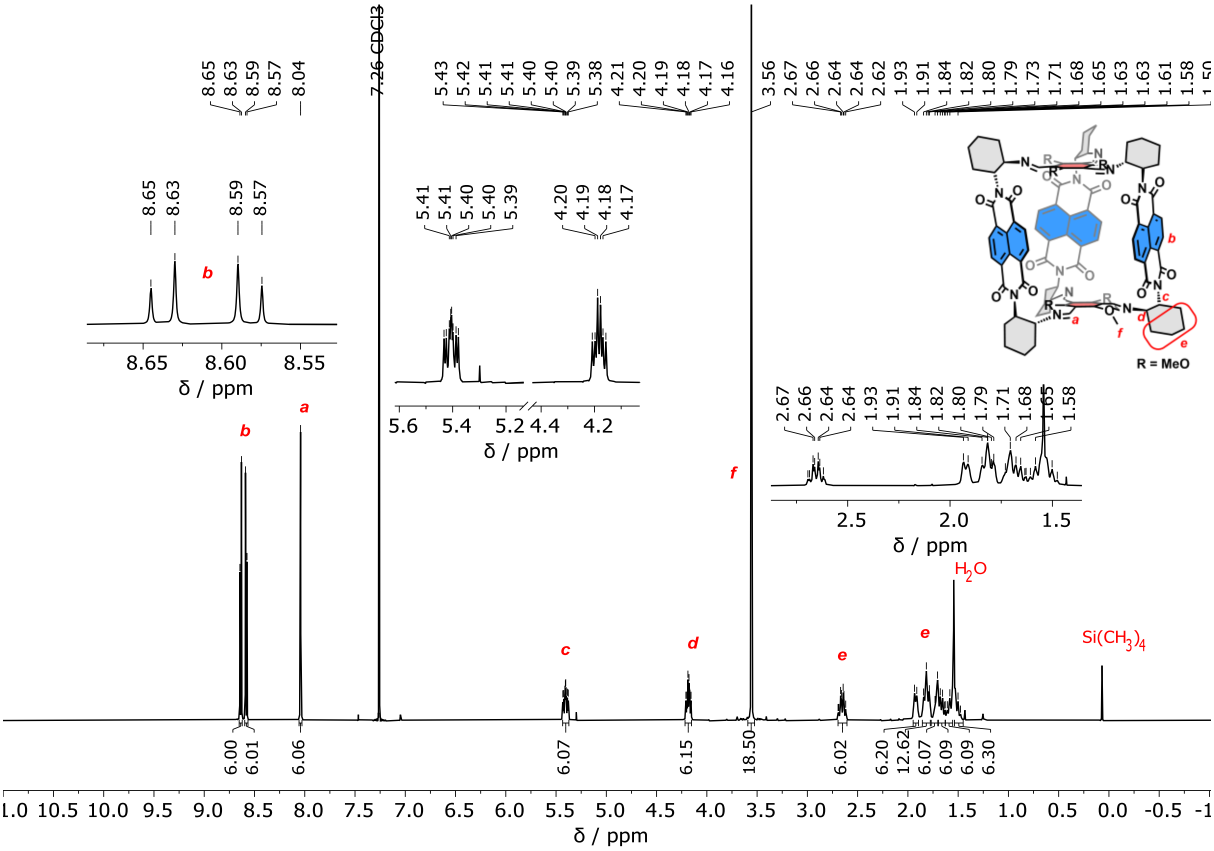

Fig. S13 ^{1}H NMR (500 MHz, $CDCl_3$, 298 K) spectrum of cage **1b**.

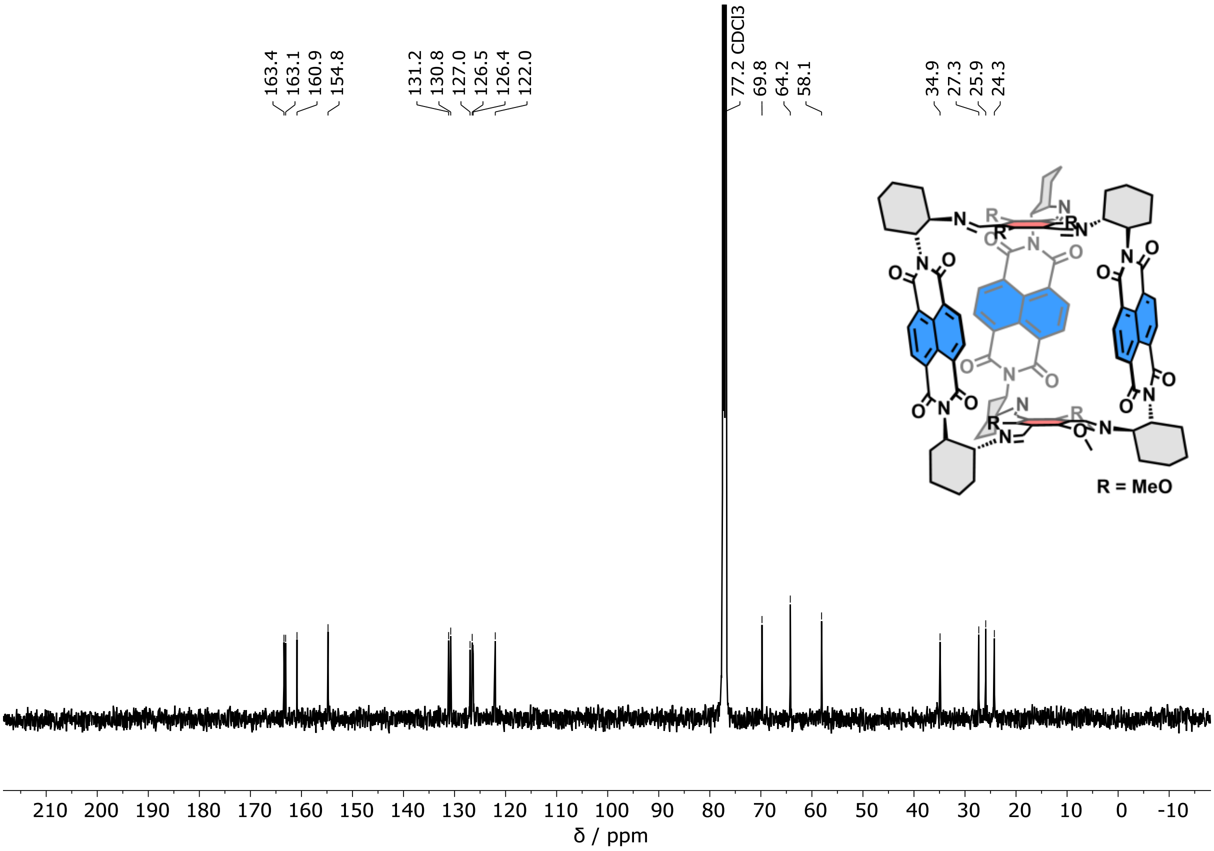

Fig. S14 $^{13}C\{^{1}H\}$ NMR (126 MHz, $CDCl_3$, 298 K) spectrum of cage **1b**.

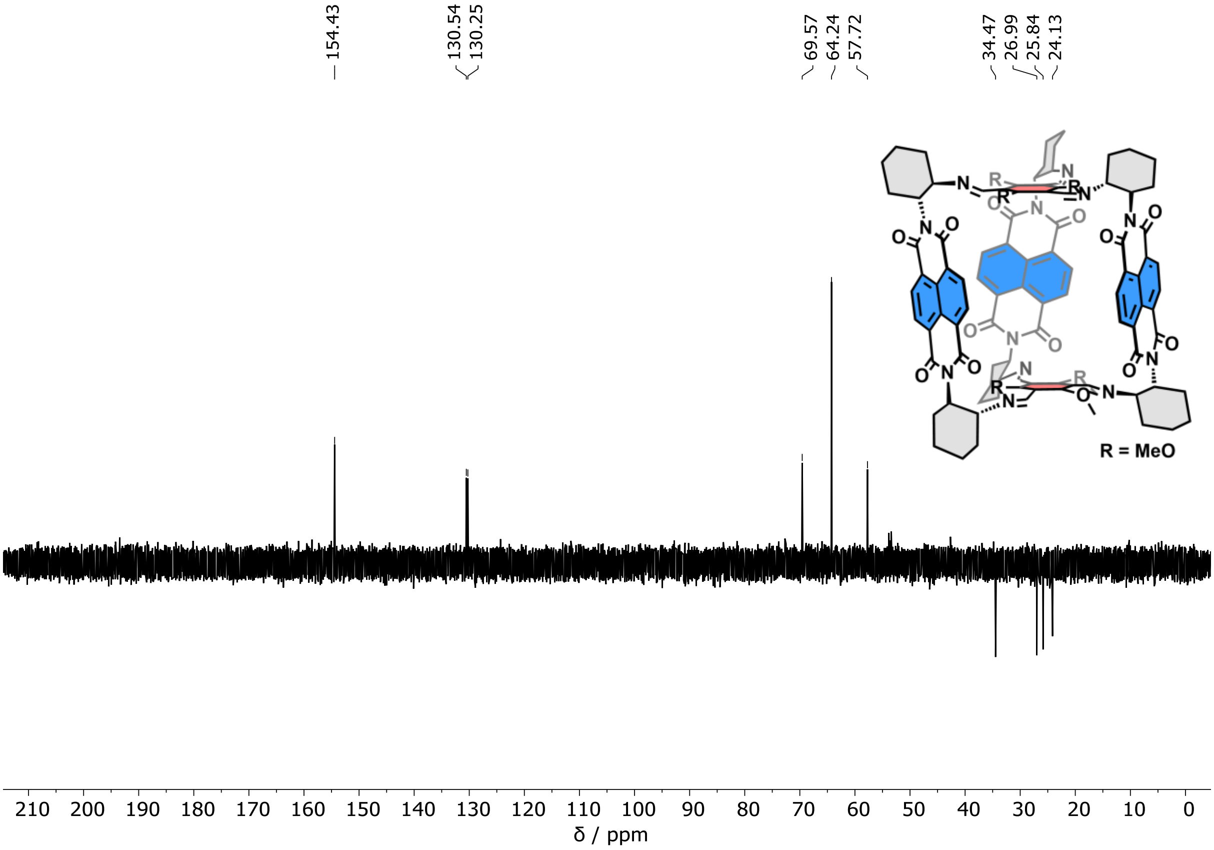

Fig. S15 ^{13}C-DEPT135-NMR (126 MHz, $CDCl_3$, 298 K) spectrum of cage **1b**.

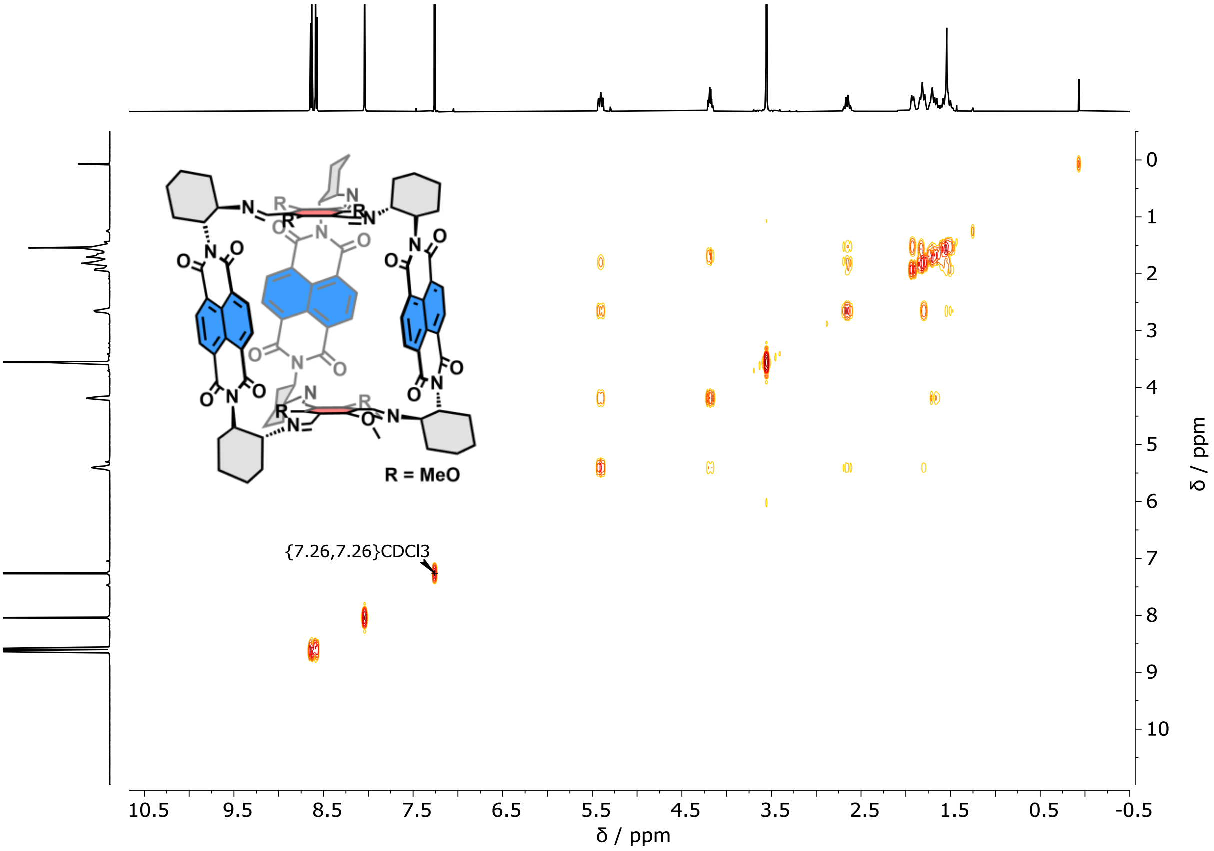

Fig. S16 ^{1}H-^{1}H COSY NMR (500 MHz, $CDCl_3$, 298 K) spectrum of cage **1b**.

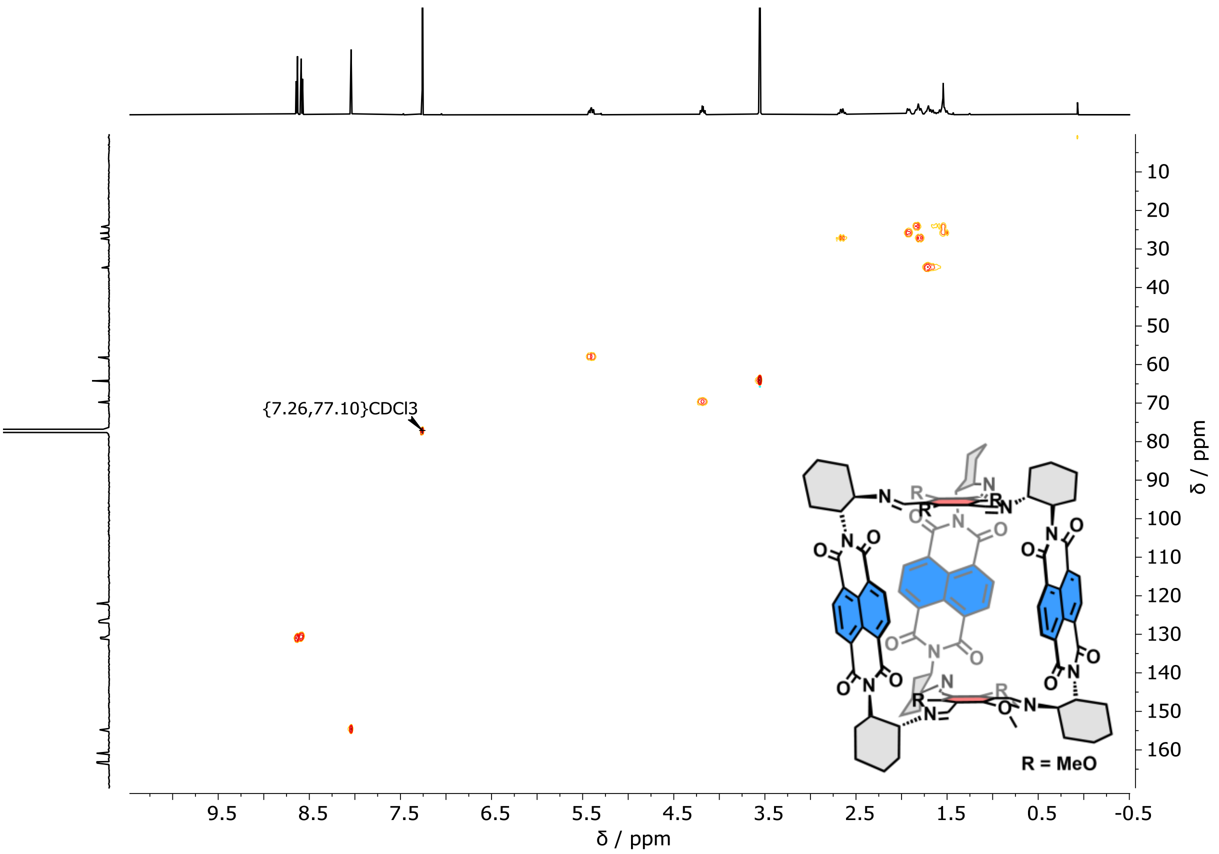

Fig. S17 ^{1}H-^{13}C HSQC NMR (126 MHz, $CDCl_3$, 298 K) spectrum of cage **1b**.

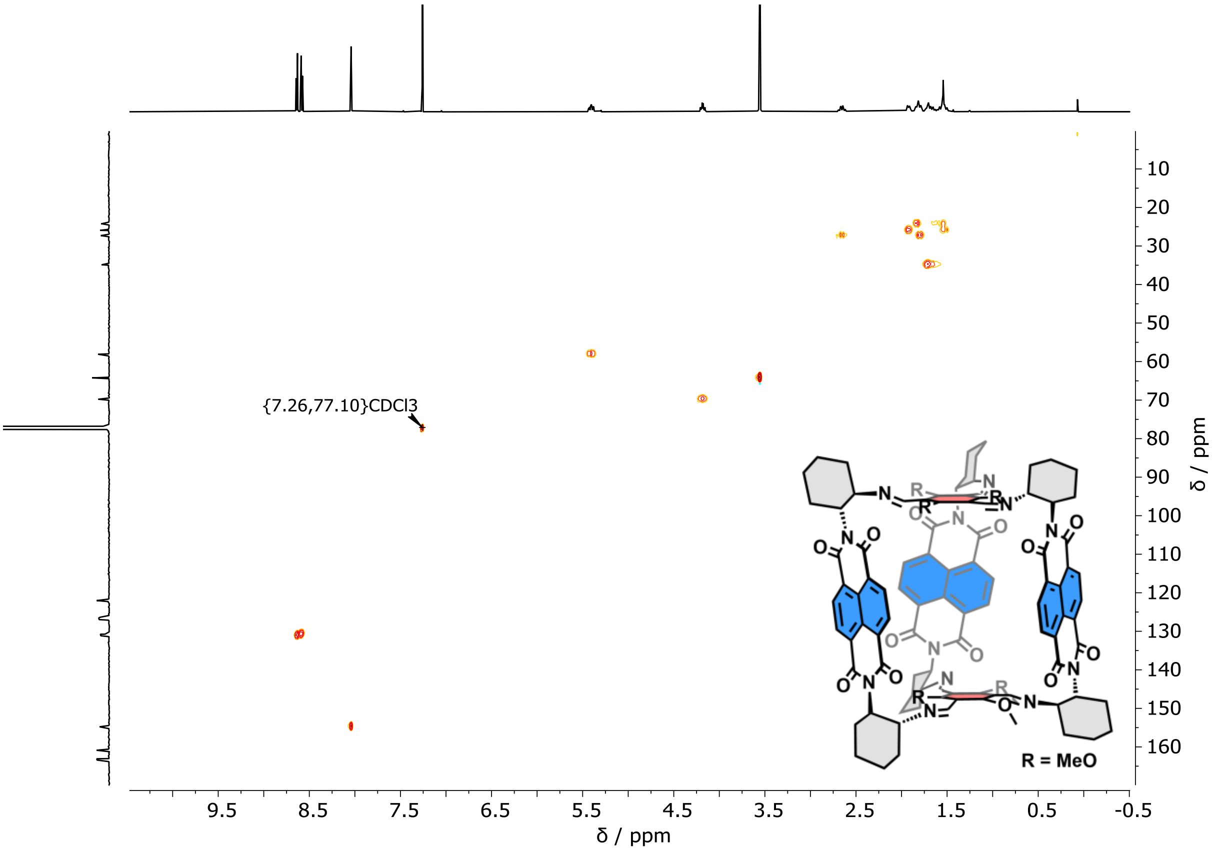

Fig. S18 ^{1}H-^{13}C HMBC NMR (126 MHz, $CDCl_3$, 298 K) spectrum of cage **1b**.

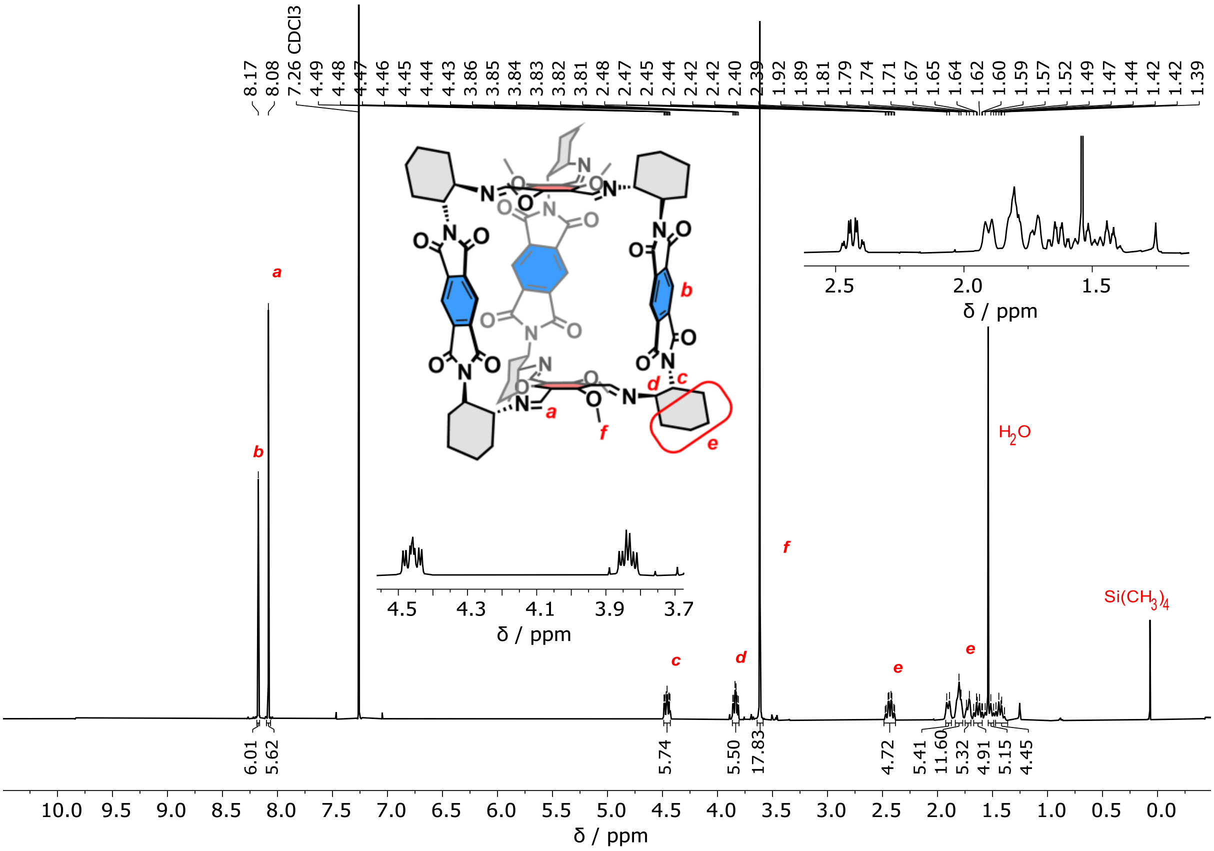

Fig. S19 ^{1}H NMR (500 MHz, $CDCl_3$, 298 K) spectrum of cage **2b**.

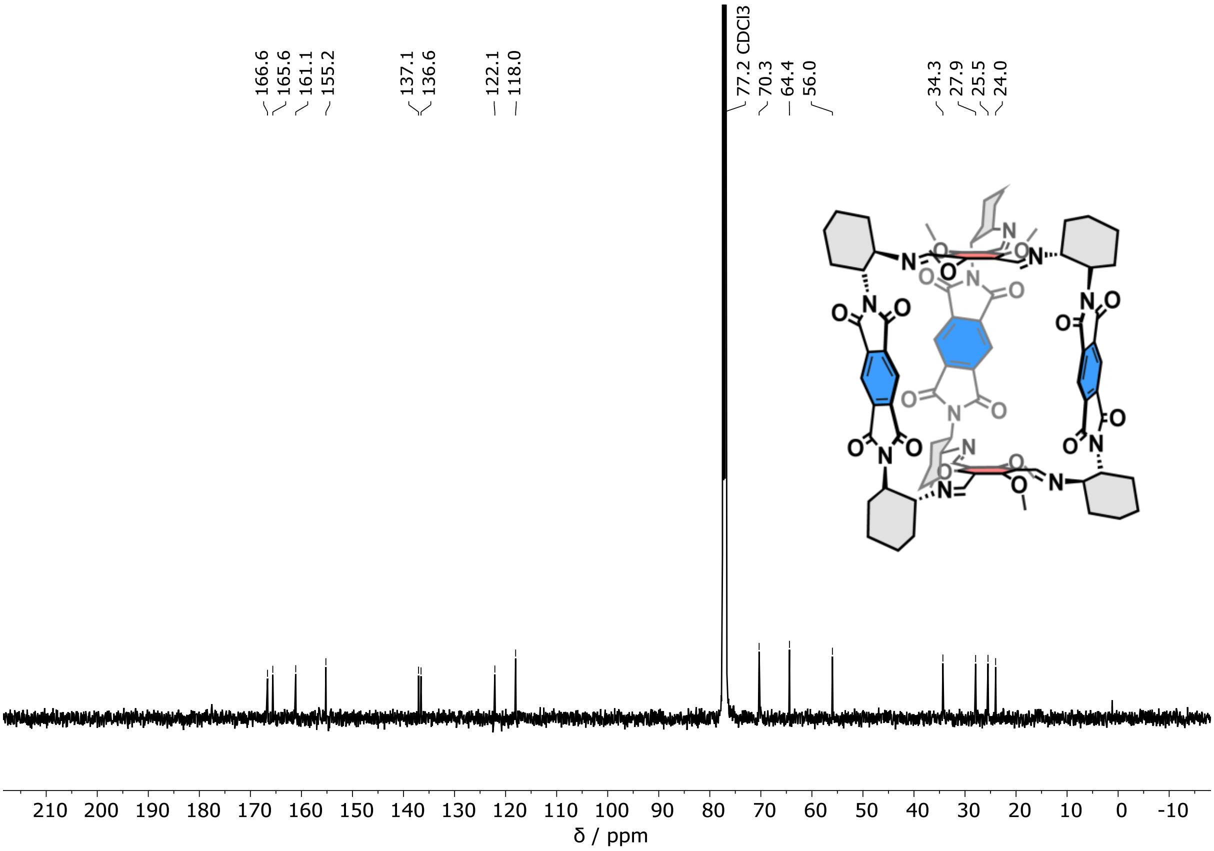

Fig. S20 $^{13}C\{^1H\}$ NMR (126 MHz, $CDCl_3$, 298 K) spectrum of cage **2b**.

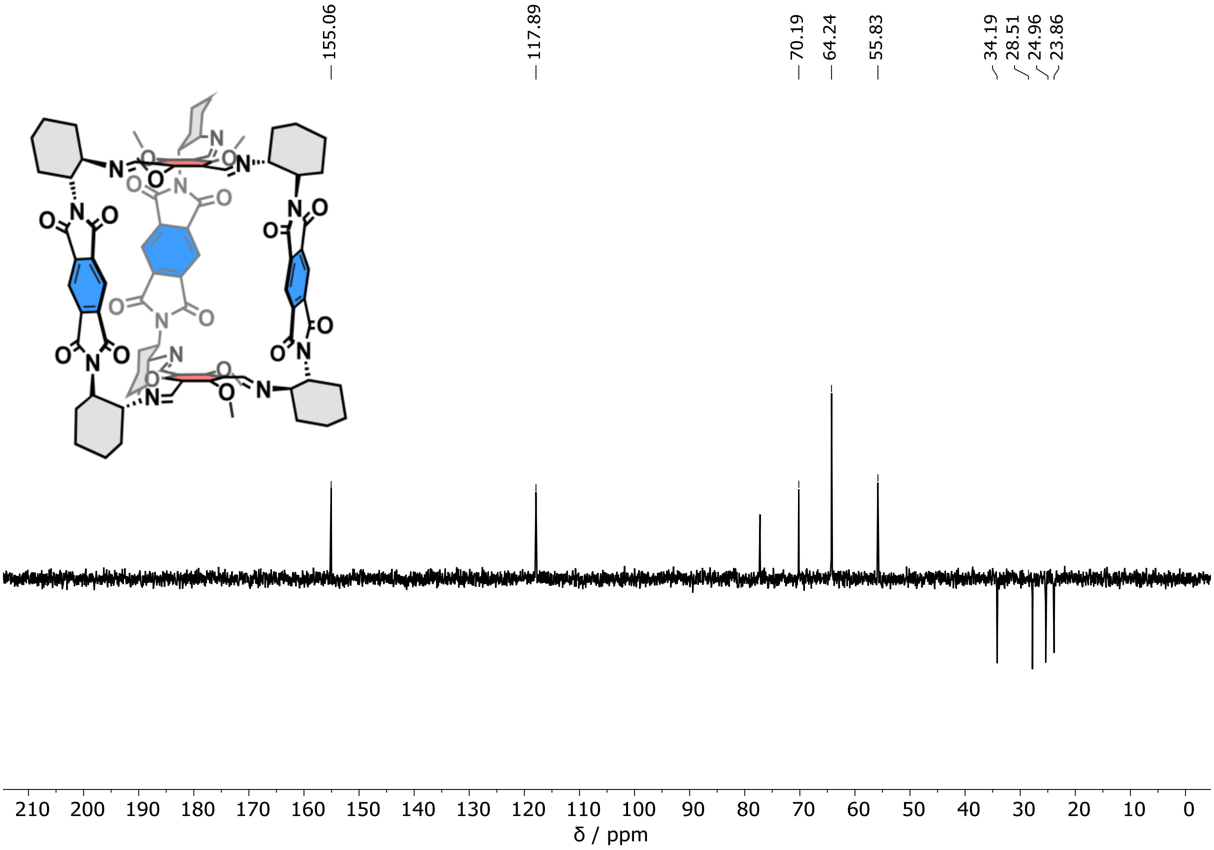

Fig. S21 ^{13}C-DEPT135-NMR (500 MHz, $CDCl_3$, 298 K) spectrum of cage **2b**.

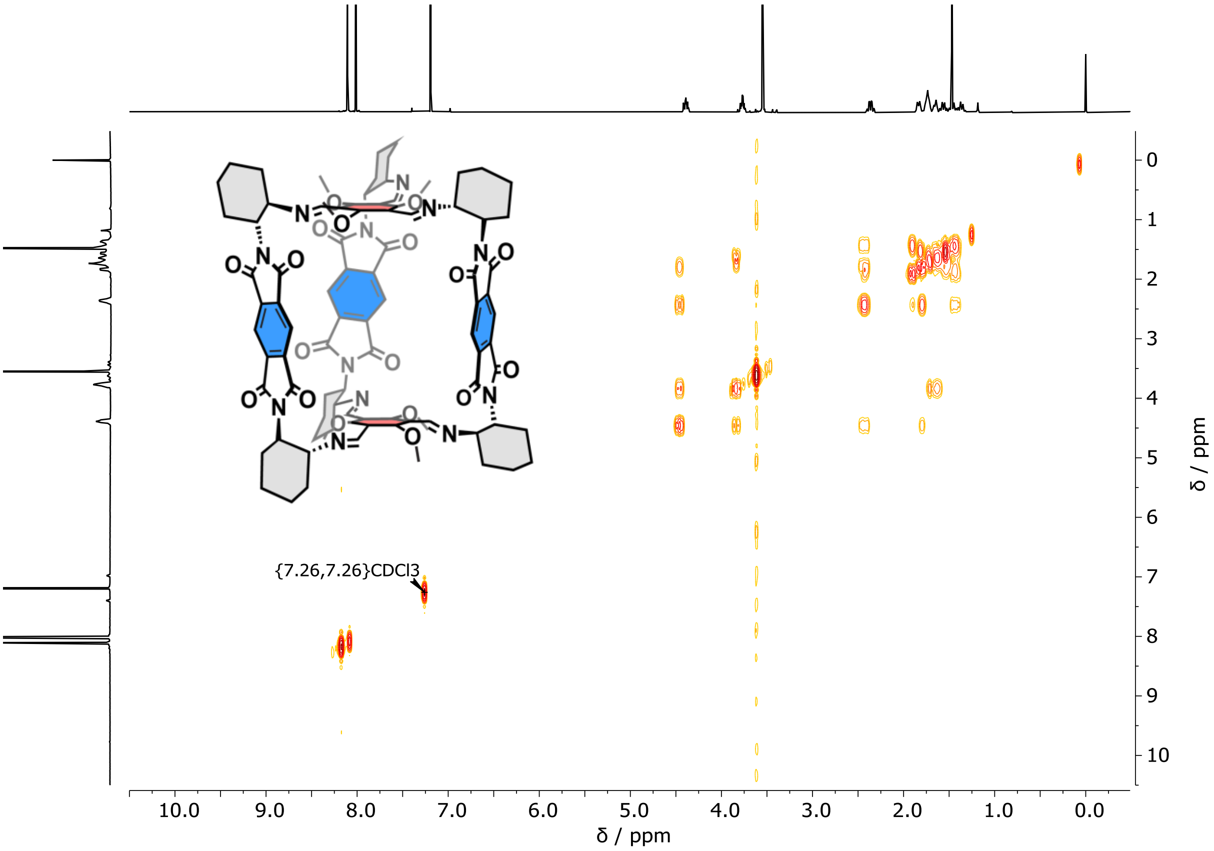

Fig. S22 ^{1}H-^{1}H COSY NMR (500 MHz, $CDCl_3$, 298 K) spectrum of cage **2b**.

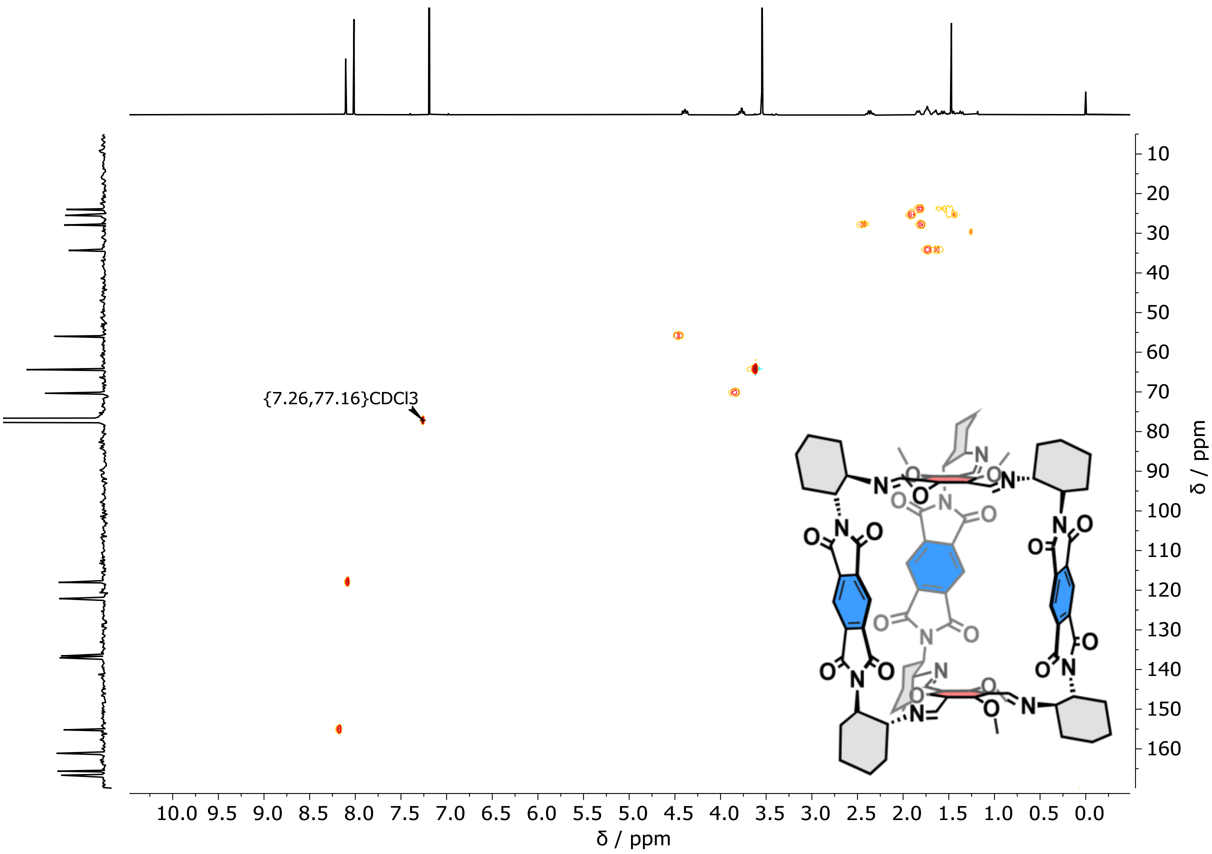

Fig. S23 ^{1}H-^{13}C HSQC NMR (500 MHz, $CDCl_3$, 298 K) spectrum of cage **2b**.

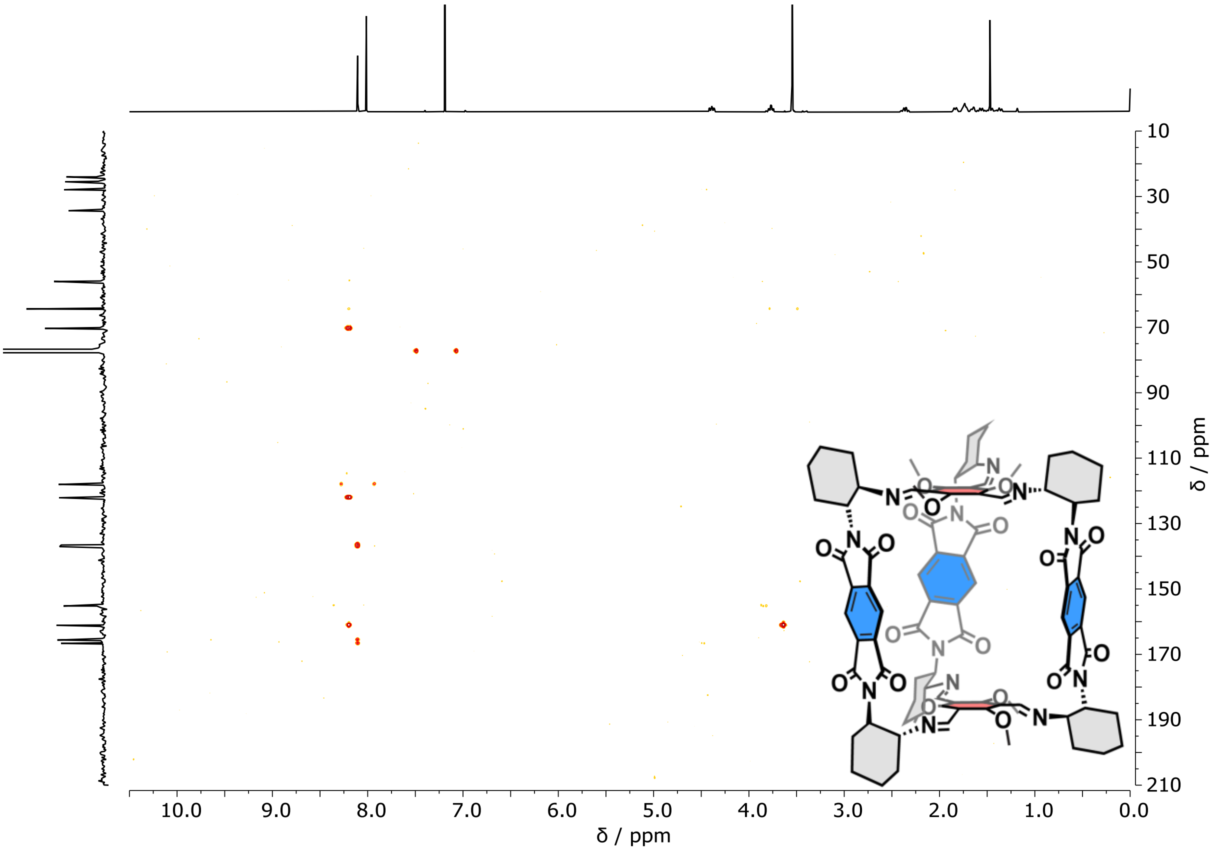

Fig. S24 ^{1}H-^{13}C HMBC NMR (500 MHz, $CDCl_3$, 298 K) spectrum of cage **2b**.

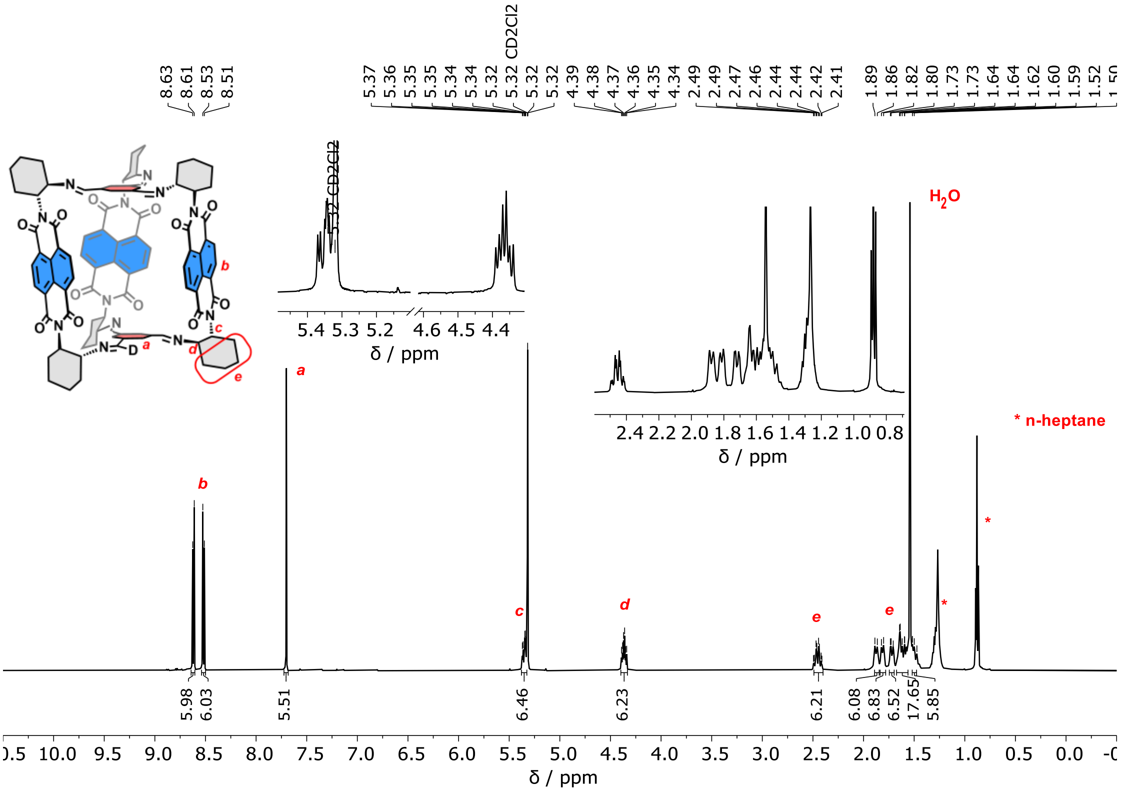

Fig. S25 ^1H NMR (500 MHz, CD_2Cl_2, 298 K) spectrum of cage **1a**-d_6. Residual solvent signals correspond to heptane and are assigned with an asterisk.

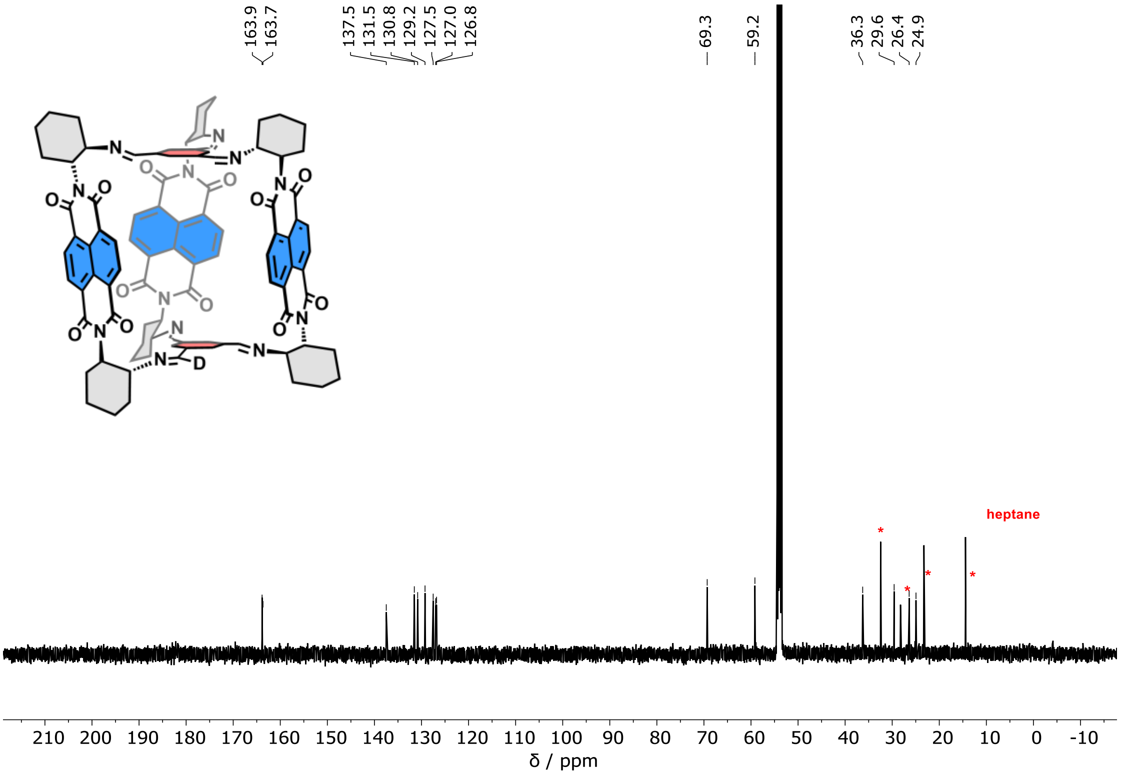

Fig. S26 $^{13}C\{^1H\}$ NMR NMR (126 MHz, CD_2Cl_2, 298 K) spectrum of cage **1a**-d_6. Residual solvent signals correspond to heptane and are assigned with an asterisk.

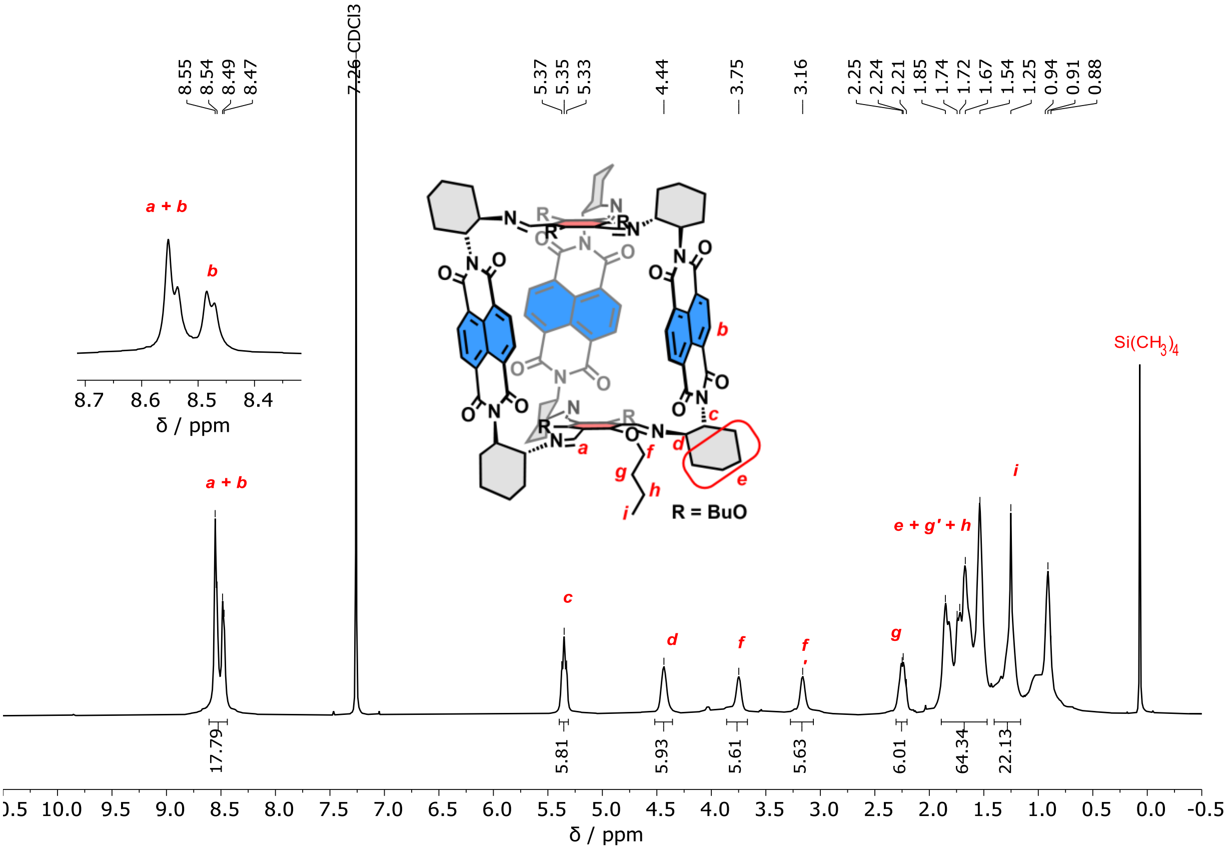

Fig. S27 ^{1}H NMR (500 MHz, $CDCl_3$, 298 K) spectrum of cage **1d**.

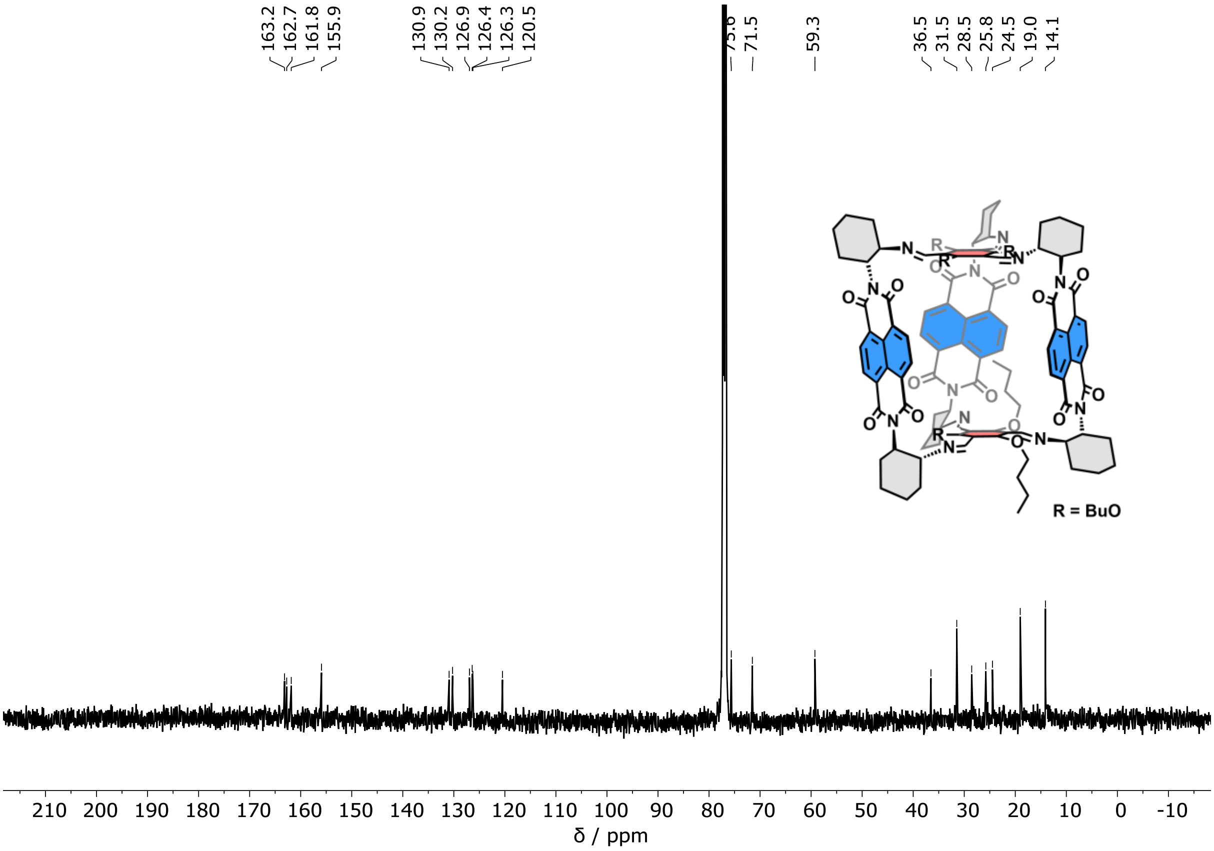

Fig. S28 $^{13}C\{^1H\}$ NMR (126 MHz, $CDCl_3$, 298 K) spectrum of cage **1d**.

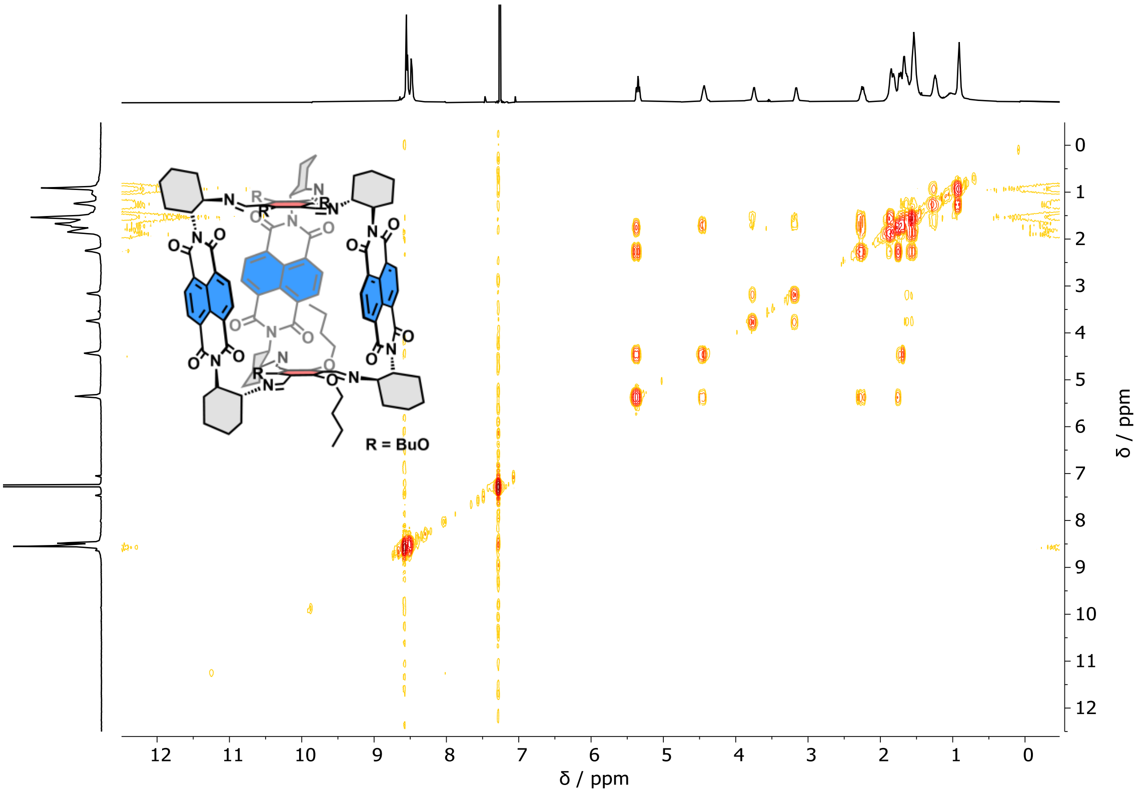

Fig. S29 ^{1}H-^{1}H COSY NMR (500 MHz, $CDCl_3$, 298 K) spectrum of cage **1d**.

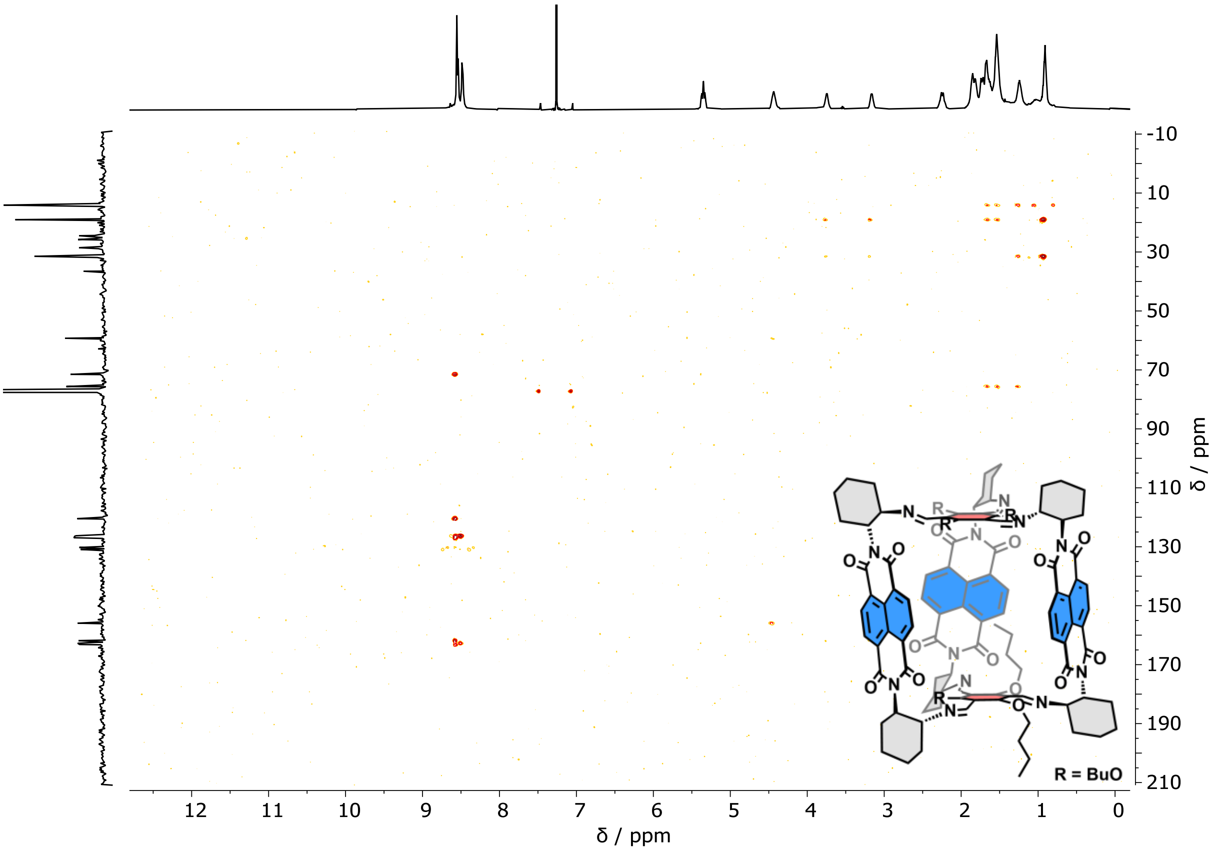

Fig. S30 ^{1}H-^{13}C HMBC NMR (500 MHz, $CDCl_3$, 298 K) spectrum of cage **1d**.

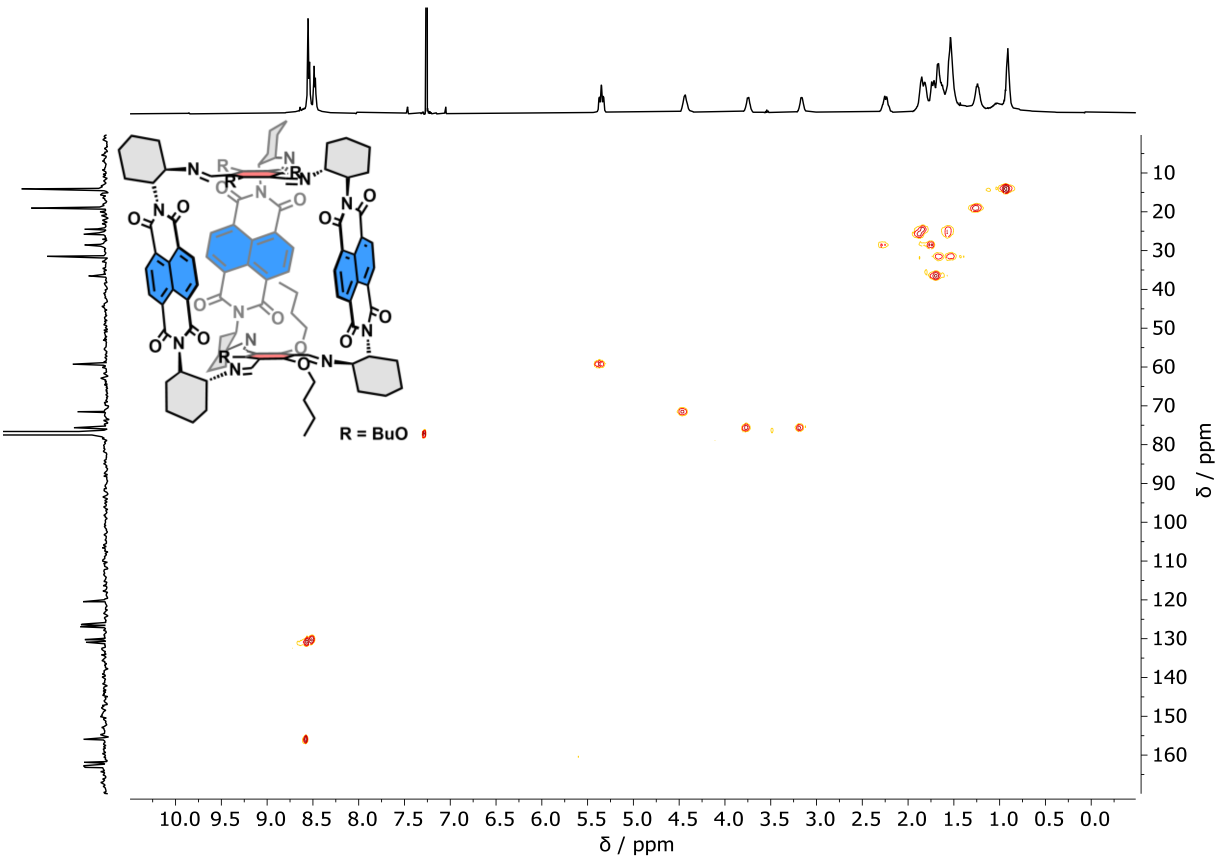

Fig. S31 ^{1}H-^{13}C HSQC NMR (500 MHz, $CDCl_3$, 298 K) spectrum of cage **1d**.

1.2. High-resolution mass spectrometry analysis

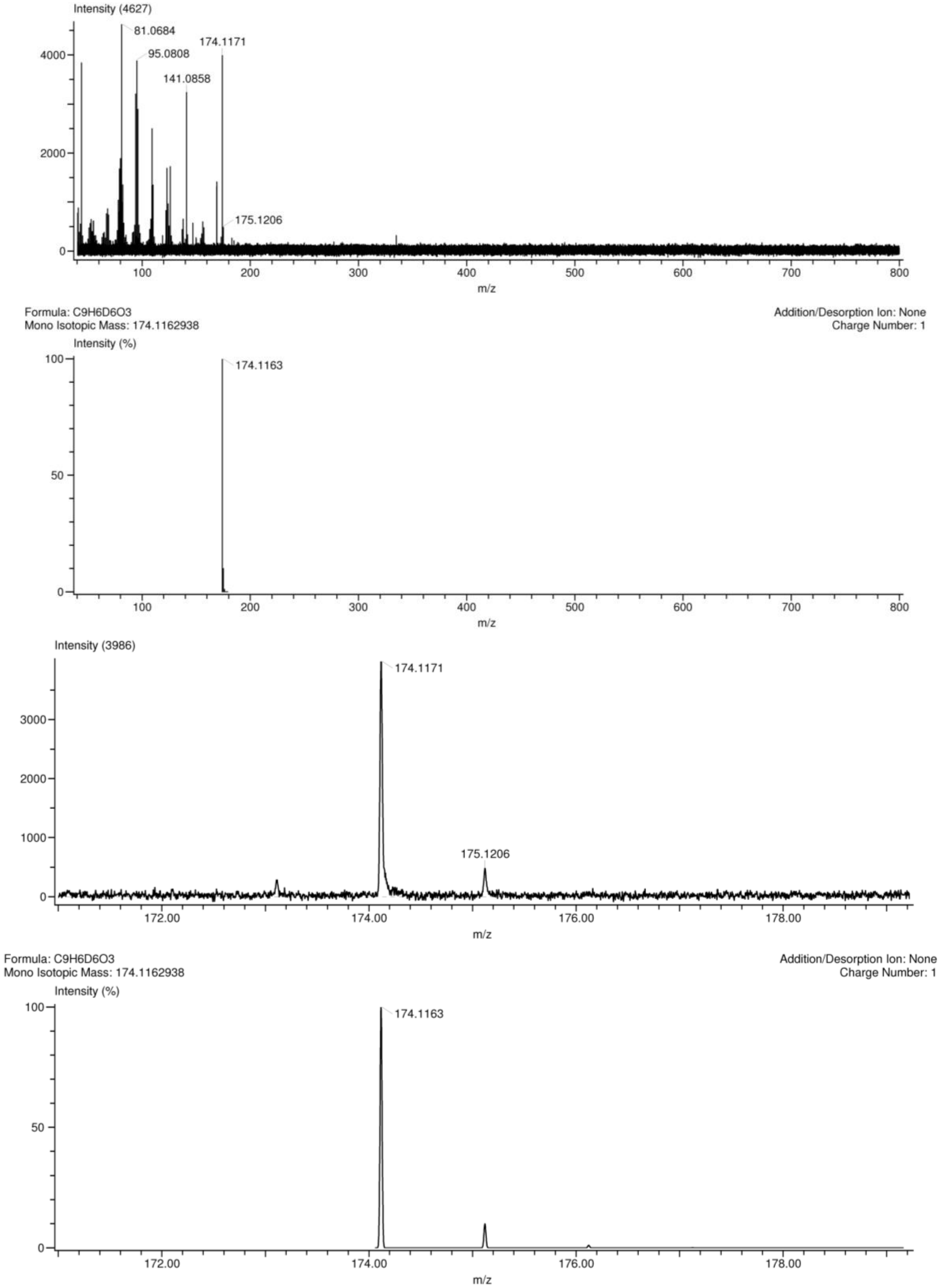

Fig. S32 Full high resolution ESI MS spectrum of 1,3,5-tris(hydroxy($^2H_6$)methyl)benzene (top). As well as the observed and calculated isotopic pattern for $[C_9H_6D_6O_3]^+$ (bottom).

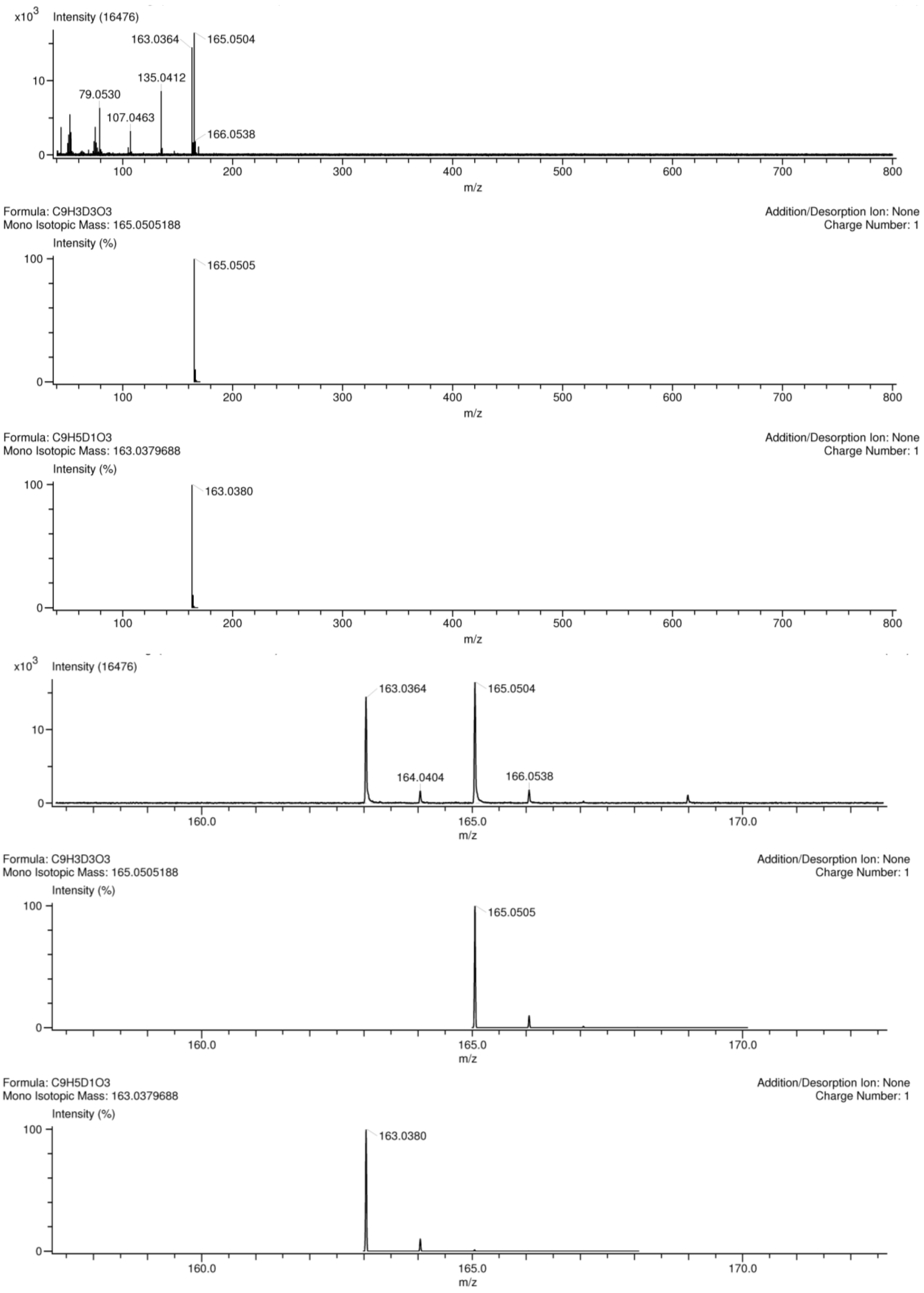

Fig. S33 Full high resolution ESI MS spectrum of 1,3,5-tri($^{2}H_3$)formylbenzene (top). As well as the observed and calculated isotopic pattern for $[C_9H_3D_3O_3]^+$ and $[C_9H_5D_1O_3]^+$ (bottom).

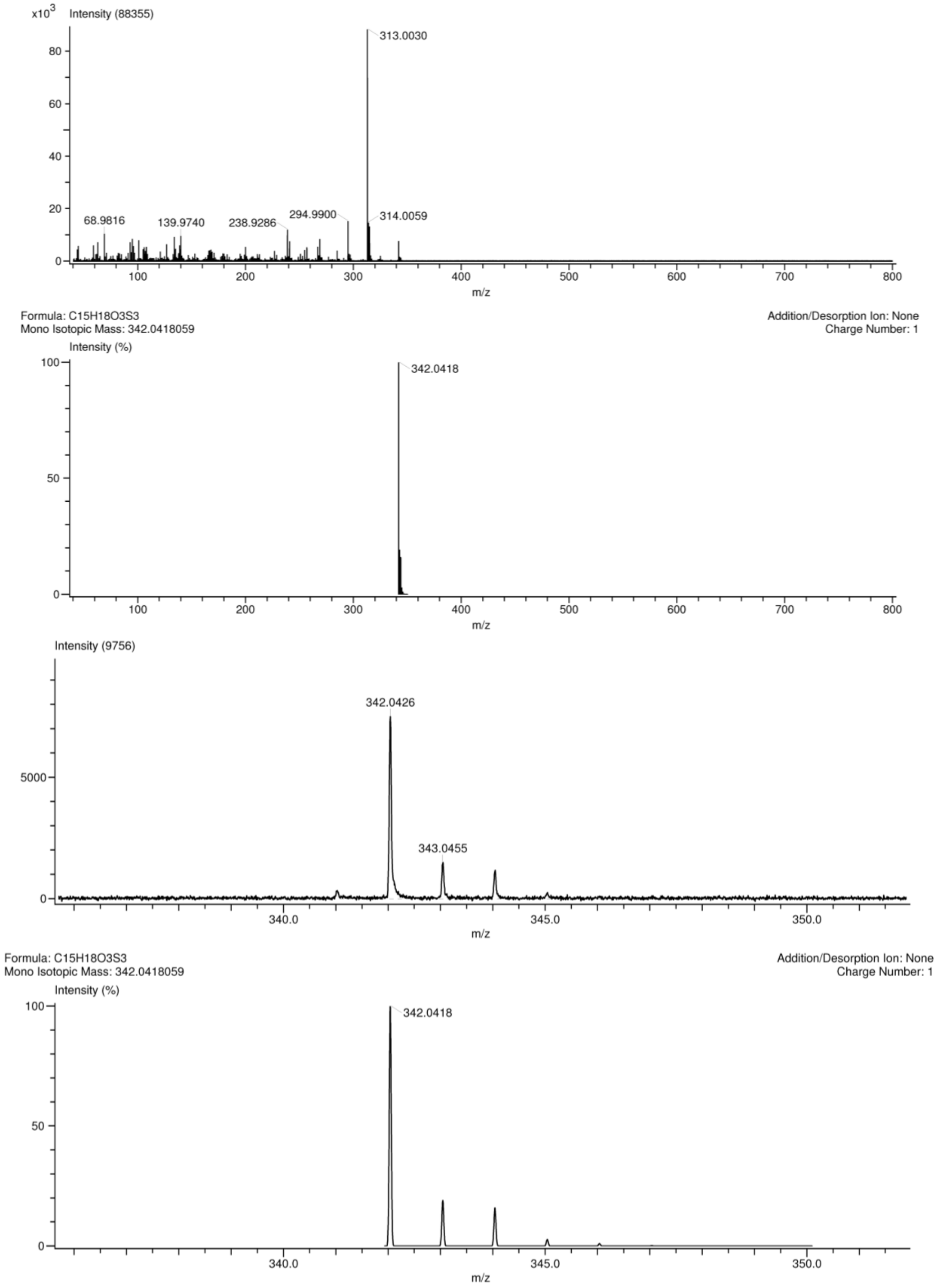

Fig. S34 Full high resolution ESI MS spectrum of 2,4,6-triethylthioether-1,3,5-tricarbaldehyde **3c** (top). As well as the observed and calculated isotopic pattern for $[C_{15}H_{18}O_3S_3]^+$ (bottom).

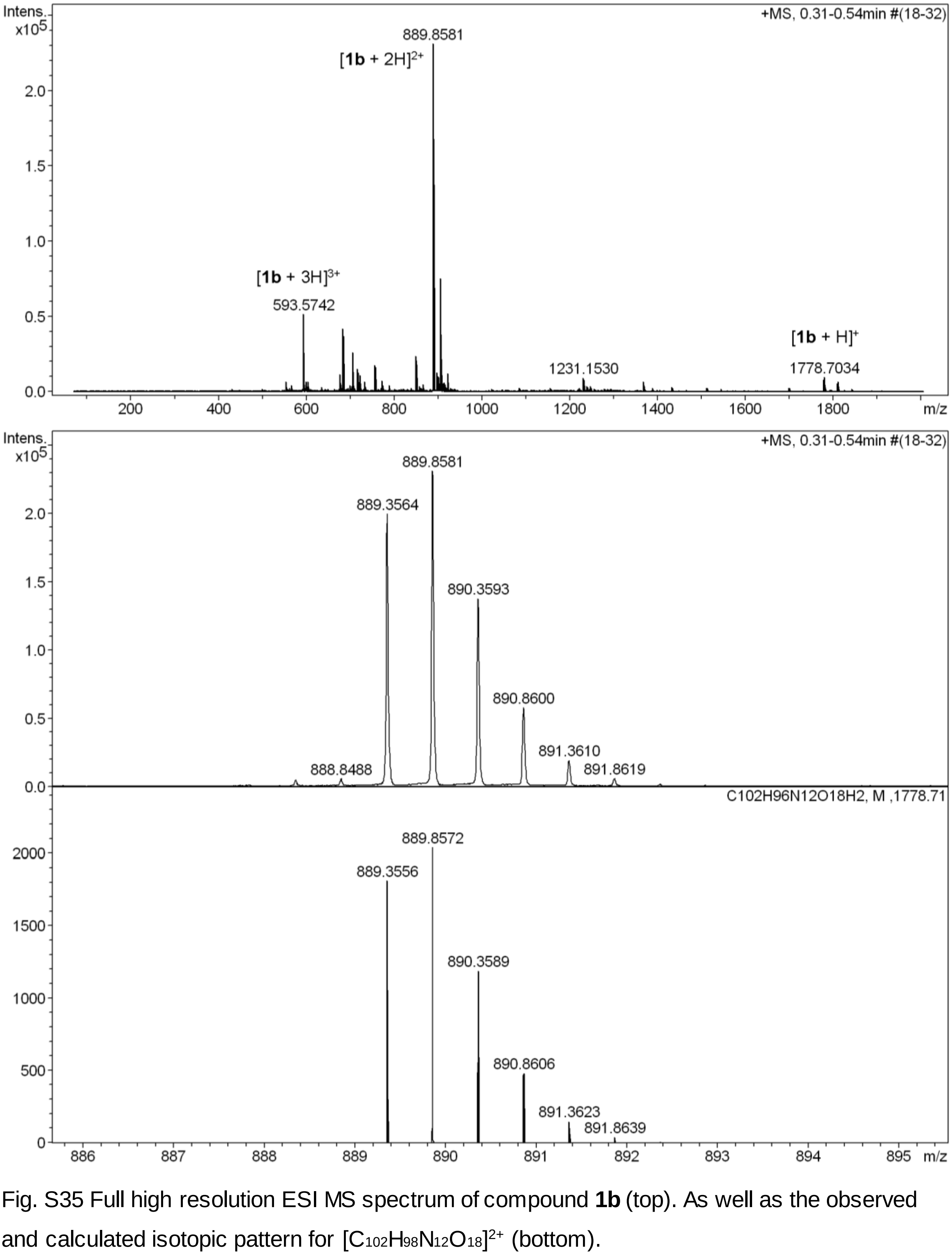

Fig. S35 Full high resolution ESI MS spectrum of compound **1b** (top). As well as the observed and calculated isotopic pattern for $[C_{102}H_{98}N_{12}O_{18}]^{2+}$ (bottom).

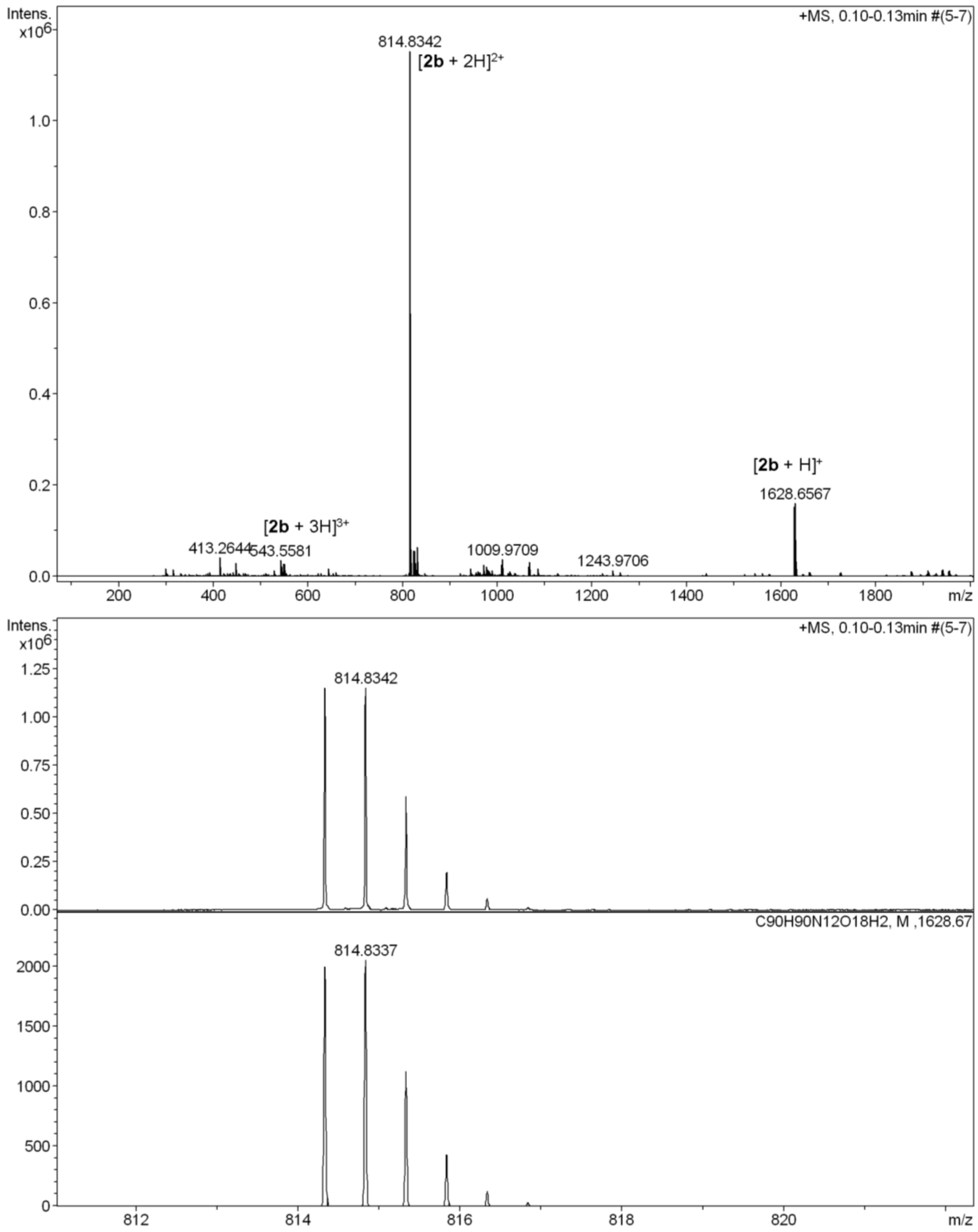

Fig. S36 Full high resolution ESI MS spectrum of compound **2b** (top). As well as the observed and calculated isotopic pattern for $[C_{90}H_{92}N_{12}O_{18}]^{2+}$ (bottom).

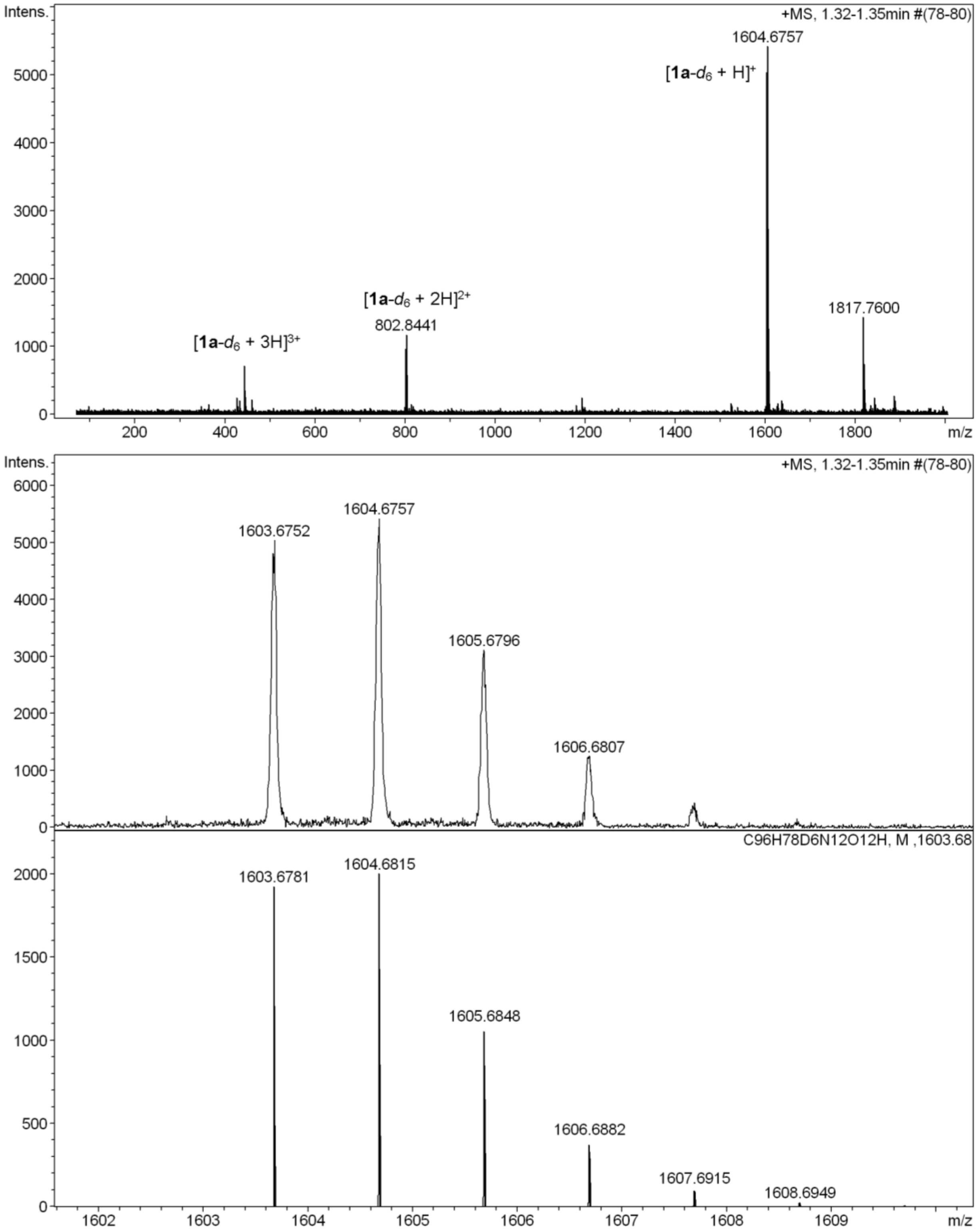

Fig. S37 Full high resolution ESI MS spectrum of compound **1a**-*d*6 (top). As well as the observed and calculated isotopic pattern for $[C_{96}H_{80}D_6N_{12}O_{18}]^{2+}$ (bottom).

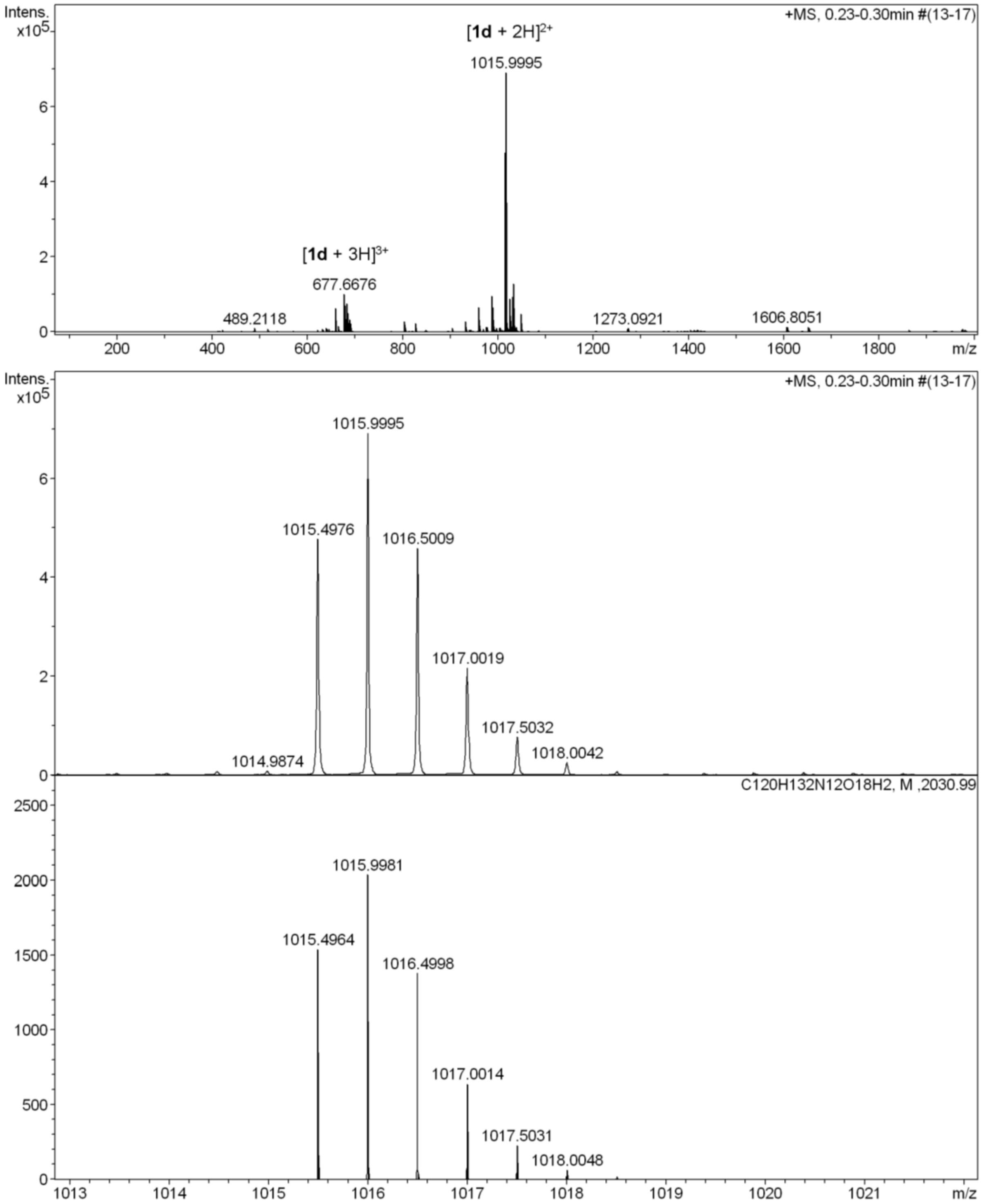

Fig. S38 Full high resolution ESI MS spectrum of compound **1d** (top). As well as the observed and calculated isotopic pattern for $[C_{120}H_{134}N_{12}O_{18}]^{2+}$ (bottom).

1.3. FT-IR analysis

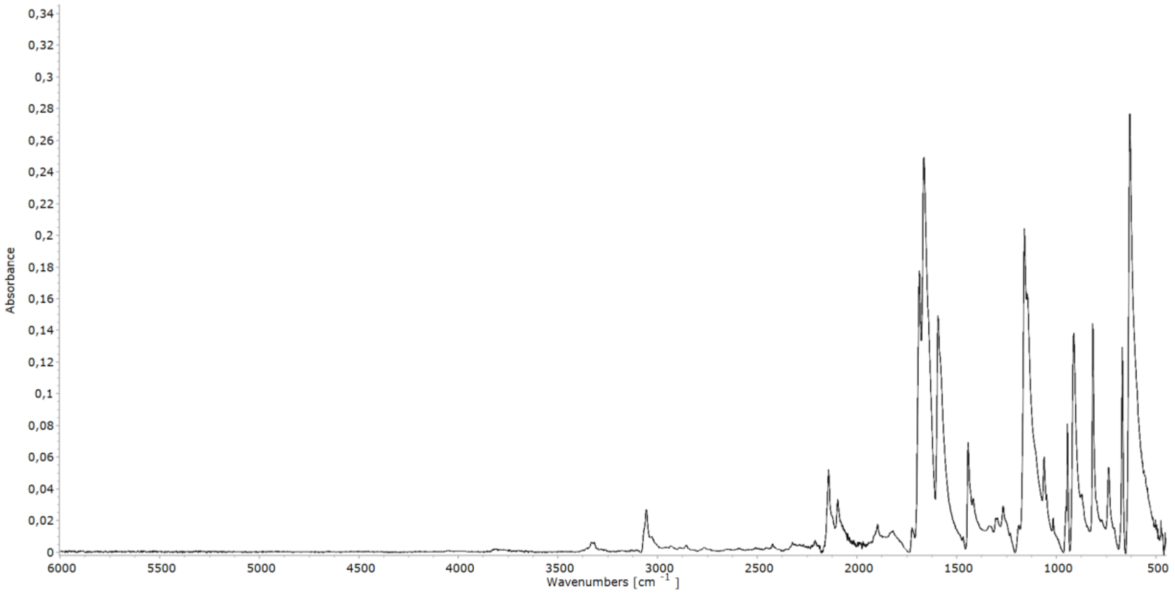

Fig. S39 FT-IR spectrum of compound **3a**-*d*6.

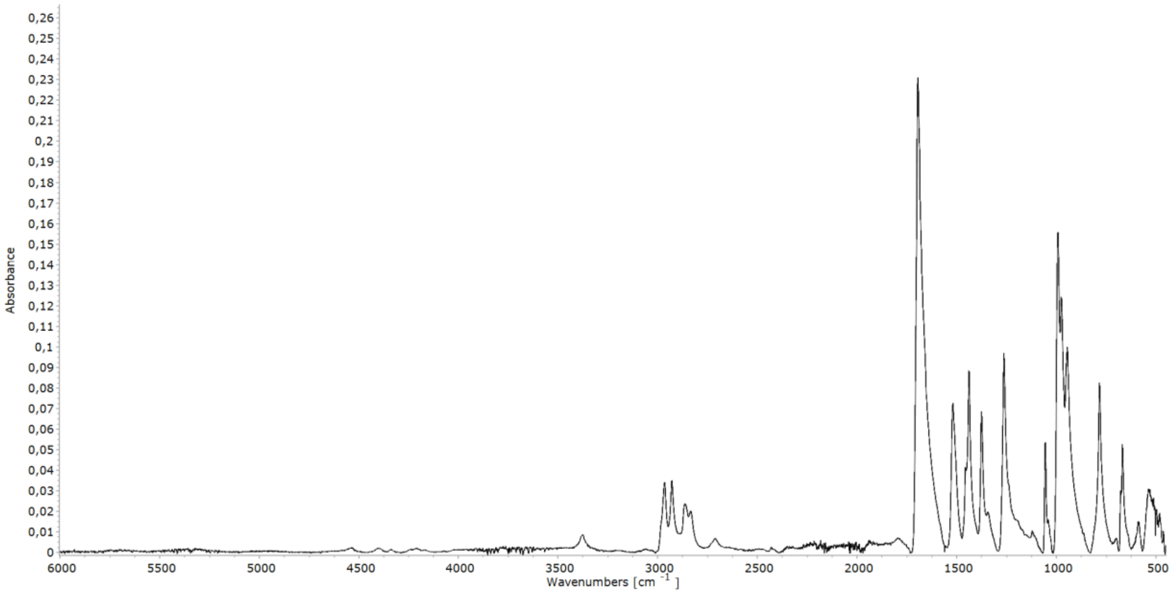

Fig. S40 FT-IR spectrum of compound **3c**.

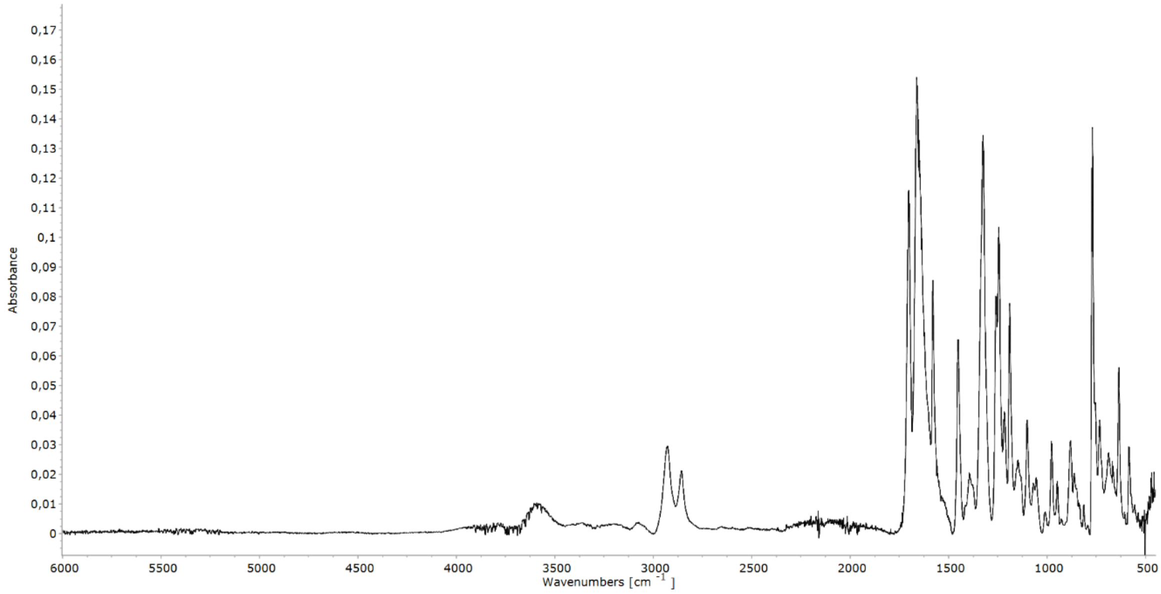

Fig. S41 FT-IR spectrum of compound **1a**.

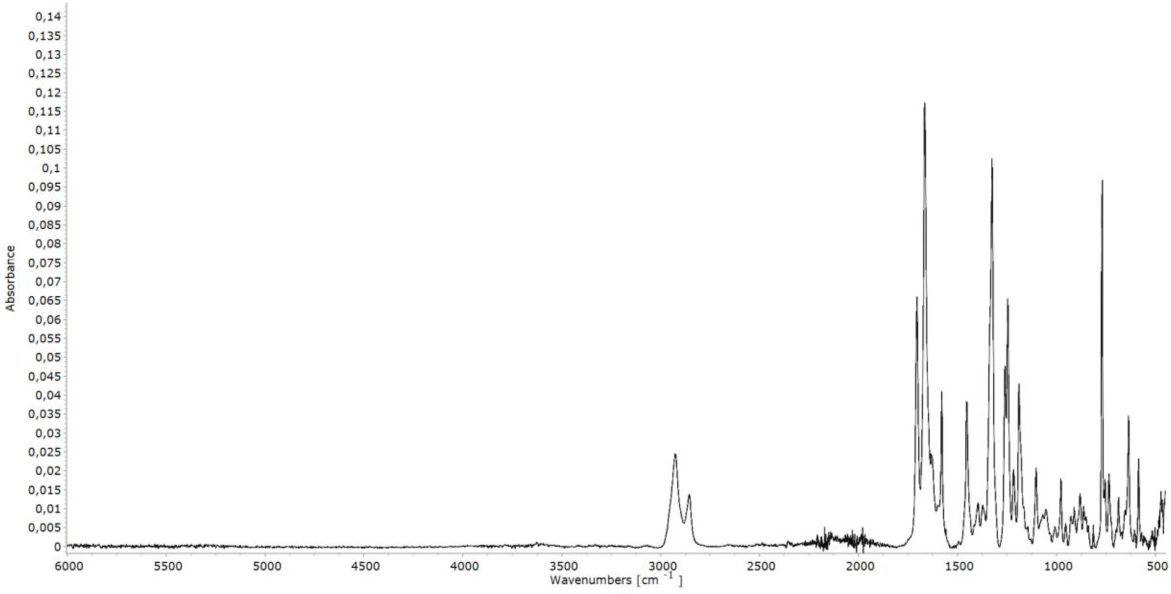

Fig. S42 FT-IR spectrum of compound **1a**-d_6.

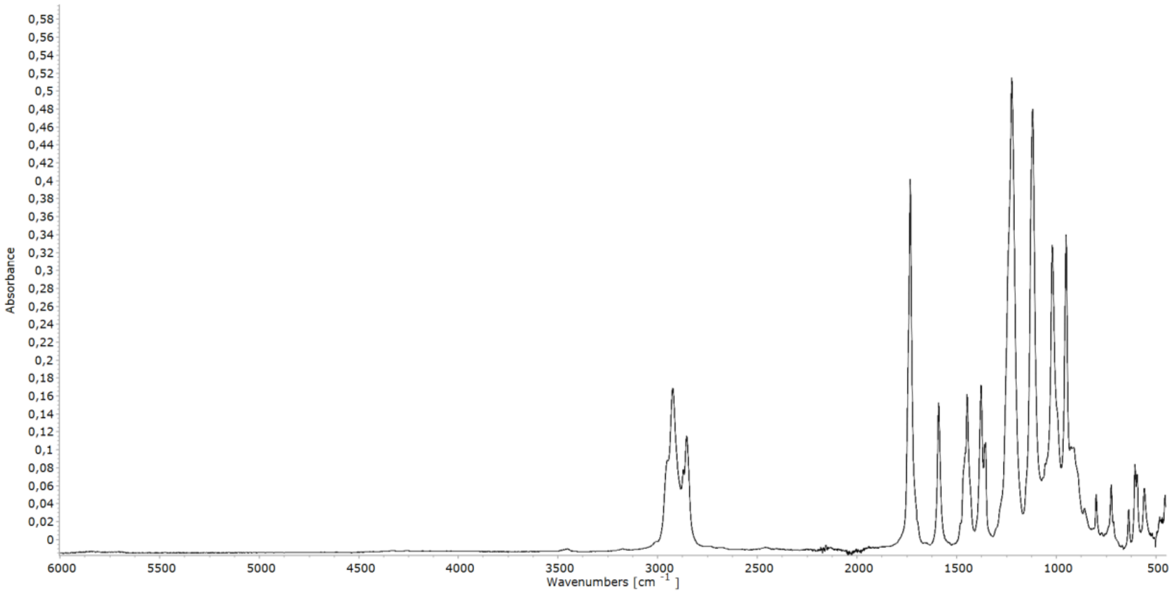

Fig. S43 FT-IR spectrum of compound **3d**.

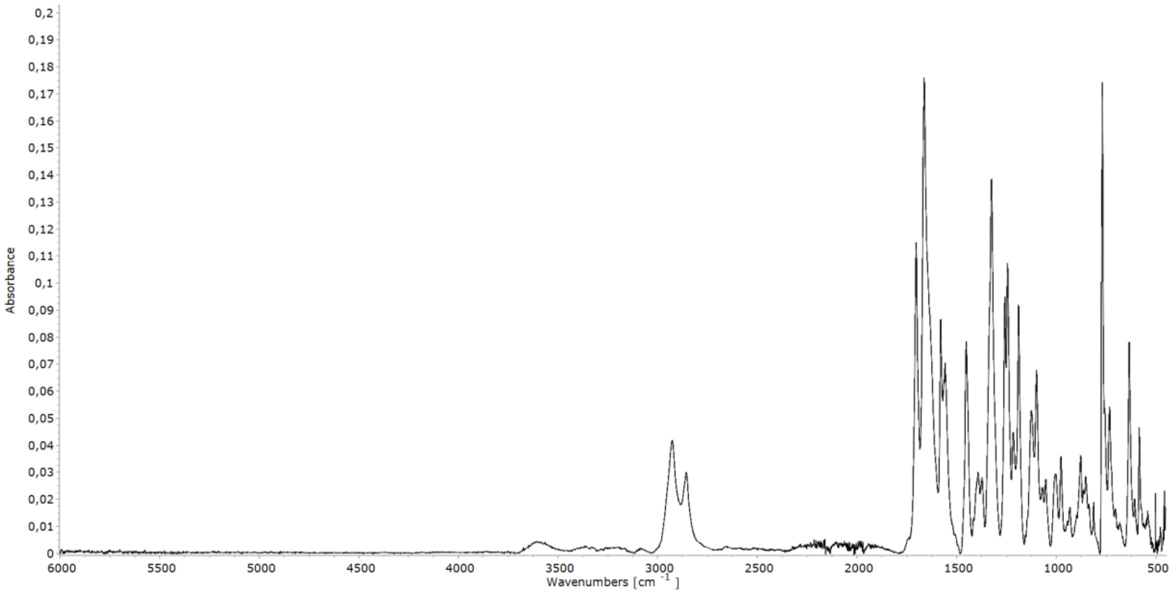

Fig. S44 FT-IR spectrum of compound **1b**.

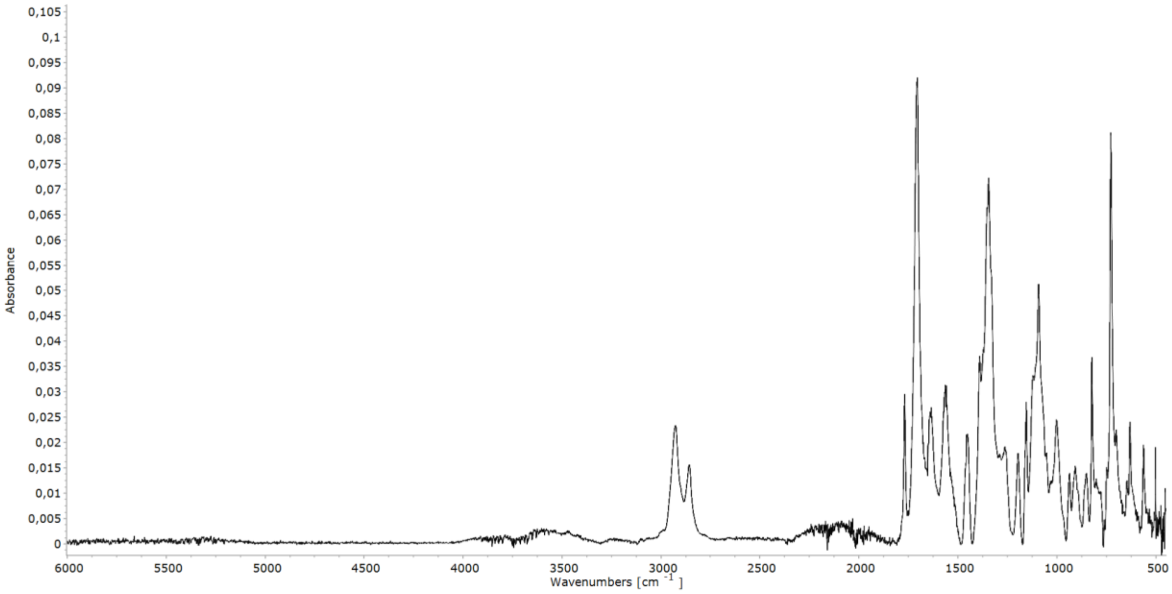

Fig. S45 FT-IR spectrum of compound **2b**.

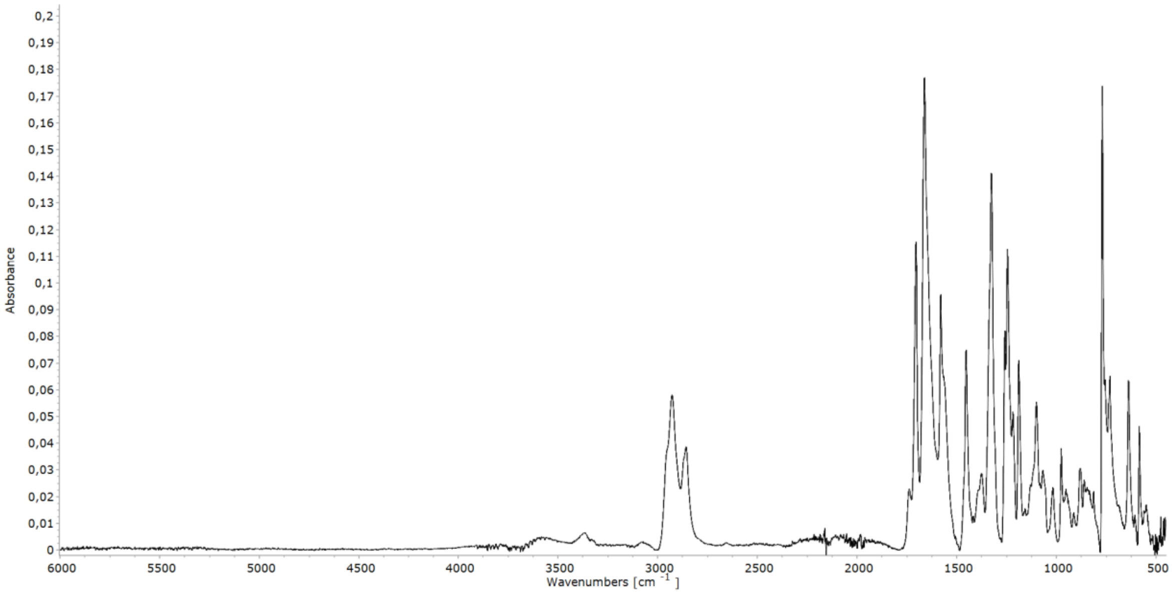

Fig. S46 FT-IR spectrum of compound **1d**.

2. Crystallographic details

General remarks

Single colorless plate-shaped crystals of **1a** were grown by slow vapor diffusion of acetonitrile into a nitrobenzene solution of the cage over the course of several days. Single yellow block-shaped crystals of **1d** were grown by slow vapour diffusion of methanol into a THF solution of the cage over several days. A suitable crystal was selected and mounted on a mylar loop in perfluoroether oil on a STOE StadiVari diffractometer (STOE & Cie GmbH, Darmstadt, Germany) equipped with a Pilatus300K detector and a Metaljet D2 source (Ga K_α radiation). The crystal was kept at a steady T = 150 K during data collection. The structures were solved with the ShelXT[10] structure solution program using the Intrinsic Phasing or dual method and by using Olex[11] as the graphical interface. The model was refined with version 2018/3 of ShelXL[12] using full-matrix least-squares minimization on F^2.

Table S2. Crystal data and structure refinement for **1b** and **1d**.

Structure	**1b**	**1d**
Identification code	2145026	2373256
Empirical formula	$C_{102}H_{96}N_{12}O_{18}$ · 2.5 C_2H_3N · 3.5 C_8H_{10}	$C_{120}H_{132}N_{12}O_{18}$
Formula weight	2231.57	2030.37
Temperature/K	150	150
Crystal system	Monoclinic	Orthorhombic
Space group	$P2_1$	$P2_12_12_1$
a / Å	16.1500(8)	16.2359(3)
b / Å	24.0088(7)	25.8812(4)
c / Å	16.3444(8)	30.5609(7)
α / °	90	90
β / °	99.061(4)	90
γ / °	90	90
Volume / $Å^3$	6258.3(5)	12841.8(4)
Z	2	4
ρ_{calc} / g cm^{-3}	1.184	1.050
μ / mm^{-1}	0.363	0.369
F(000)	2366	4320.0
Crystal size/mm^3	0.25 × 0.157 × 005	0.14 × 0.13 × 0.12
Radiation	Ga K_α (λ = 1.34143)	Ga K_α (λ = 1.34143)
2Θ range for data collection / °	2.382–56.832	3.892–100
Index ranges	$-20 \le h \le 19$, $-20 \le k \le 14$, $-20 \le l \le 19$	$-18 \le h \le 18$, $-15 \le k \le 29$, $-34 \le l \le 34$
Reflections collected	65808	100062
Independent reflections	16900 [R_{int} = 0.1177, R_{sigma} = 0.1153]	19941 [R_{int} = 0.0625, R_{sigma} = 0.0327]
Data/restraints/parameters	16900/84/1286	19941/11/1354
Goodness-of-fit on F^2	0.977	1.204
Final R indexes [I>=2σ (I)]	R_1 = 0.1439, wR_2 = 0.2542	$R1$ = 0.1053, $wR2$ = 0.2900
Final R indexes [all data]	R_1 = 0.1027, wR_2 = 0.2775	$R1$ = 0.1476, $wR2$ = 0.3378
Largest diff. peak/hole / e $Å^{-3}$	0.764/–0.426	0.93/–0.28
Flack parameter	0.2(4)	0.4(4)

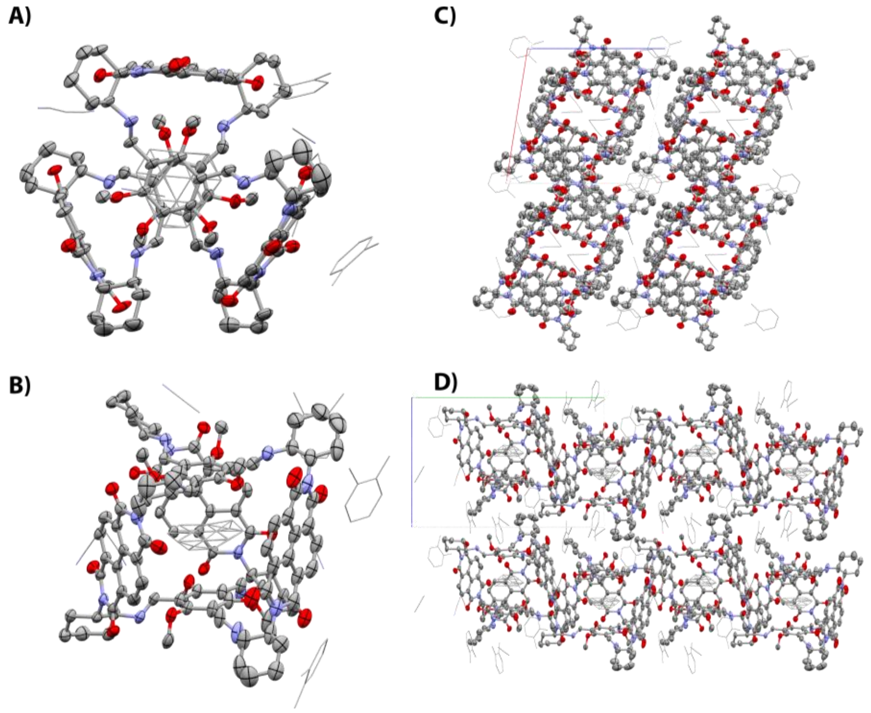

Fig. S47 The crystal structure of **1b** with displacement parameters visualized at 50% probability obtained at 150 K from the side (A) and top view (B) as well as the crystal packing along the *a*-axis (C) and *b*-axis (D). Color code: C, grey; O, red; N, blue. Nitrobenzene molecules are shown in wireframe style and hydrogens are omitted for clarity.

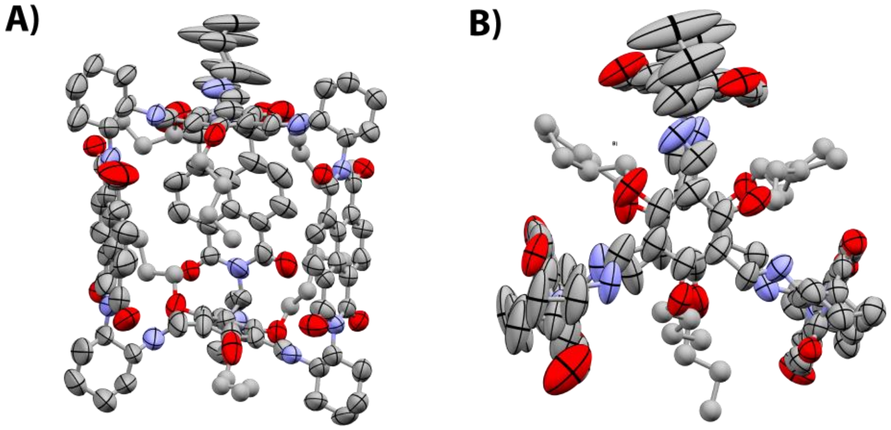

Fig. S48 The crystal structure of **1d** with displacement parameters visualized at 50% probability obtained at 150 K from the side (A) and top view (B). Color code: C, grey; O, red; N, blue. Butly chains are shown in ball-stick style and hydrogens are omitted for clarity.

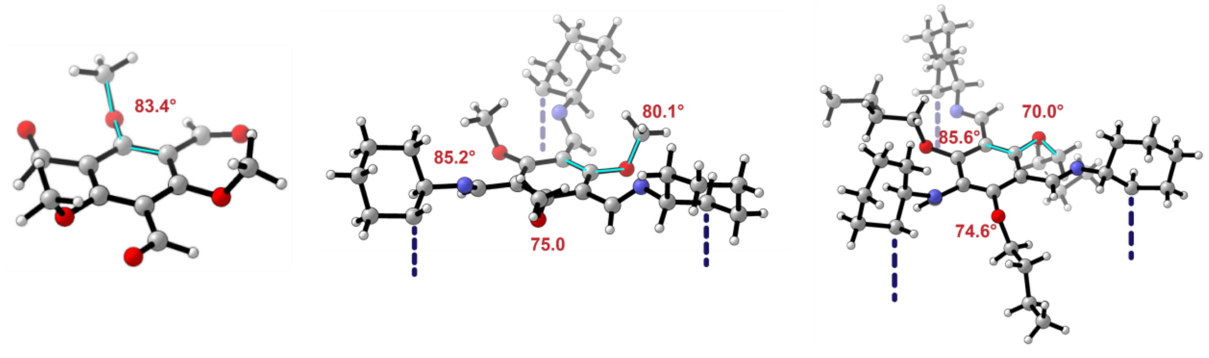

3b **1b** **1d**

Fig. S49 Representation of the structure of **3b** (geometry obtained on B3LYP/6-31G(d) level of theory), and truncated arene bridges of **1b** and **1d** (geometry obtained from SC-XRD experiments). The connection towards the cage is indicated with blue dashed lines. The H_3**C**–**O**–**C**$_{Ar}$–**C**$_{Ar}$ torsion angles are given in red. Selected bonds to the torsion angle are highlighted in blue.

3. Exchange experiments

3.1 Isotopic purity of deuterated compounds

The cage **1a** as well as **1a**-d_6 show an isotopic pattern in MALDI-MS that does not correspond to simulated distributions. The isotopic purity of cage **1a**-d_6 is given by the bridge **3a**-d_3. For the synthesis of the **3a** precursor 1,3,5-tris(hydroxy($^2H_6$)methyl)benzene, we used in our experiments 98% isotopically pure $LiAl^2H_4$ which can result in a statistical mixture of non-deuterated to six-fold deuterated triol. After oxidation to trialdehyde **3a**-d_3, ^{1}H NMR spectroscopy shows a residual resonance for protonated aldehydic protos that corresponds to approximately 99% isotopic purity. However, HR-FD-MS indicates a resonance intensity ratio of 1:1 between **3a**-d_1 and **3a**-d_3. MS seems to overestimate the content of non-deutorated species in **3a**-d_3 and consequently also **1a**-d_6.

3.2. MALDI MS experiments

General remarks

The samples were prepared by mixing stock solutions of the corresponding reagents in $CDCl_3$. The corresponding cage (0.3 mM, 0.1 mL), and **3a** (0.3 mM, 0.1 mL), or 2,2,2-trifluoroacetic acid (0.1 mM, 0.1 mL) were mixed in an NMR tube and topped up with additional $CDCl_3$ to 0.6 mL. If required, the reaction mixtures were heated in an oil bath without stirring. Samples were taken by spotting a capillary (3 × 10 µL onto an anthracene matrix). The measurements were performed on a Bruker RapifleX MALDI-TOF (27% laser power, negative reflector mode).

Table S3. Conditions for the scrambling experiments of **1a** and **1a**-*d*6 (see Figure S42).

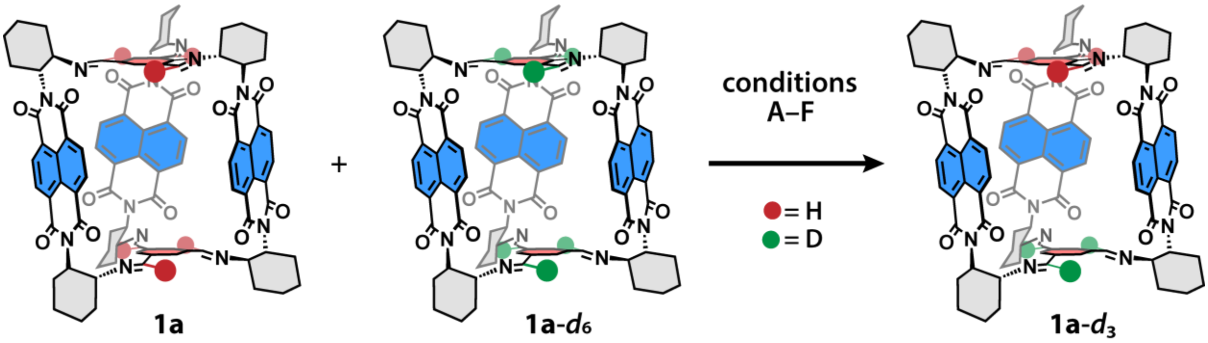

Conditions	Solvent	*T* / °C	Additive
A	Anhyd. $CHCl_3$	60	–
B	Anhyd. $CHCl_3$	25	–
C	Anhyd. $CHCl_3$	25	1 mol% TFA
D	Water sat. $CHCl_3$	60	–
E	Water sat. $CHCl_3$	25	–
F	Water sat. $CHCl_3$	25	1 mol% TFA

Table S4. Conditions for the scrambling experiments of **1a** and **3a**-*d*3 (see Figure S42).

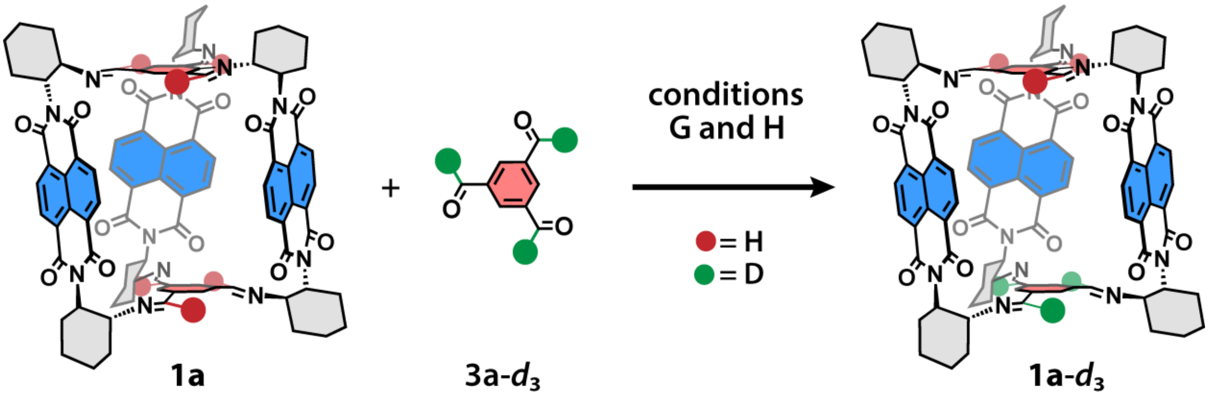

Conditions	Solvent	*T* / °C	Additive
G	Anhyd. $CHCl_3$	25	–
H	Water sat. $CHCl_3$	25	–

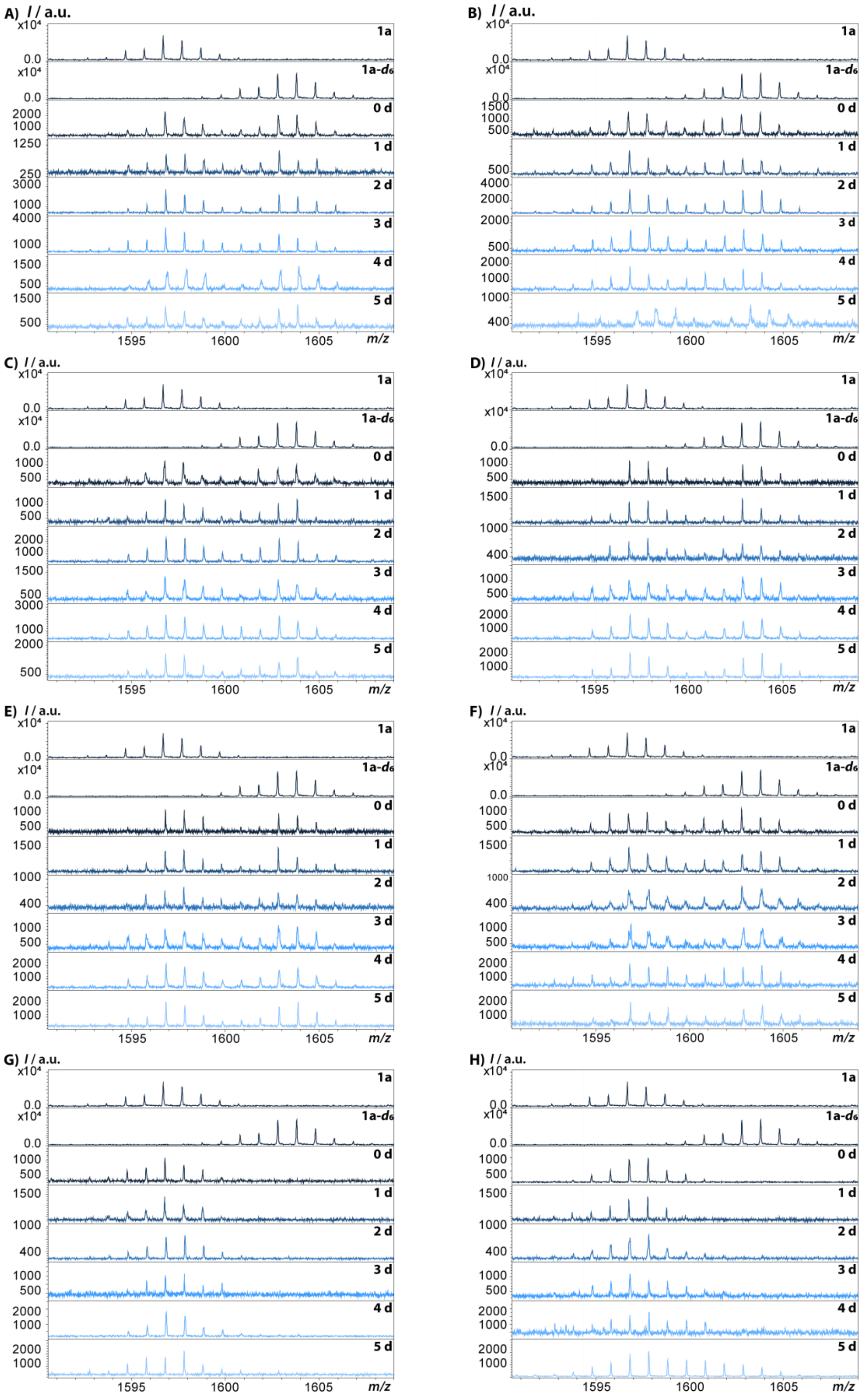

Fig. S50. Scrambling experiments of **1a**/**1a**-*d*6 (A–F) and **1a**/**3a**-*d*3 (G and H). Conditions are shown in Table S3 and S4.

3.3. ^{1}H NMR experiments

General remarks

Determination of the water content in organic solvents was performed by Karl–Fischer coulometry using a Metrohm 899 Coulometer with generator electrode with-out diaphragm. Anhydrous $CDCl_3$ (water content: 7 ppm) was prepared by storing commercially available $CDCl_3$ over 4 Å molecular sieves. Water-treated $CDCl_3$ (water content: 442 ppm) was prepared by stirring an emulsion of $CDCl_3$ with D_2O for 20 minutes. The organic phase was seperated and filtered through a nylon filter (20 µm). The water content in the water-enriched solvent was immediately measured. We avoided using water-saturated solvents in ^{1}H NMR experiments to ensure correct shimming and simpler data analysis. Data analysis was carried out in *MestReNova 14.0*, all spectra were referenced to the solvent residual resonance of $CDCl_3$ (δ_H = 7.26 ppm). All relevant signals were integrated with the *Data Analysis* tool implemented in *MestReNova 14.0*. The obtained integrals were referenced to the signals and corresponding concentration of 1,3,5-trimethoxy benzene (δ_H = 3.72 ppm, s, CH_3–O).

Bridge competition experiments

The reaction samples were prepared by mixing 9.7 mg of **3a** (0.06 mmol, 1 equiv.), 15.1 mg of **3b** (0.06 mmol, 1 equiv.), and 83.0 mg of **4a** (0.18 mmol, 3 equiv.) in $CDCl_3$ (6.0 mL). Subsequently, 0.6 mL of the reaction mixture was transferred into an NMR tube and the reaction was followed via ^{1}H NMR spectroscopy without stirring the sample.

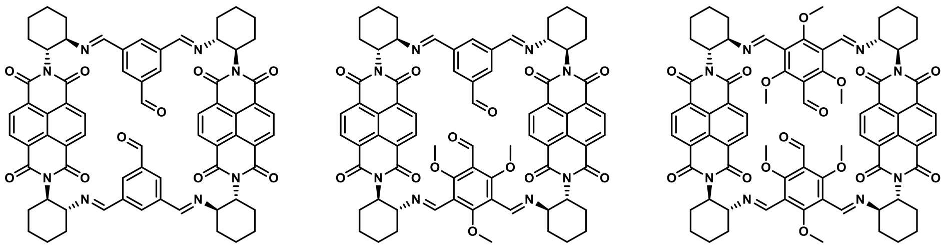

5a **5ab** **5b**

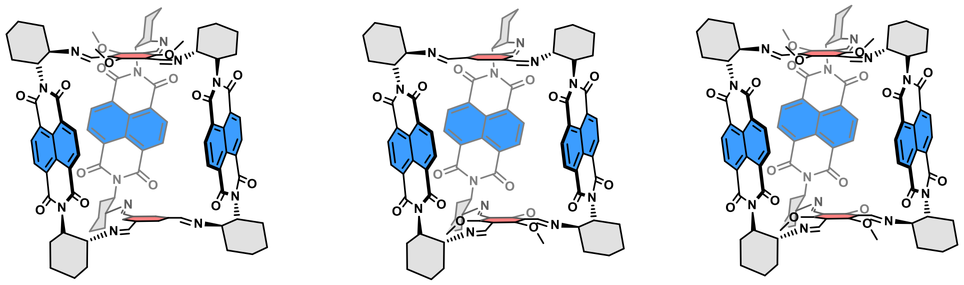

1a **1ab** **1b**

Scheme S1. Chemical structures of macrocycles **5a**, **5b**, and **5ab** as well as cages **1a**, **1b**, and **1ab**.

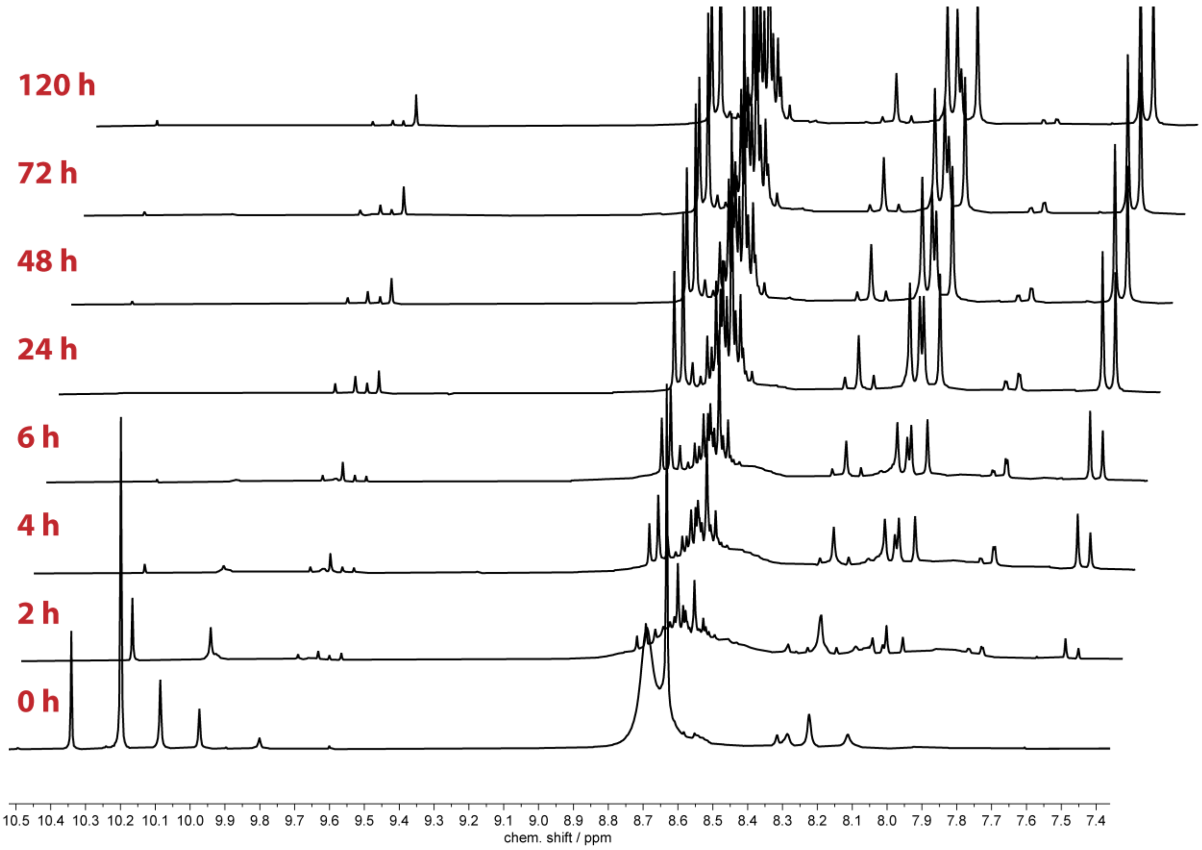

Fig. S51 ^{1}H NMR (300 MHz, 298 K, $CDCl_3$) of the mixture (2:2:6) of **3a**, **3b**, and **4a** monitored over five days with 76 ppm water concentration in $CDCl_3$.

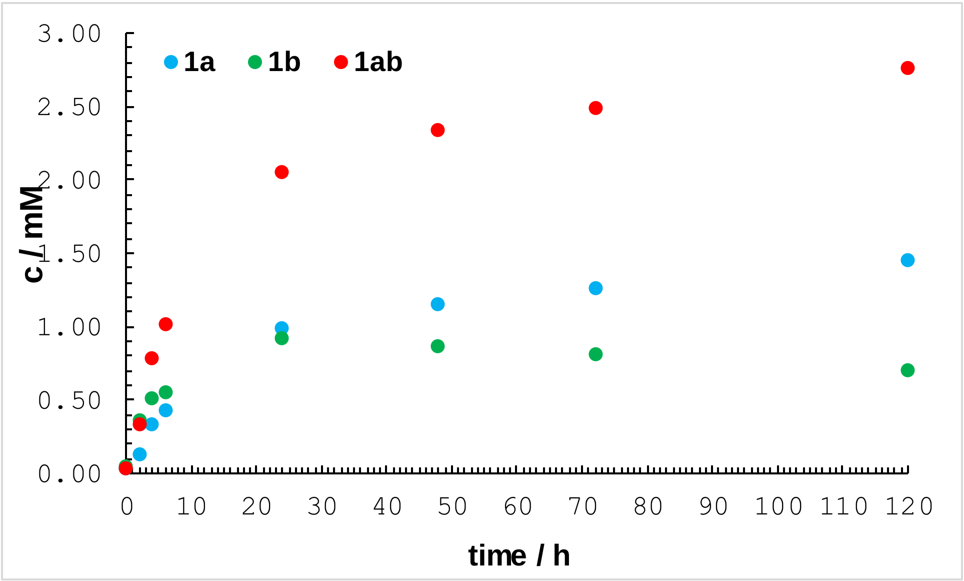

Fig. S52 Time-evolution curves of the ^{1}H NMR (300 MHz, 298 K, $CDCl_3$) of **1a**, **1b**, and **1ab** formed from the mixture (2:2:6) of **3a**, **3b**, and **4a** monitored over three days with 76 ppm water concentration in $CDCl_3$. Concentration is determined using tetramethylsilane as an internal standard.

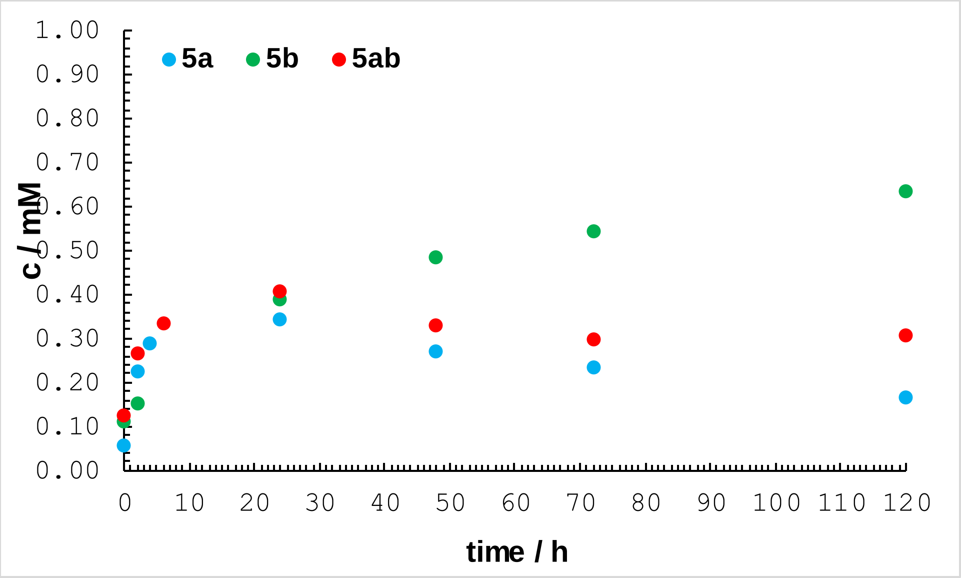

Fig. S53 Time-evolution curves of the ^{1}H NMR (300 MHz, 298 K, $CDCl_3$) of **5a**, **5b**, and **5ab** formed from the mixture (2:2:6) of **3a**, **3b**, and **4a** monitored over three days with 76 ppm water concentration in $CDCl_3$. Concentration is determined using tetramethylsilane as an internal standard.

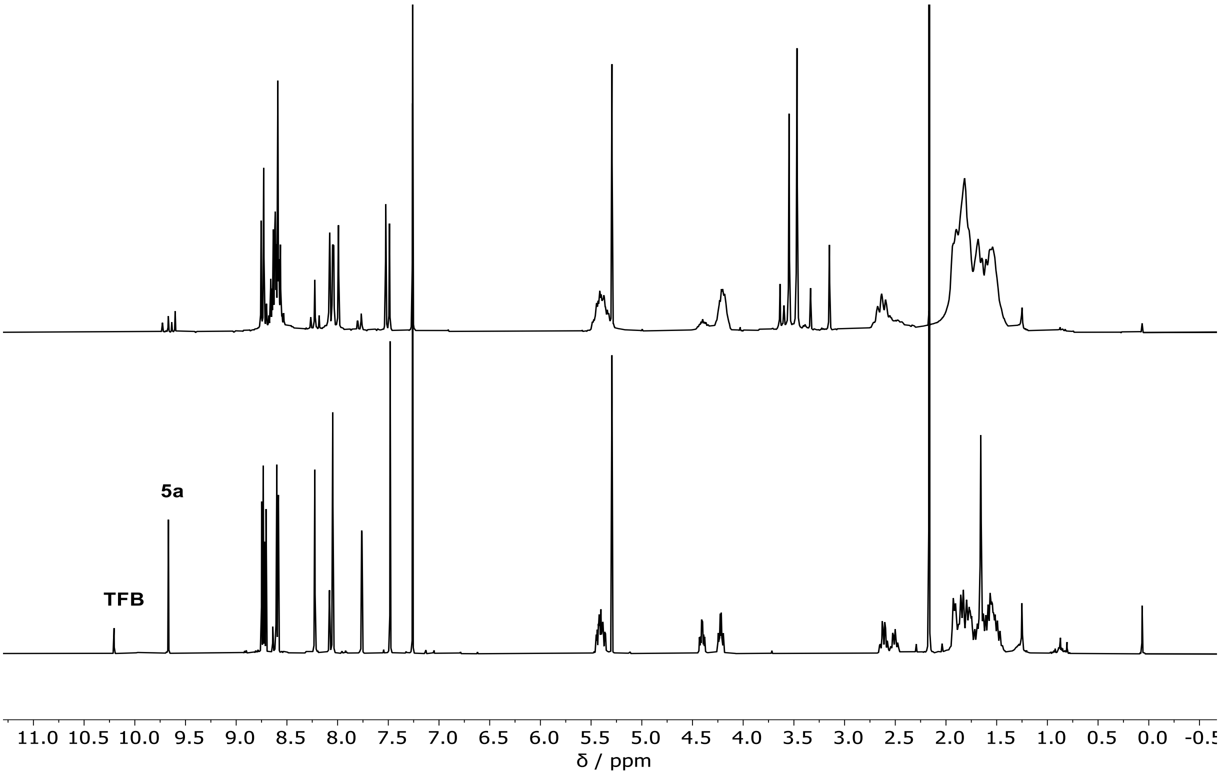

Fig. S54 ^{1}H NMR (300 MHz, 298 K, $CDCl_3$) after 24 h of the competition experiment shown in Figure S51 (top) and partially hydrolysed **1a** to **5a**[6] over SiO_2 (bottom).

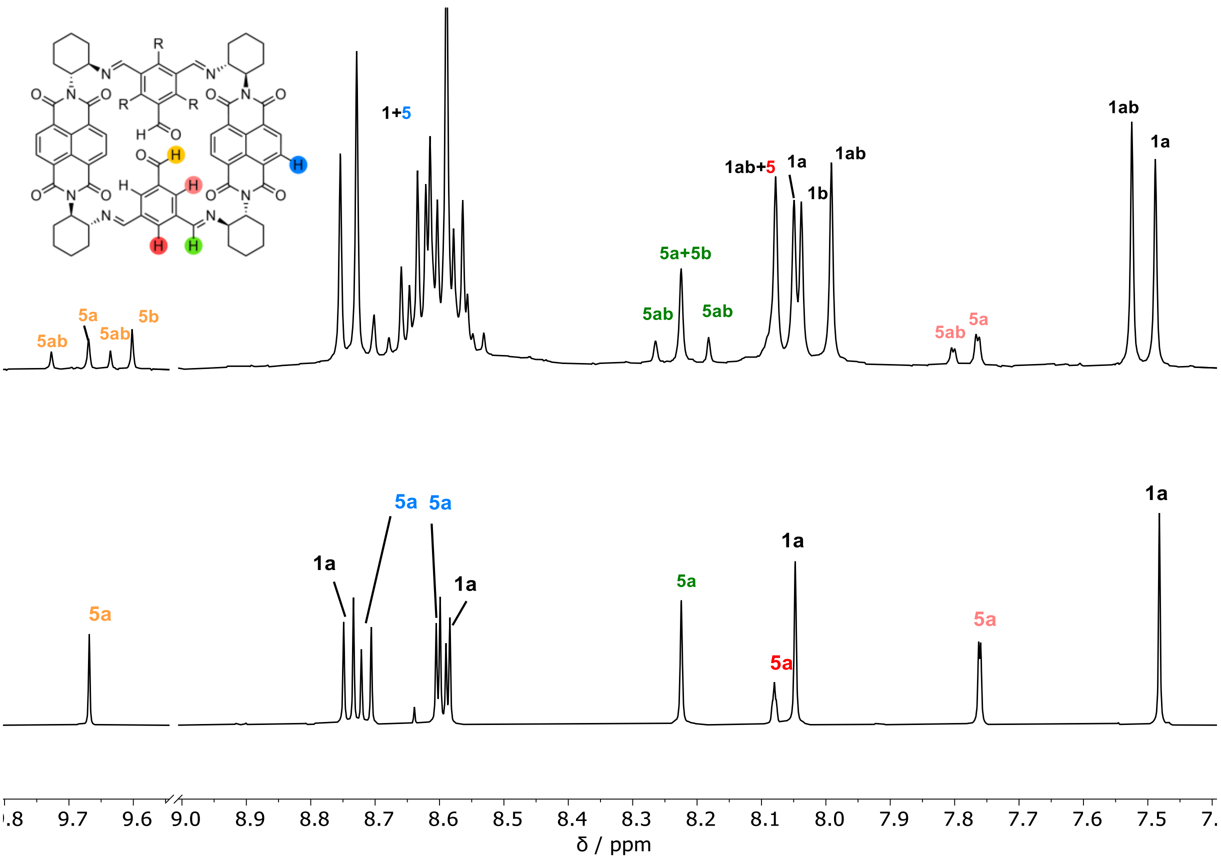

Fig. S55 ^{1}H NMR (300 MHz, 298 K, $CDCl_3$) of the aromatic region after 24 h of the competition experiment shown in Figure S51 (top) and partially hydrolysed **1a** to **5a**[6] over SiO_2 (bottom). Resonances for macrocycles **5** and cages **1** are assigned.

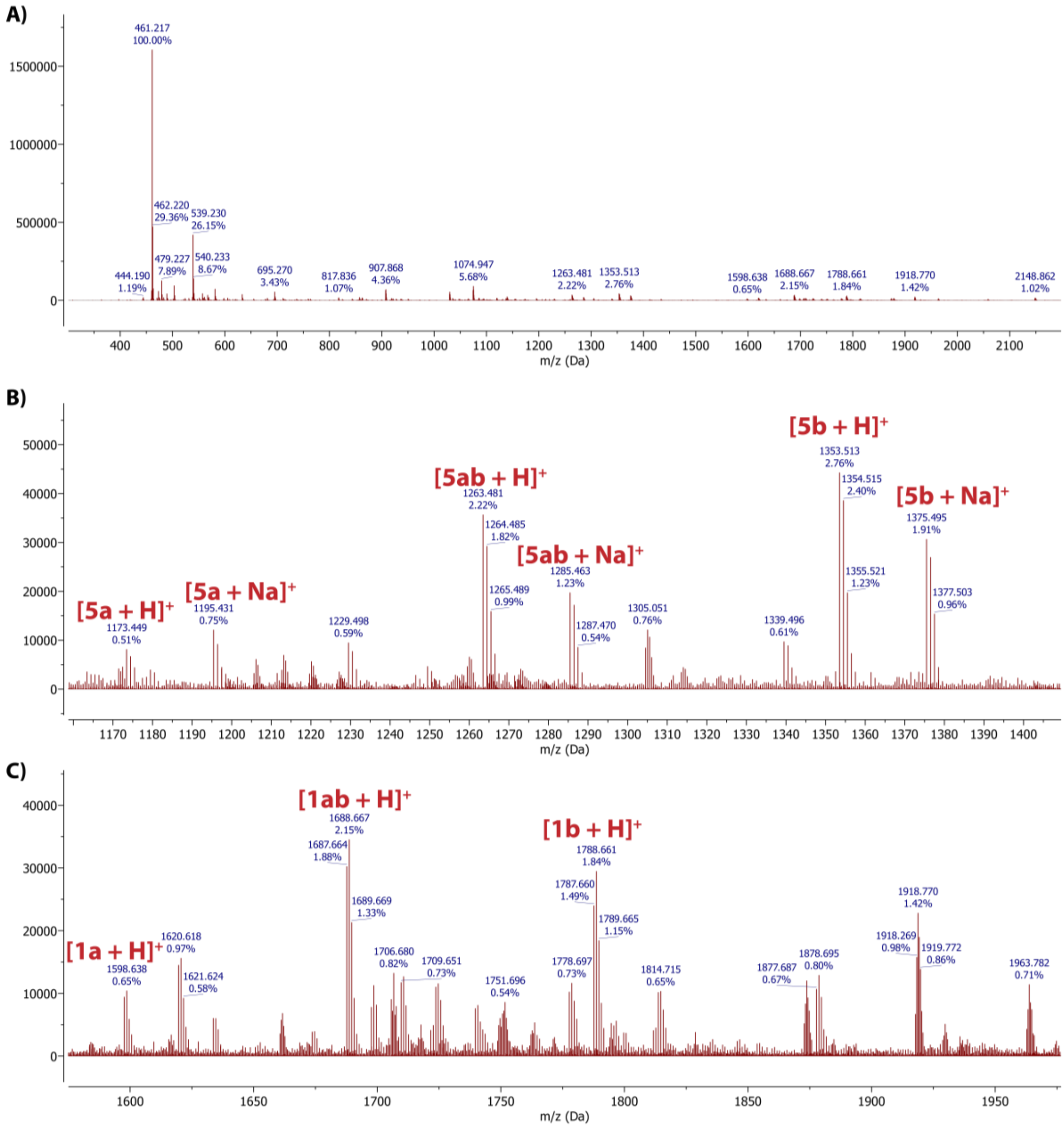

Fig. S56 A) Full HR-FD-MS of the crude reaction mixture between 2 equiv. **3a**, 2 equiv. **3b**, and 6 equiv. **4a** after 72 h. B) A zoomed-in spectrum to highlight compounds **5a**, **5ab**, and **5b** in the region of 1160–1410 m/z. C) A zoomed-in spectrum to highlight compounds **1a**, **1ab**, and **1b** in the region of 1550–2000 m/z. The corresponding structures of **1** and **5** are shown in Scheme S1.

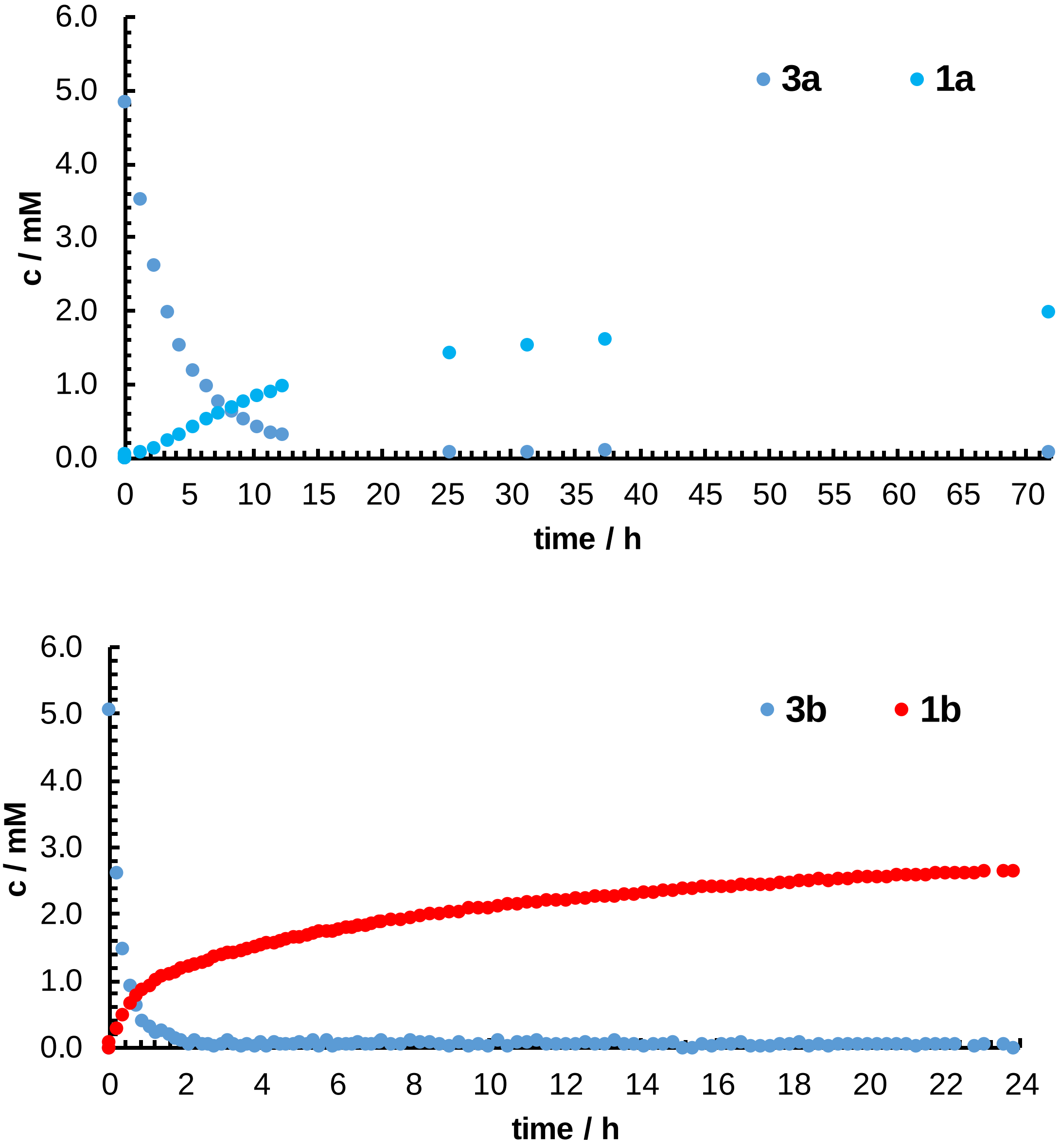

Fig. S57 Kinetic profiles of the cage formation monitored via ^{1}H NMR (300 MHz, 298 K, $CDCl_3$). Top: reaction of 2 equiv. **3a** with 3 equiv. of **4a** and the concentration profiles of **3a** and **1a**. Bottom: reaction of 2 equiv. **3b** with 3 equiv. of **4a** and the concentration profiles of **3b**, and **1b**. Concentration is determined using 1,3,5-trimethoxy benzene as an internal standard

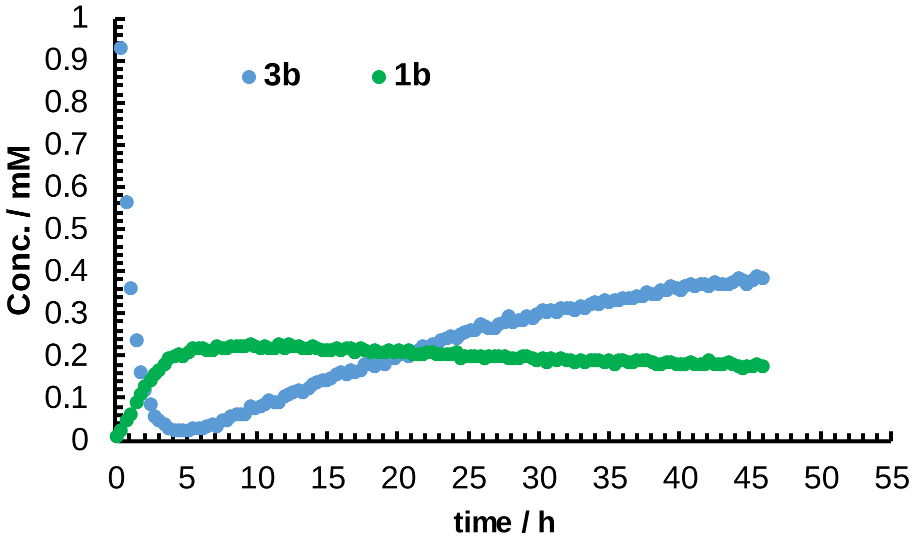

Fig. S58 Time-evolution curves of the ^{1}H NMR (300 MHz, 298 K, $CDCl_3$) of the mixture (2:2:3) of **3b**, and **1b** monitored over three days with 76 ppm water concentration in $CDCl_3$. Concentration is determined using 1,3,5-trimethoxy benzene as an internal standard

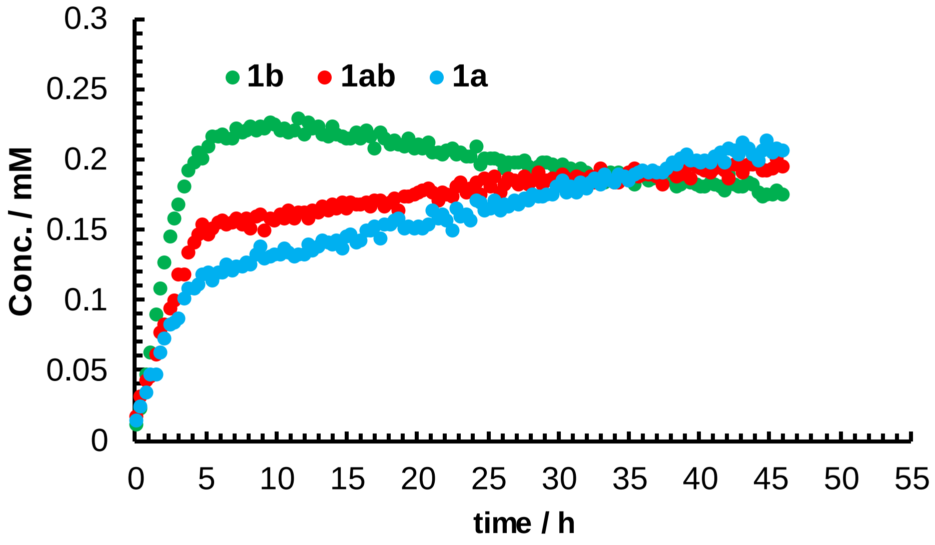

Fig. S59 Time-evolution curves of the ^{1}H NMR (300 MHz, 298 K, $CDCl_3$) of the mixture (2:2:3) of **1a**, **1b**, and **1ab** monitored over three days with 76 ppm water concentration in $CDCl_3$. Concentration is determined using 1,3,5-trimethoxy benzene as an internal standard

Bridge exchange experiments

The reaction samples were prepared by mixing the corresponding cage **1** (1 equiv., 5 mM), the corresponding aldehyde (6 equiv.), and 1,3,5-trimethoxy benzene (5 mg) in $CDCl_3$ (0.6 mL).

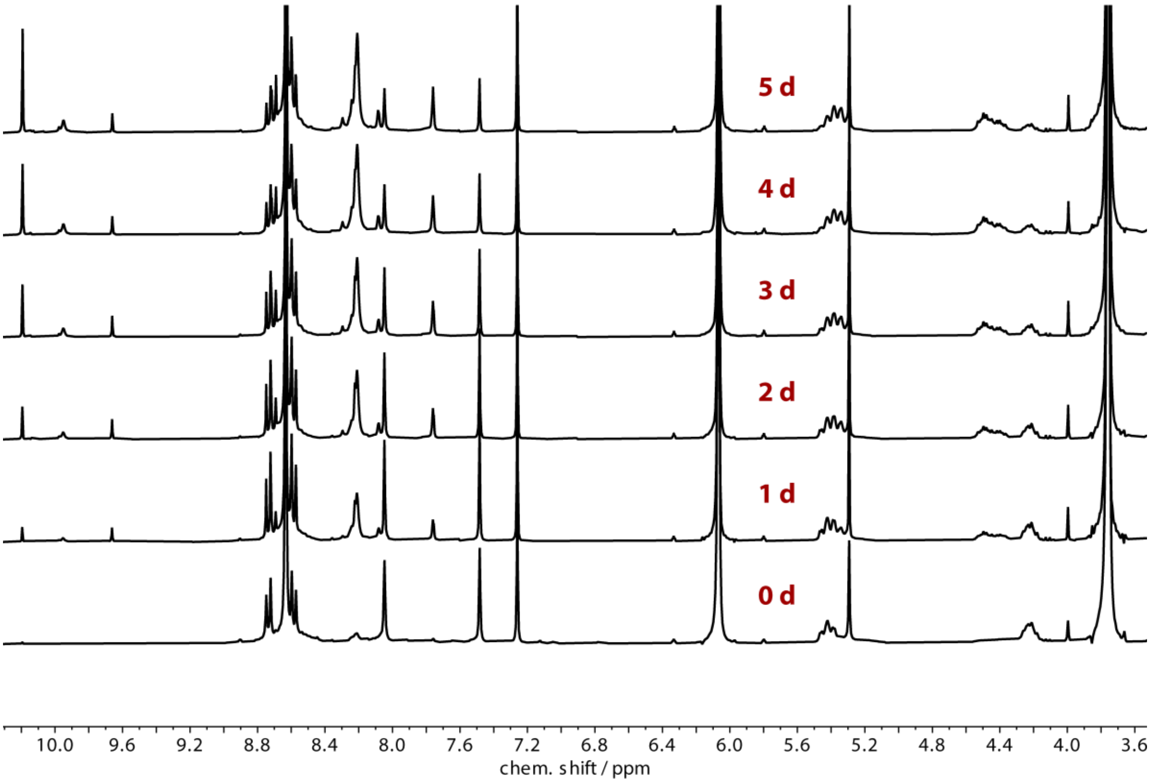

Fig. S60 ^{1}H NMR (300 MHz, 298 K, $CDCl_3$) of **1a** and 6 equiv. of **3a**-d_3 monitored over five days (red numbers) with 7 ppm water concentration in $CDCl_3$.

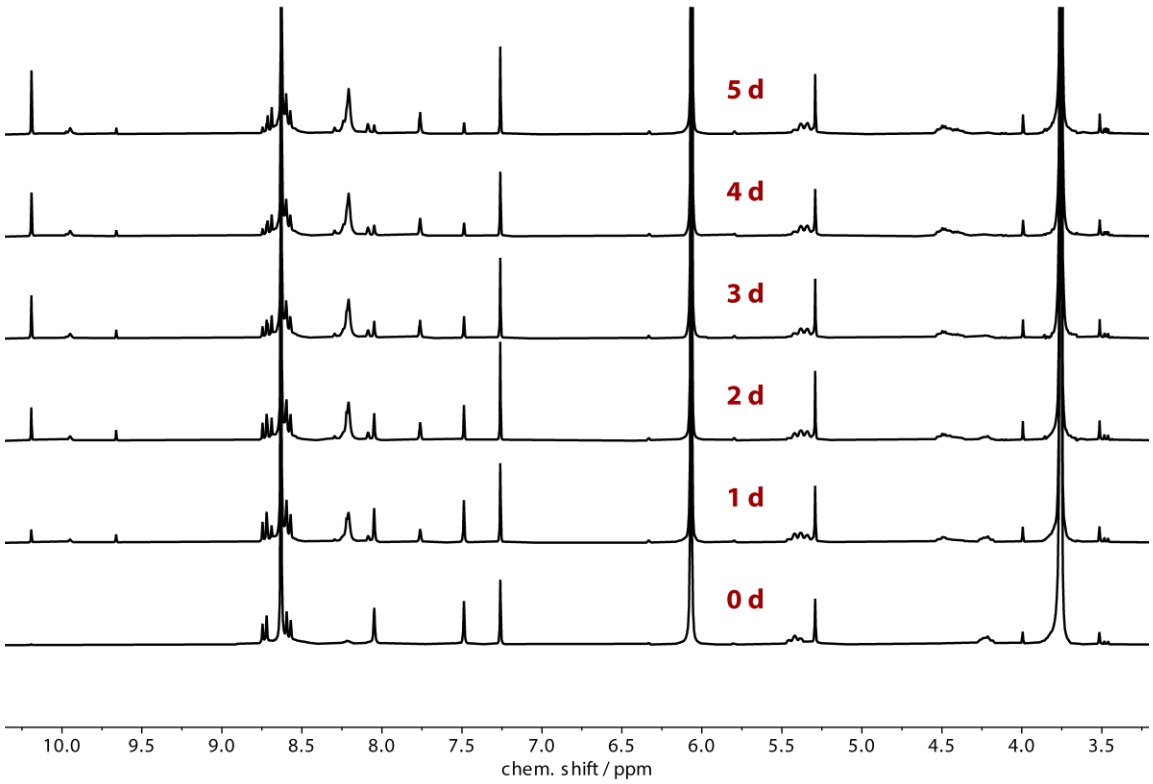

Fig. S61 ^{1}H NMR (300 MHz, 298 K, $CDCl_3$) of **1a** and 6 equiv. of **3a**-d_3 monitored over five days (red numbers) with 442 ppm water concentration in $CDCl_3$.

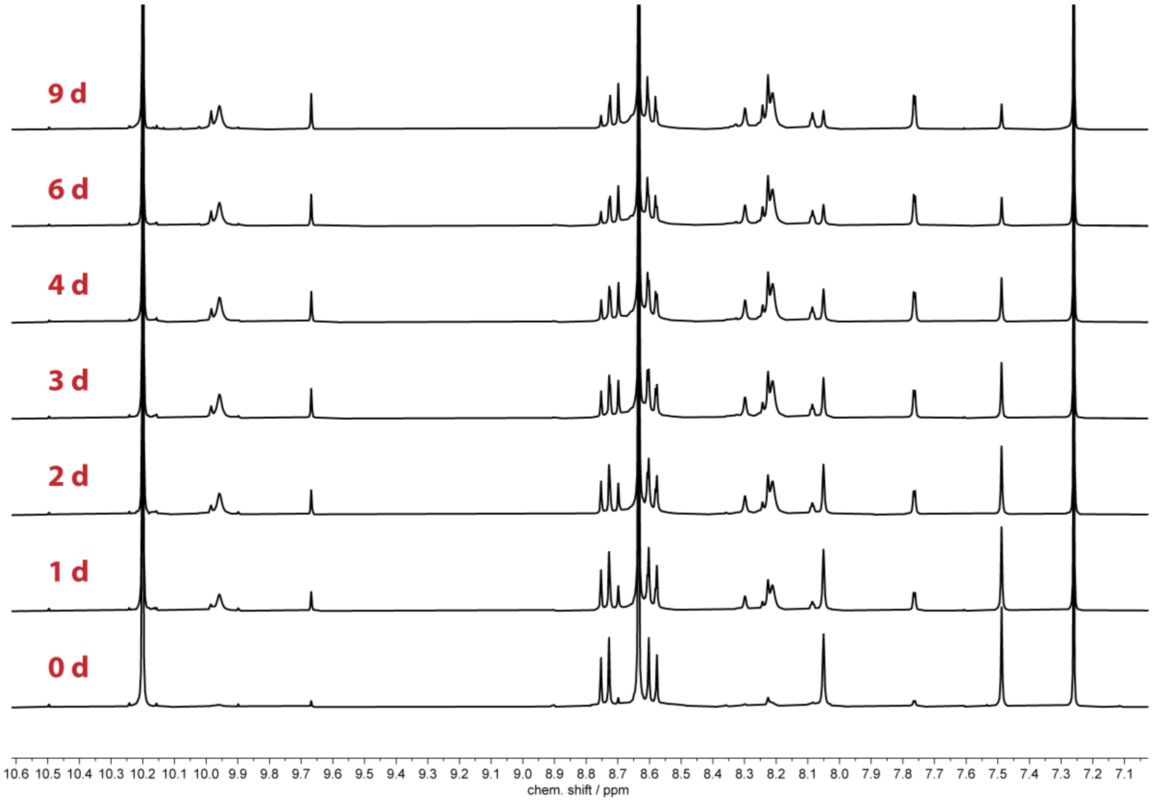

Fig. S62 ^{1}H NMR (300 MHz, 298 K, $CDCl_3$) of **1a** and 6 equiv. of **3a** monitored over nine days (red numbers) with 76 ppm water concentration in $CDCl_3$.

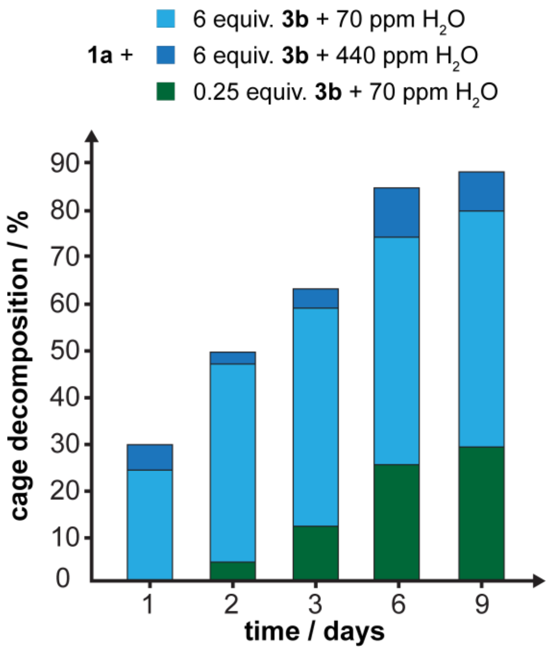

Fig. S63 Comparison of decomposition of **1a** with different amounts of **3b** and water in $CDCl_3$.

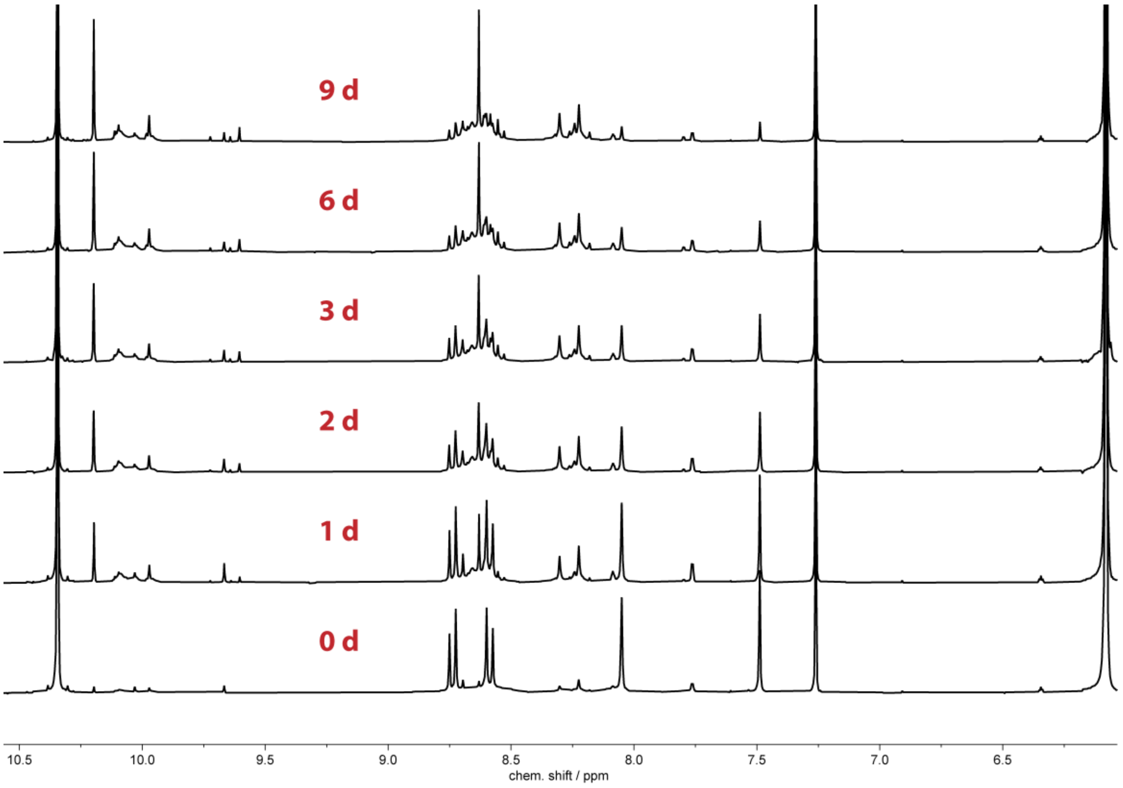

Fig. S64 ^{1}H NMR (300 MHz, 298 K, $CDCl_3$) of **1a** and 6 equiv. of **3b** monitored over nine days (red numbers) with 76 ppm water concentration in $CDCl_3$.

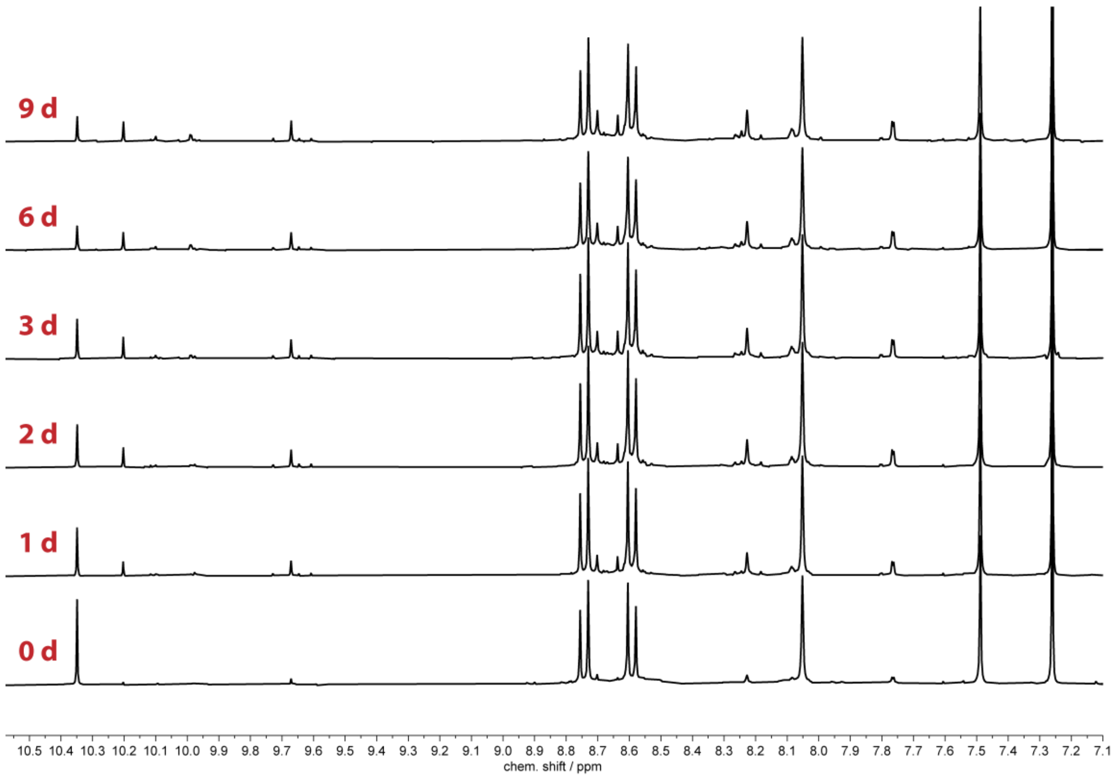

Fig. S65 ^{1}H NMR (300 MHz, 298 K, $CDCl_3$) of **1a** and 0.25 equiv. of **3b** monitored over nine days (r ed numbers) with 76 ppm water concentration in $CDCl_3$.

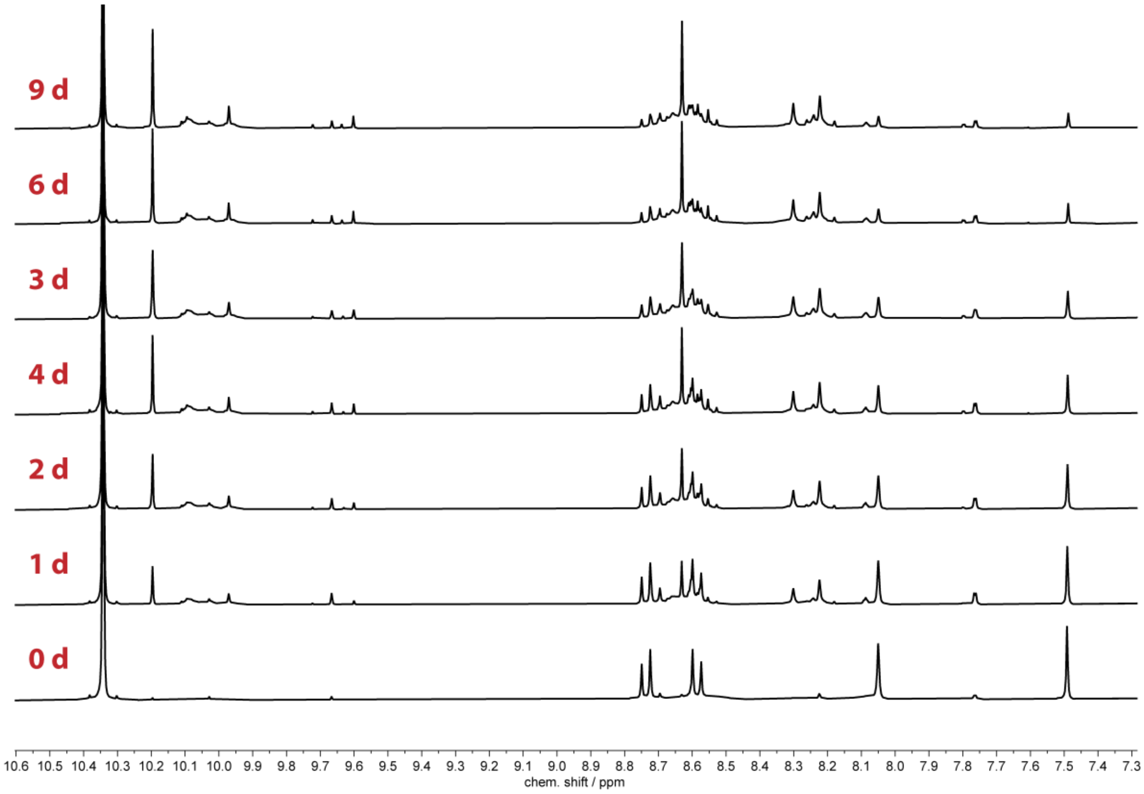

Fig. S66 ^{1}H NMR (300 MHz, 298 K, $CDCl_3$) of **1a** and 6 equiv. of **3b** monitored over nine days (red numbers) with 440 ppm water concentration in $CDCl_3$.

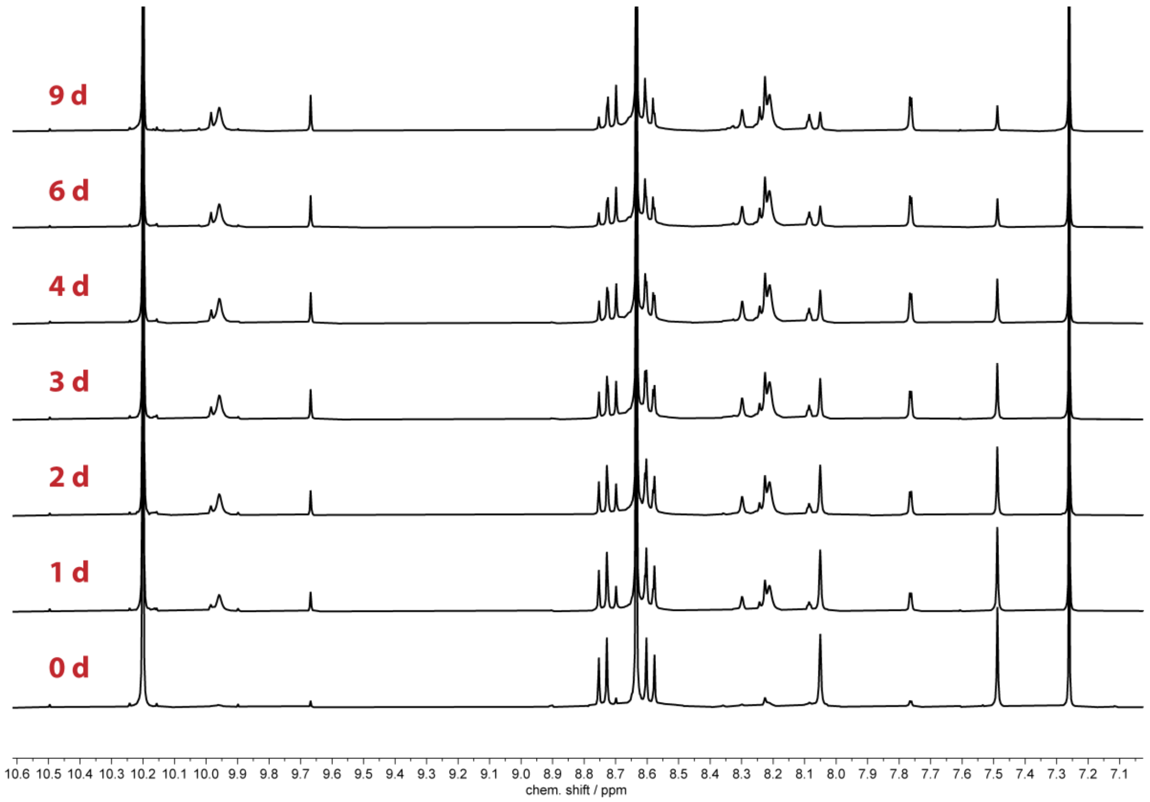

Fig. S67 ^{1}H NMR (300 MHz, 298 K, $CDCl_3$) of **1b** and 6 equiv. of **3a** monitored over nine days (red numbers) with 76 ppm water concentration in $CDCl_3$.

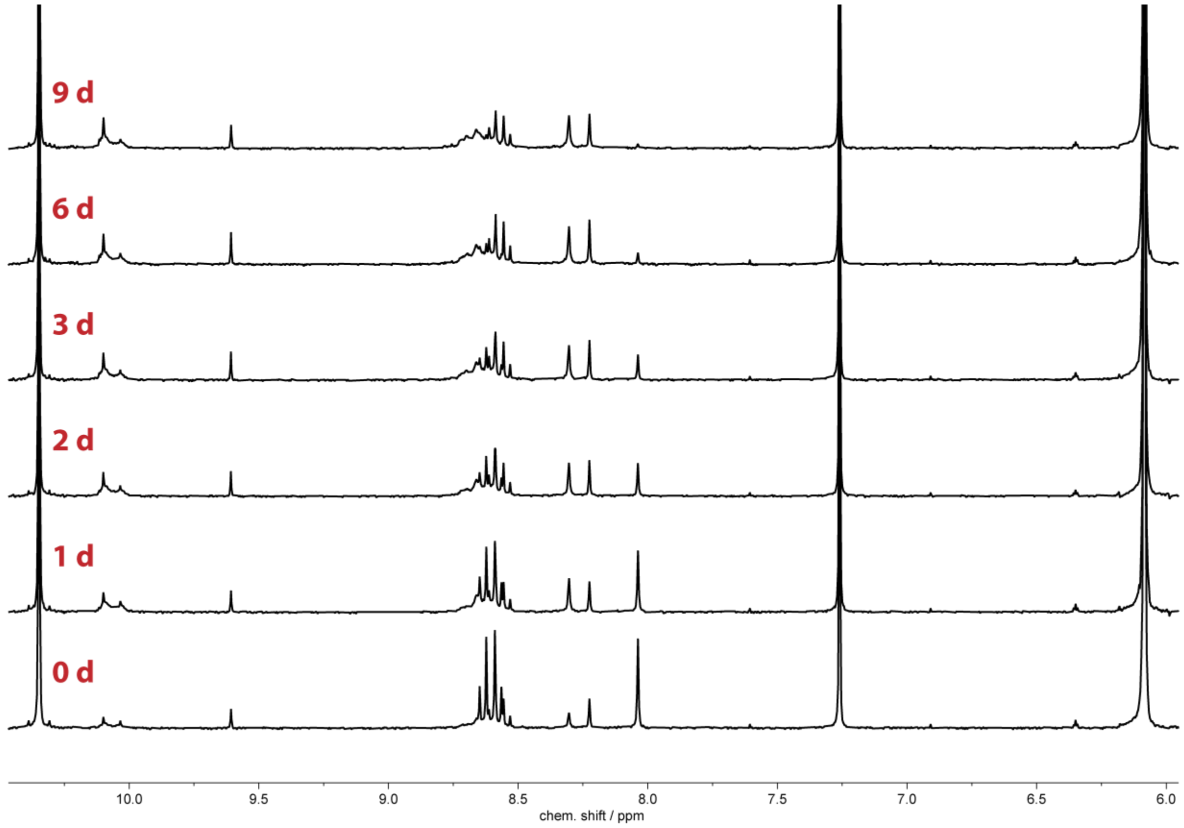

Fig. S68 ^{1}H NMR (300 MHz, 298 K, $CDCl_3$) of **1b** and 6 equiv. of **3b** monitored over nine days (red numbers) with 76 ppm water concentration in $CDCl_3$.

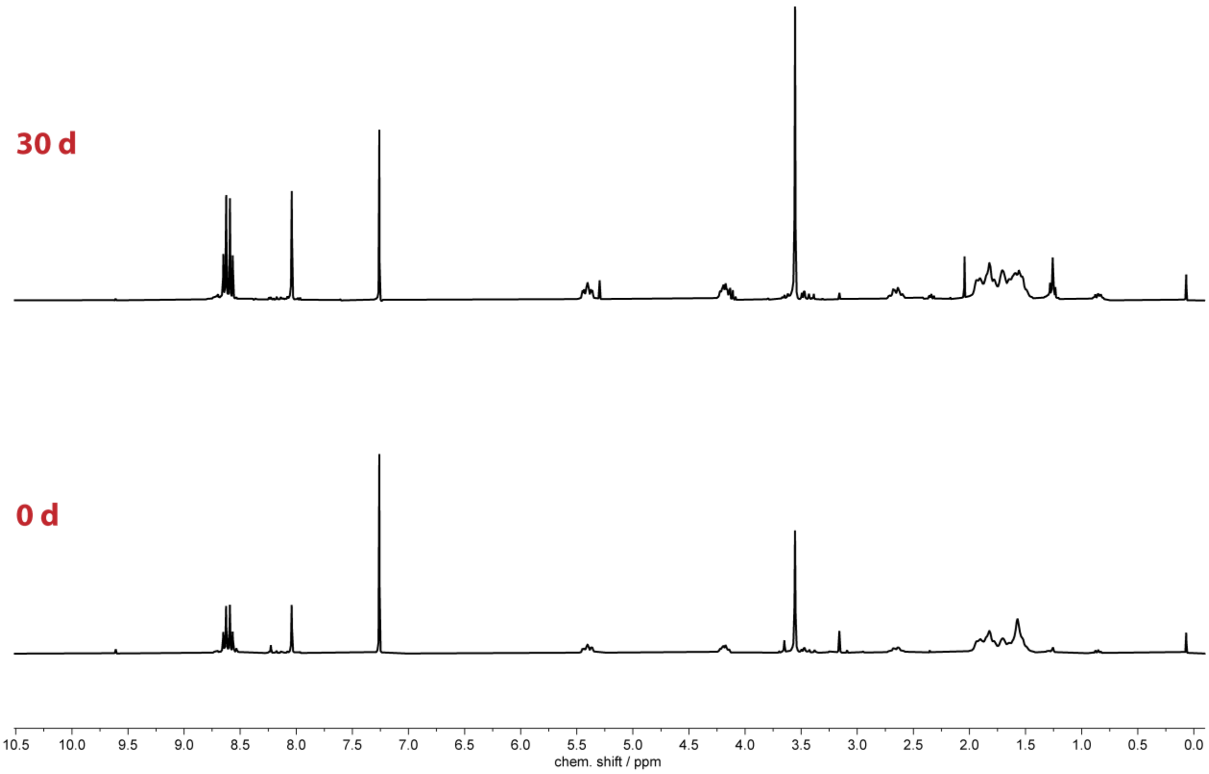

Fig. S69 ^{1}H NMR (300 MHz, 298 K, $CDCl_3$) of **1b** after isolation and after 30 days in solution with 76 ppm water concentration in $CDCl_3$.

3.4. Titration experiments

The samples were prepared by mixing stock solutions of the corresponding reagents. the corresponding cage (6.4 mM, 0.1 mL), and 1,3,5-trimethoxy benzene (213.1 mM, 0.45 mL) were mixed in $CDCl_3$ with the corresponding water concentration. The corresponding portion of a 2,2,2-trifluoroacetic acid (1.3 mM) stock solution was added and the sample measured.

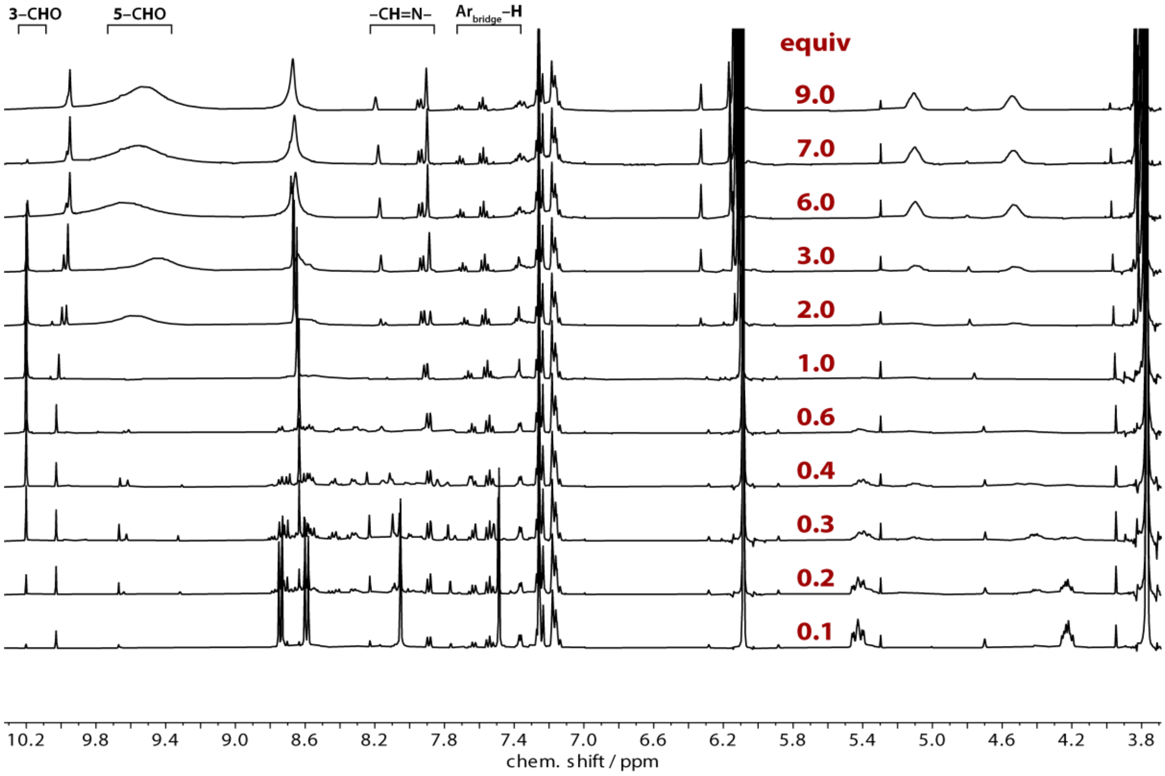

Fig. S70 ^{1}H NMR (300 MHz, 298 K, $CDCl_3$) of cage **1a** after addition (~10 min) of the appropriate amount of TFA (red numbers) with 7 ppm water concentration in $CDCl_3$.

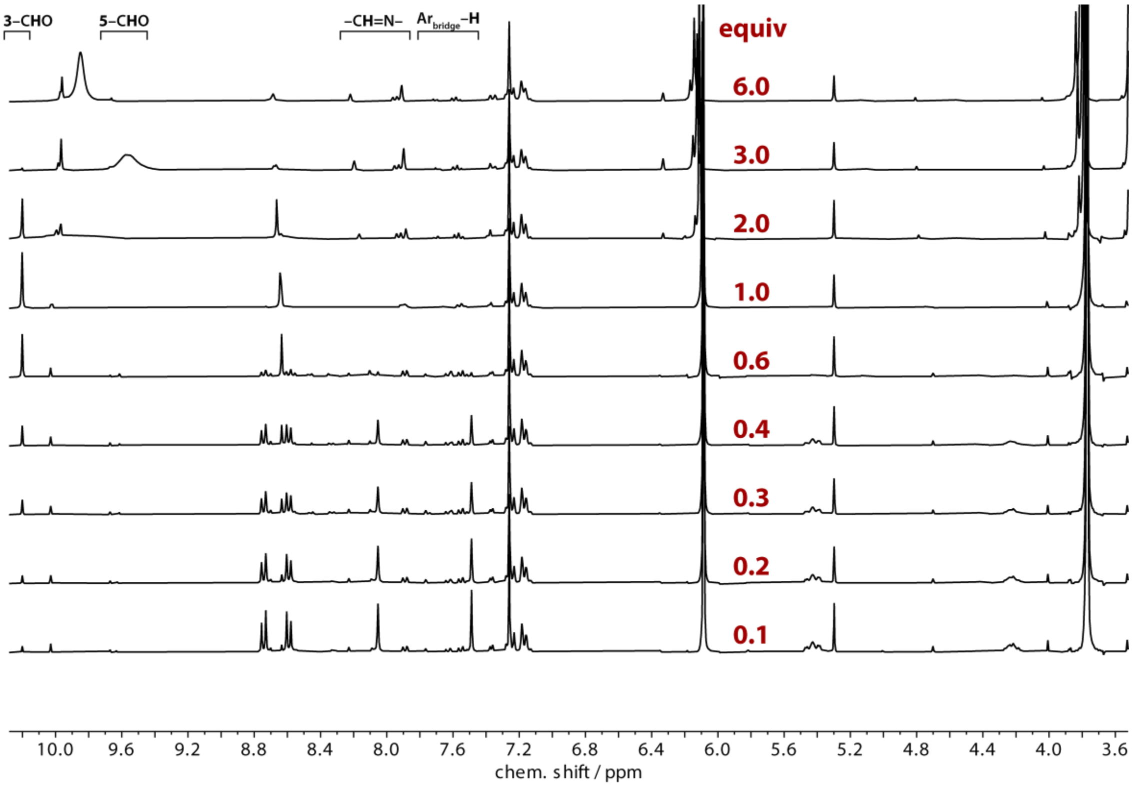

Fig. S71 ^{1}H NMR (300 MHz, 298 K, $CDCl_3$) of cage **1a** after the addition (~10 min) of the appropriate amount of TFA (red numbers) with 76 ppm water concentration in $CDCl_3$.

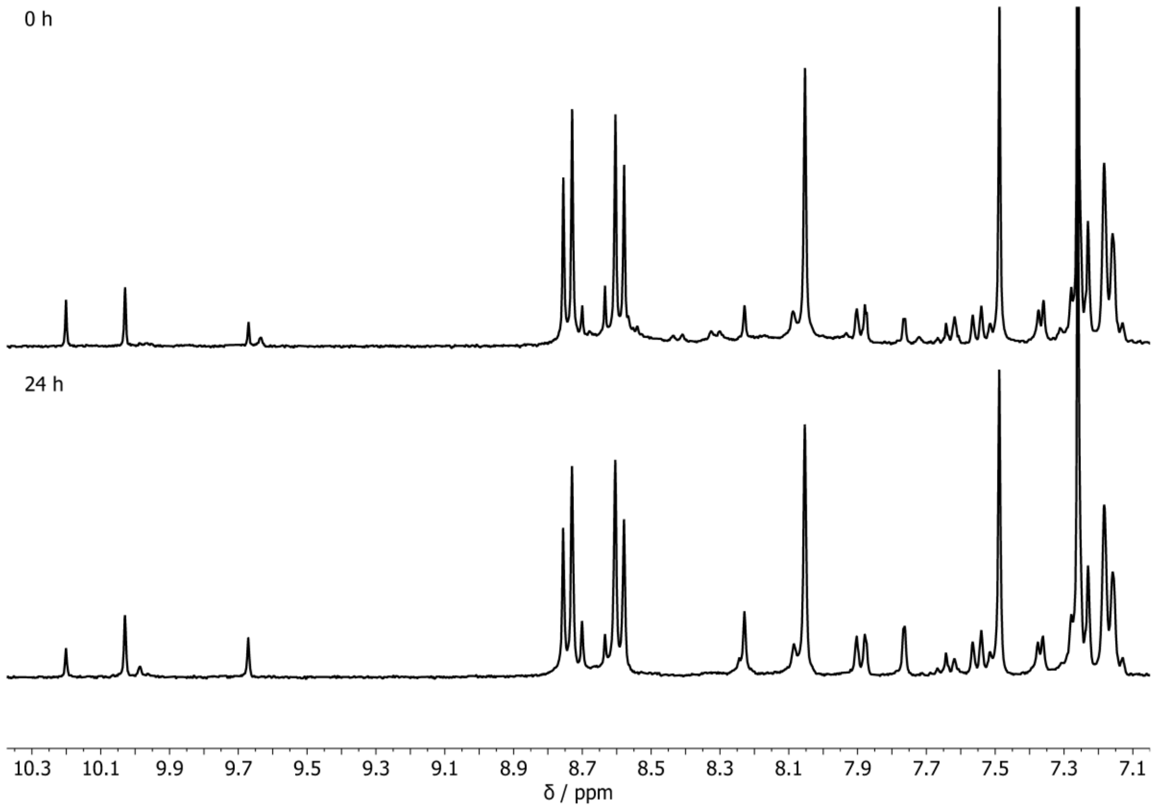

Fig. S72 ^{1}H NMR (300 MHz, 298 K, $CDCl_3$) of cage **1a** and 0.2 equiv. of TFA in right after the addition (~10 min) and after 24 h with 7 ppm water concentration in $CDCl_3$.

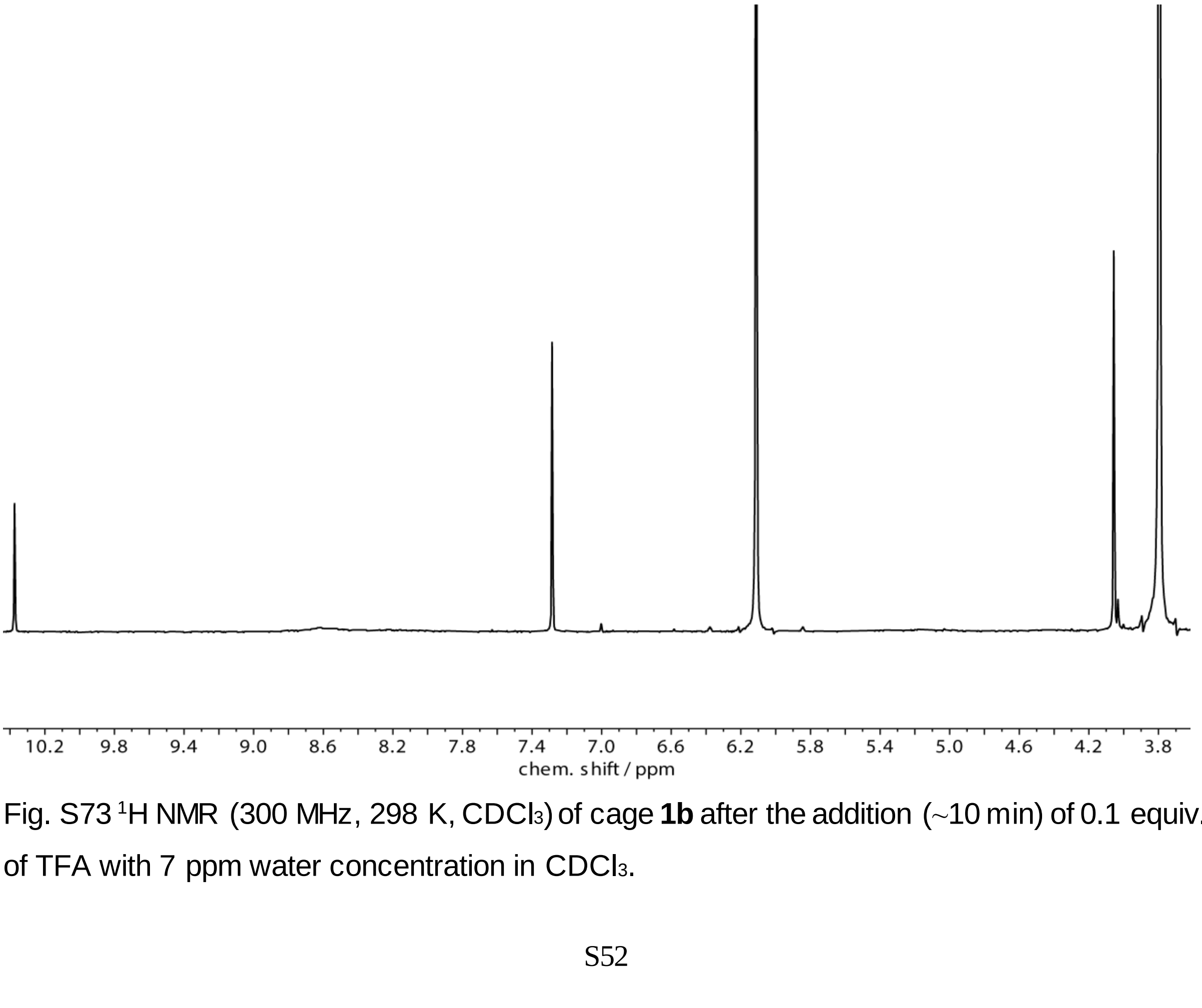

Fig. S73 ^{1}H NMR (300 MHz, 298 K, $CDCl_3$) of cage **1b** after the addition (~10 min) of 0.1 equiv. of TFA with 7 ppm water concentration in $CDCl_3$.

4. Computational methods

General remarks

All calculations were performed with Gaussian16 rev. C.02 suite of electronic structure programs.[13] The geometries of the potential energy minima were optimized at B3LYP/6-31G(d) level of theory and confirmed with frequency calculations. Please note that the bridges and cages were optimized in *C*3 and *D*3 symmetry, respectively. The single point energies were then calculated on M06-2X/6-31+G(2d,p) level of theory. The reported energies (at 0 K) given in kcal mol^{-1} represent the sum of the total electronic energy and the unscaled zero-point energy correction based on B3LYP/6-31G(d) level of theory. The heat of formation was calculated according to Eq. 1.

$$\Delta E = \left(E_{cage} + 6\,E_{water}\right) - \left(2\,E_{bridge} + 3\,E_{diamine}\right) \qquad \text{(Eq. 1)}$$

4.1. Cartesian coordinates

H_2O, Charge = 0, singlet, B3LYP/6-31G(d), *E* = –76.4399012298 Hartree, ZPVE = 0.021345 Hartree / Particle.

O	0.00000000	0.00000000	0.11691800
H	0.00000000	0.76579700	-0.46767300
H	0.00000000	-0.76579700	-0.46767300

4a, Charge = 0, singlet, B3LYP/6-31G(d), *E* = –1527.767501 Hartree, ZPVE = 0.514228 Hartree / Particle.

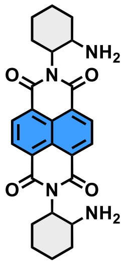

C	-5.67356600	-0.66492300	1.33768900
C	-5.02455600	0.26299700	0.29484000
C	-5.67374100	0.19941300	-1.10768700
C	-7.19779600	0.43120300	-0.95788000
C	-7.85806300	-0.48916700	0.07642500
C	-7.17394900	-0.34915800	1.44322100
N	-3.53457300	0.18368500	0.22271900
C	-2.87314800	1.42326000	0.11897700
C	-1.38946000	1.41394000	0.04140400
C	-0.69129500	0.18807100	0.04162100
C	-1.38694600	-1.03732900	0.11443500
C	-2.87458500	-1.05620600	0.19224300
C	0.72623100	0.18871200	-0.03598500
C	1.42497300	-1.03731000	-0.03764600
C	0.72388400	-2.22945500	0.03384400

C	-0.68160600	-2.22941600	0.10882100
C	1.41995400	1.41557800	-0.10881500
C	2.90479400	1.42779900	-0.18504700
N	3.56377500	0.18611000	-0.20716500
C	2.91065200	-1.04853000	-0.11510300
C	-0.69017200	2.60750800	-0.03324900
C	0.71518700	2.60811800	-0.10682800
C	5.05126400	0.21924100	-0.23818000
C	5.62724900	-0.42032500	-1.51335500
C	7.15244700	-0.24793600	-1.55933600
C	7.81294400	-0.81113200	-0.29407400
C	7.21547900	-0.17222200	0.96775900
C	5.68489800	-0.34254200	1.05327900
O	3.53414900	-2.09655200	-0.10660800
O	3.52282400	2.47799000	-0.23561500
O	-3.49708000	2.47114700	0.09782200
O	-3.47807600	-2.11211300	0.24069100
H	-5.27248900	1.04608000	-1.67838400
H	-7.36158000	1.47758400	-0.66232700
H	-7.66750000	0.31164800	-1.94226400
H	-8.92601500	-0.25539000	0.15575000
H	-7.79380000	-1.53431600	-0.25714700
H	-7.31789700	0.67393200	1.81957800
H	-7.63627700	-1.02080300	2.17551200
H	-5.18035600	-0.51132100	2.30486900
H	-5.54053200	-1.71637900	1.08294200
H	-1.24466800	3.53865700	-0.03358600
H	-1.23265000	-3.16102100	0.16228000
H	1.26579800	3.53987200	-0.16298300
H	1.27766000	-3.16112300	0.03115200
H	5.28157800	1.28621200	-0.27058700
H	5.37407600	-1.48363900	-1.54381200
H	5.16526200	0.05255400	-2.38791200
H	7.55076400	-0.74269900	-2.45252300
H	7.40082800	0.81873700	-1.65469800
H	7.66150800	-1.89896500	-0.25803100
H	8.89646300	-0.64647300	-0.32252400
H	7.66801200	-0.60419400	1.86911000
H	7.45267400	0.90263100	0.98251900
H	5.45599700	-1.40994200	1.11833200
H	-5.20113600	1.28254200	0.64072000
N	-5.28161100	-1.00745000	-1.84002700
H	-5.43117800	-1.85621500	-1.30420200
H	-5.80600700	-1.08502200	-2.70631900
N	5.06693900	0.29665900	2.22067500
H	5.37540100	-0.15527600	3.07659800
H	5.34338600	1.27437700	2.28594700

3a, Charge = 0, singlet, B3LYP/6-31G(d), *E* = –572.218000 Hartree, ZPVE = 0.128639 Hartree / Particle

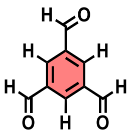

C	0.00000000	1.39684000	0.00013900
C	-1.21115100	0.70669300	0.00014500
C	-1.20969900	-0.69842000	0.00013900
C	-0.00643900	-1.40223400	0.00014500
C	1.20969900	-0.69842000	0.00013900

C	1.21759000	0.69554100	0.00014500
C	-2.49868800	1.44802000	0.00016900
C	-0.00467800	-2.88793700	0.00016900
C	2.50336600	1.43991800	0.00016900
O	-3.58917000	0.91349900	-0.00041900
O	1.00347200	-3.56506200	-0.00041900
O	2.58569800	2.65156300	-0.00041900
H	-2.40049000	2.55466800	0.00047400
H	3.41265200	0.80155100	0.00047400
H	-1.01216200	-3.35621900	0.00047400
H	-2.16463000	-1.21889100	0.00015100
H	0.02672400	2.48407000	0.00015100
H	2.13790600	-1.26517900	0.00015100

3b, Charge = 0, singlet, B3LYP/6-31G(d), *E* = –915.756322 Hartree, ZPVE = 0.225909 Hartree / Particle.

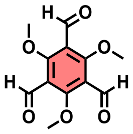

C	-1.34182600	0.40752000	-0.23496700
C	-0.32405700	1.38691000	-0.23473200
C	1.02383600	0.95829500	-0.23496700
C	1.36312800	-0.41281300	-0.23473200
C	0.31799000	-1.36581500	-0.23496700
C	-1.03907000	-0.97409700	-0.23473200
C	-0.72078100	2.81574200	-0.34118000
C	2.79889400	-0.78365600	-0.34118000
C	-2.07811300	-2.03208500	-0.34118000
O	-2.62865700	0.83858000	-0.31351200
O	2.04056000	1.85719400	-0.31351200
O	0.58809700	-2.69577400	-0.31351200
O	0.00000000	3.77436200	-0.12902300
O	3.26869300	-1.88718100	-0.12902300
O	-3.26869300	-1.88718100	-0.12902300
H	-1.76968200	2.96127900	-0.65282100
H	-1.67970200	-3.01322900	-0.65282100
H	3.44938400	0.05195000	-0.65282100
C	-3.38108500	0.88862600	0.91091100
H	-4.36361400	1.27187400	0.63260200
H	-3.48625500	-0.10965400	1.33840800
H	-2.90383300	1.57474900	1.62189900
C	0.92097000	-3.37241800	0.91091100
H	0.08814400	-3.30216800	1.62189900
H	1.08033200	-4.41493700	0.63260200
H	1.83809100	-2.96435900	1.33840800
C	2.46011500	2.48379200	0.91091100
H	3.28328200	3.14306300	0.63260200
H	1.64816400	3.07401300	1.33840800
H	2.81568900	1.72741900	1.62189900

3c, Charge = 0, singlet, B3LYP/6-31G(d), *E* = –1884.679493 Hartree, ZPVE = 0.213467 Hartree / Particle.

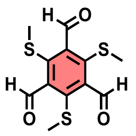

C	-1.20718100	0.71594600	-0.46634300
C	0.01544900	1.40084200	-0.46184400
C	1.22361800	0.68747600	-0.46634300
C	1.20544000	-0.71380000	-0.46184400
C	-0.01643700	-1.40342200	-0.46634300
C	-1.22088900	-0.68704100	-0.46184400
C	0.00000000	2.91301100	-0.39116300
C	2.52274200	-1.45650600	-0.39116300
C	-2.52274200	-1.45650600	-0.39116300
O	0.27068100	3.51492800	0.62608400
O	2.90867600	-1.99188100	0.62608400
O	-3.17935800	-1.52304700	0.62608400
H	-0.28295800	3.44454200	-1.31957200
H	-2.84158200	-1.96732000	-1.31957200
H	3.12454000	-1.47722200	-1.31957200
C	-3.08468600	1.81365300	1.27935100
H	-4.00703000	2.39378800	1.36683200
H	-3.22841100	0.81947300	1.70684900
H	-2.27098200	2.34422600	1.77975400
C	-0.02832700	-3.57824300	1.27935100
H	-0.89466900	-3.13884200	1.77975400
H	-0.06956600	-4.66708400	1.36683200
H	0.90452100	-3.20562200	1.70684900
C	3.11301300	1.76458900	1.27935100
H	4.07659600	2.27329600	1.36683200
H	2.32389000	2.38614900	1.70684900
H	3.16565100	0.79461500	1.77975400
S	-2.73794000	1.66453500	-0.51890800
S	2.81049900	1.53885800	-0.51890800
S	-0.07255900	-3.20339300	-0.51890800

1a, Charge = 0, singlet, B3LYP/6-31G(d), E = –5269.22643619 Hartree, ZPVE = 1.660162 Hartree / Particle.

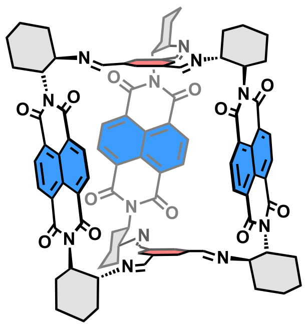

C	0.71358500	1.21099700	5.03651700
C	-0.71358500	1.21099700	-5.03651700
C	-1.40554700	0.01248400	5.03651700
C	1.40554700	0.01248400	-5.03651700
C	-0.70242300	-1.20662000	5.03166600
C	0.70242300	-1.20662000	-5.03166600
C	0.69196200	-1.22348100	5.03651700
C	-0.69196200	-1.22348100	-5.03651700

C	1.39617500	-0.00500600	5.03166600
C	-1.39617500	-0.00500600	-5.03166600
C	0.69375200	1.21162600	-5.03166600
C	-0.69375200	1.21162600	5.03166600
C	-1.47208600	2.47363200	-5.12211500
C	1.47208600	2.47363200	5.12211500
N	-0.90371400	3.59287100	-5.34108900
N	0.90371400	3.59287100	5.34108900
N	2.65966100	-2.57907500	5.34108900
N	-2.65966100	-2.57907500	-5.34108900
N	-3.56337500	-1.01379600	5.34108900
N	3.56337500	-1.01379600	-5.34108900
N	-0.75173800	5.84710400	-3.47137400
N	0.75173800	5.84710400	3.47137400
N	4.68787200	-3.57457600	3.47137400
N	-4.68787200	-3.57457600	-3.47137400
N	5.43961000	-2.27252800	-3.47137400
N	-5.43961000	-2.27252800	3.47137400
C	-4.67683600	-3.89797200	-4.92806900
C	4.67683600	-3.89797200	4.92806900
C	-1.74338100	4.76805000	-5.53536500
C	1.74338100	4.76805000	5.53536500
C	-1.03732500	5.99924500	-4.92806900
C	1.03732500	5.99924500	4.92806900
C	-1.76658200	7.32009100	-5.23172800
C	1.76658200	7.32009100	5.23172800
C	-1.93426100	7.50612500	-6.74683500
C	1.93426100	7.50612500	6.74683500
C	-2.65556800	6.30428000	-7.37066900
C	2.65556800	6.30428000	7.37066900
C	-1.92211400	4.99423700	-7.05532600
C	1.92211400	4.99423700	7.05532600
C	-1.82320600	5.76076100	-2.57070500
C	1.82320600	5.76076100	2.57070500
C	-1.49714400	5.73459200	-1.11811400
C	1.49714400	5.73459200	1.11811400
C	-0.14894400	5.73881200	-0.69433300
C	0.14894400	5.73881200	0.69433300
C	0.90630200	5.75733500	-1.63426900
C	-0.90630200	5.75733500	1.63426900
C	0.60865900	5.83622000	-3.09039200
C	-0.60865900	5.83622000	3.09039200
C	-2.51741500	5.72718700	-0.17950000
C	2.51741500	5.72718700	0.17950000
C	-2.22196000	5.74618100	1.19707300
C	2.22196000	5.74618100	-1.19707300
O	2.98422200	5.72938700	2.95815100
O	-2.98422200	5.72938700	-2.95815100
O	1.50575700	5.91613100	-3.91532200
O	-1.50575700	5.91613100	3.91532200
O	3.46968400	-5.44910600	2.95815100
O	-3.46968400	-5.44910600	-2.95815100
O	5.87639800	-1.65404200	3.91532200
O	-5.87639800	-1.65404200	-3.91532200
O	6.45390600	-0.28028100	-2.95815100
O	-6.45390600	-0.28028100	2.95815100
O	4.37064100	-4.26208900	-3.91532200
O	-4.37064100	-4.26208900	3.91532200
C	-5.53336400	-5.42818200	-6.74683500
C	5.53336400	-5.42818200	6.74683500

C	-4.13188300	-5.45192900	-7.37066900
C	4.13188300	-5.45192900	7.37066900
C	-3.36407900	-4.16171800	-7.05532600
C	3.36407900	-4.16171800	7.05532600
C	-2.87827100	0.03804800	5.12211500
C	2.87827100	0.03804800	-5.12211500
C	-5.00094300	-0.87421300	5.53536500
C	5.00094300	-0.87421300	-5.53536500
C	-6.78745100	-0.85235100	7.37066900
C	6.78745100	-0.85235100	-7.37066900
C	-5.71416100	-2.10127300	4.92806900
C	5.71416100	-2.10127300	-4.92806900
C	4.89548500	-2.99839500	0.69433300
C	-4.89548500	-2.99839500	-0.69433300
C	5.43914900	-2.09378700	1.63426900
C	-5.43914900	-2.09378700	-1.63426900
C	5.04442900	-2.74041700	-0.69433300
C	-5.04442900	-2.74041700	0.69433300
C	5.71487400	-1.57073100	-1.11811400
C	-5.71487400	-1.57073100	1.11811400
C	7.46762500	-2.07794300	-6.74683500
C	-7.46762500	-2.07794300	6.74683500
C	5.28619300	-0.83251900	-7.05532600
C	-5.28619300	-0.83251900	7.05532600
C	7.22267600	-2.13014100	-5.23172800
C	-7.22267600	-2.13014100	5.23172800
C	-5.90056800	-1.30143800	2.57070500
C	5.90056800	-1.30143800	-2.57070500
C	-4.53284700	-3.66354800	1.63426900
C	4.53284700	-3.66354800	-1.63426900
C	-4.74998500	-3.44522400	3.09039200
C	4.74998500	-3.44522400	-3.09039200
C	-6.08731900	-0.94881700	-1.19707300
C	6.08731900	-0.94881700	1.19707300
C	-4.21773000	-4.16386100	-1.11811400
C	4.21773000	-4.16386100	1.11811400
C	-3.86535900	-4.79736400	1.19707300
C	3.86535900	-4.79736400	-1.19707300
C	-6.21859700	-0.68344800	0.17950000
C	6.21859700	-0.68344800	-0.17950000
C	-3.70118200	-5.04373900	-0.17950000
C	3.70118200	-5.04373900	0.17950000
C	-4.07736200	-4.45932300	-2.57070500
C	4.07736200	-4.45932300	2.57070500
C	-5.35864400	-2.39099600	-3.09039200
C	5.35864400	-2.39099600	3.09039200
C	-3.25756200	-3.89383700	-5.53536500
C	3.25756200	-3.89383700	5.53536500
C	5.45609400	-5.18995000	5.23172800
C	-5.45609400	-5.18995000	-5.23172800
C	-1.40618500	-2.51168000	-5.12211500
C	1.40618500	-2.51168000	5.12211500
H	-7.25723500	0.06221400	6.98156800
H	7.25723500	0.06221400	-6.98156800
H	3.57473900	-6.31605700	6.98156800
H	-3.57473900	-6.31605700	-6.98156800
H	3.68249600	6.25384300	6.98156800
H	-3.68249600	6.25384300	-6.98156800
H	-7.07378900	-2.99109900	7.21620800
H	7.07378900	-2.99109900	-7.21620800

H	6.12726200	-4.63053100	7.21620800
H	-6.12726200	-4.63053100	-7.21620800
H	0.94652700	7.62163000	7.21620800
H	-0.94652700	7.62163000	-7.21620800
H	-8.54608900	-2.06207000	6.94616500
H	8.54608900	-2.06207000	-6.94616500
H	6.05885000	-6.37009500	6.94616500
H	-6.05885000	-6.37009500	-6.94616500
H	2.48723900	8.43216500	6.94616500
H	-2.48723900	8.43216500	-6.94616500
H	-7.65170800	-3.04631200	4.80707600
H	7.65170800	-3.04631200	-4.80707600
H	6.46403800	-5.10341700	4.80707600
H	-6.46403800	-5.10341700	-4.80707600
H	1.18767000	8.14973000	4.80707600
H	-1.18767000	8.14973000	-4.80707600
H	-7.71752600	-1.28222300	4.74870500
H	7.71752600	-1.28222300	-4.74870500
H	4.96920100	-6.04246200	4.74870500
H	-4.96920100	-6.04246200	-4.74870500
H	2.74832500	7.32468500	4.74870500
H	-2.74832500	7.32468500	-4.74870500
H	-5.25181300	-2.98063200	5.37854600
H	5.25181300	-2.98063200	-5.37854600
H	-5.20721000	-3.05788700	-5.37854600
H	5.20721000	-3.05788700	5.37854600
H	0.04460300	6.03851900	5.37854600
H	-0.04460300	6.03851900	-5.37854600
H	-6.72711900	0.20905900	0.52780500
H	6.72711900	0.20905900	-0.52780500
H	3.18250900	-5.93038500	0.52780500
H	-3.18250900	-5.93038500	-0.52780500
H	3.54461000	5.72132600	0.52780500
H	-3.54461000	5.72132600	-0.52780500
H	-3.48377000	-5.49347100	1.93615700
H	3.48377000	-5.49347100	-1.93615700
H	-3.01560000	5.76376900	1.93615700
H	3.01560000	5.76376900	-1.93615700
H	-6.49937000	-0.27029800	-1.93615700
H	6.49937000	-0.27029800	1.93615700
H	6.93588400	-0.84372400	-8.45764700
H	-6.93588400	-0.84372400	8.45764700
H	4.19862800	-5.58479000	8.45764700
H	-4.19862800	-5.58479000	-8.45764700
H	2.73725600	6.42851400	8.45764700
H	-2.73725600	6.42851400	-8.45764700
H	4.81334900	0.06535800	-7.47125000
H	-4.81334900	0.06535800	7.47125000
H	-2.46327600	4.13580400	-7.47125000
H	2.46327600	4.13580400	7.47125000
H	4.79126200	-1.69435900	-7.52362600
H	-4.79126200	-1.69435900	7.52362600
H	-0.92827300	4.99653400	-7.52362600
H	0.92827300	4.99653400	7.52362600
H	3.86298900	-3.30217500	7.52362600
H	-3.86298900	-3.30217500	-7.52362600
H	2.35007300	-4.20116100	7.47125000
H	-2.35007300	-4.20116100	-7.47125000
H	-2.65285300	-4.68919300	-5.07831600
H	2.65285300	-4.68919300	5.07831600

H	5.38738700	0.04715800	-5.07831600
H	-5.38738700	0.04715800	5.07831600
H	2.48218600	-0.03500600	5.05425000
H	-2.48218600	-0.03500600	-5.05425000
H	-1.21077700	2.16713900	5.05425000
H	1.21077700	2.16713900	-5.05425000
H	-2.73453400	4.64203500	-5.07831600
H	2.73453400	4.64203500	5.07831600
H	-2.56639700	2.37883700	-5.02634200
H	2.56639700	2.37883700	5.02634200
H	3.34333200	1.03314700	-5.02634200
H	-3.34333200	1.03314700	5.02634200
H	1.27140900	-2.13213300	-5.05425000
H	-1.27140900	-2.13213300	5.05425000
H	0.77693500	-3.41198300	5.02634200

1b, Charge = 0, singlet, B3LYP/6-31G(d), E = –5956.3530471 Hartree, ZPVE = 1.857237 Hartree / Particle.

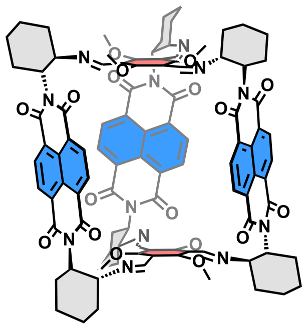

C	-0.27193100	-3.58905800	8.16619600
C	-0.28317700	-2.33868000	7.26848900
C	-1.69872700	-1.94325000	6.78384600
C	-2.63717400	-1.81652400	8.00425900
C	-2.63972200	-3.07231100	8.88490000
C	-1.22036400	-3.40996000	9.36029200
N	0.68896800	-2.45352800	6.14238600
C	1.72802200	-1.49840600	6.09558800
C	2.69399700	-1.60081600	4.96870300
C	2.63941200	-2.70743400	4.09102200
C	1.60957000	-3.66825700	4.19842600
C	0.53856400	-3.49869100	5.21701700
C	3.68696100	-0.64587500	4.81470900
C	4.64372900	-0.76966200	3.78902200
C	4.63440900	-1.87194300	2.94816600
C	3.63968600	-2.86392600	3.09576600
C	3.63498600	-4.01480700	2.27518100
C	4.75137800	-4.25067900	1.32045700
N	5.70130600	-3.21647700	1.17164900
C	5.66949800	-2.00963900	1.88846500
C	1.59617500	-4.76692200	3.35290600
C	2.62226500	-4.95221700	2.40656100
C	6.81218000	-3.45441700	0.20432800
C	6.72892700	-2.51997900	-1.02649300
C	7.89075900	-2.84491200	-1.99123000
C	9.26201400	-2.80356000	-1.30530800
C	9.30397400	-3.76251800	-0.10813800
C	8.18806100	-3.43481800	0.89426700
N	5.47619200	-2.77961400	-1.72361800

C	4.72322800	-1.79193600	-2.01313500
C	3.45743700	-1.92931100	-2.76614500
C	2.60642300	-3.04927000	-2.66449200
C	1.40428700	-3.13309800	-3.39696100
C	1.04932600	-2.05104700	-4.22884400
C	1.87473600	-0.91475600	-4.35617400
C	3.06933200	-0.86675300	-3.60817700
C	0.56894200	-4.34167400	-3.22471700
N	-0.14764100	-4.83593200	-4.15665200
C	-0.88730100	-6.06271300	-3.89084400
C	-2.36213100	-5.85080500	-4.30917200
C	-3.19529200	-7.14495300	-4.28739900
C	-2.53916000	-8.22897400	-5.15449500
C	-1.09314200	-8.48408500	-4.70893000
C	-0.27184100	-7.18935700	-4.74993200
N	-3.01721000	-4.75436600	-3.53777200
C	-3.37218400	-3.59167400	-4.25625800
C	-4.01548000	-2.49029900	-3.49035500
C	-4.38311400	-2.69080500	-2.14033700
C	-4.05023400	-3.88768000	-1.46804500
C	-3.26405100	-4.93901700	-2.16800300
C	-5.10808700	-1.68202800	-1.45305900
C	-5.55112500	-1.91727500	-0.13158900
C	-5.22475800	-3.10471900	0.50485900
C	-4.45401600	-4.08053300	-0.15582700
C	-5.38788200	-0.46069700	-2.10525300
C	-6.11977200	0.61565500	-1.38474000
N	-6.64203900	0.30410800	-0.11924700
C	-6.42178500	-0.92065200	0.54823400
C	-4.31198200	-1.28909600	-4.11549600
C	-4.97793900	-0.26500500	-3.41513900
C	-7.50681300	1.30393600	0.57276100
C	-6.71612100	2.55962600	1.01216700
C	-7.66924000	3.53070300	1.74341500
C	-8.91681300	3.87224800	0.91913200
C	-9.67965700	2.59945900	0.52841600
C	-8.77037000	1.63207300	-0.24283500
N	-5.67911300	2.15140000	1.95062600
C	-4.48196700	2.53884500	1.74323600
C	-3.35958600	2.23224300	2.65667900
C	-3.25748000	1.03369500	3.39283100
C	-2.16386800	0.78154500	4.24670800
C	-1.14557400	1.75307800	4.33651700
C	-1.21300500	2.96337700	3.61589300
C	-2.32202700	3.18029800	2.77216500
O	-4.19955000	0.05135300	3.23981000
C	-5.26455300	0.04709300	4.20458200
O	-2.38022800	4.30261900	1.98927500
C	-3.12264400	5.40766900	2.53030700
C	-0.09731800	3.92320900	3.76451800
N	-0.24903500	5.18789700	3.70263400
C	0.91233500	6.04050000	3.91949800
C	1.00424500	7.05563900	2.75499300
C	2.05697700	8.15496400	2.98453400
C	1.80476500	8.88059300	4.31393800
C	1.76794700	7.88533900	5.48128300
C	0.70487200	6.80513500	5.24556000
O	-0.02568000	1.50695800	5.08563100
C	-0.04122000	2.02365400	6.42645800
C	-2.13448500	-0.50502000	4.97609900

N	-1.62138200	-0.64070500	6.13566600
O	-6.94807000	-1.16751500	1.62328400
O	-6.27839700	1.71965300	-1.88958000
O	-3.18966200	-3.50319100	-5.46145900
O	-2.87102000	-5.93501800	-1.57441600
O	2.90433500	-4.06114900	-1.79084600
C	3.60476900	-5.18554200	-2.34777400
O	3.85281600	0.25630000	-3.63666700
C	4.94559400	0.23591500	-4.56964900
C	1.42160100	0.17657600	-5.24599900
N	2.22098000	0.91695500	-5.90865700
C	1.66088600	1.92581600	-6.79817200
C	2.33569700	3.28432600	-6.49139900
C	1.99255600	4.38179200	-7.51476200
C	2.33044300	3.92140100	-8.94001200
C	1.61971600	2.60236900	-9.27107700
C	1.98262200	1.51495600	-8.25207400
O	-0.15169300	-2.05608000	-4.88720300
C	-0.12194500	-2.52878900	-6.24392500
N	2.09073200	3.73420500	-5.08998300
C	3.20773700	3.77243300	-4.22689500
C	2.96785700	4.20937400	-2.82510900
C	1.69945700	4.70645900	-2.44831300
C	0.61680900	4.69400500	-3.35551300
C	0.79192000	4.11167800	-4.71329200
C	1.51264400	5.23773400	-1.14507900
C	2.57732700	5.19457000	-0.21778100
C	3.80069200	4.66021800	-0.59194000
C	4.00295300	4.18881000	-1.90328600
C	0.26976300	5.81382200	-0.79683100
C	-0.77543900	5.80309700	-1.70734800
C	-0.60864100	5.22190400	-2.97902200
C	2.38384200	5.72001400	1.16055300
N	1.17824300	6.38605700	1.43296800
C	0.10683100	6.48897700	0.51887500
O	4.33016200	3.49034900	-4.61962900
O	-0.15752500	3.99085500	-5.47670200
O	-0.89925600	7.13123300	0.78145900
O	3.25395700	5.59524400	2.01270900
O	6.47428900	-1.11319700	1.67043300
O	4.85636500	-5.30809300	0.71671400
O	1.85045900	-0.64278200	6.95963000
O	-0.42130500	-4.25753600	5.25755500
H	0.53237200	2.76371900	-9.26929000
H	-3.04592100	-3.92177400	8.31765200
H	9.47337800	-1.78122300	-0.96106000
H	2.75562300	7.41543100	5.59099500
H	-1.09273400	-8.89089000	-3.68772300
H	-8.62271900	4.41473800	0.00942400
H	3.41702400	3.78338200	-9.03858400
H	-0.85273000	-2.60194800	10.00943400
H	9.19054700	-4.79608500	-0.46632600
H	0.84822500	9.42119800	4.26703300
H	-2.54468600	-7.91595100	-6.20873000
H	-10.05837900	2.10940400	1.43717300
H	2.04652700	4.70250100	-9.65582100
H	-1.22421600	-4.32364200	9.96715000
H	10.27733900	-3.70907200	0.39466600
H	2.58464000	9.63490700	4.47491600
H	-3.12865500	-9.15220900	-5.09757300

H	-10.55563300	2.84751500	-0.08333800
H	2.55814400	5.28721500	-7.26100100
H	0.75411300	-3.76117500	8.51503100
H	8.18098700	-4.16682000	1.71170200
H	2.01264800	8.86328300	2.14763700
H	-4.20394000	-6.91897900	-4.65569600
H	-9.30195300	0.69647700	-0.45767500
H	0.92987500	4.63162100	-7.45407200
H	-0.57336300	-4.46736700	7.58904900
H	8.37151900	-2.45183300	1.33638800
H	3.05930700	7.71830200	2.99135600
H	-3.29516600	-7.50902800	-3.26130900
H	-8.49217400	2.07830600	-1.20141200
H	3.41189800	3.10916400	-6.52080600
H	0.09290900	-1.49441200	7.84773600
H	0.02382500	7.52732500	2.67724900
H	6.62332800	-4.46481500	-0.16061800
H	-2.34250200	-5.46999400	-5.33110700
H	-7.80945800	0.79273200	1.48754600
H	-1.42847500	5.19234500	-3.68855700
H	0.79004500	-5.48648600	3.44684600
H	5.41101800	-0.01563500	3.65062600
H	4.60065800	4.62826400	0.13982300
H	-4.17829100	-5.00302500	0.34360000
H	-5.19495400	0.68453600	-3.89233700
H	4.96956800	3.80745200	-2.21323500
H	3.71409400	0.19332800	5.50089400
H	-4.02522800	-1.15655600	-5.15282600
H	-5.57778200	-3.26779100	1.51709900
H	-1.71962600	6.25388900	-1.42248900
H	2.63897500	-5.83032200	1.77055700
H	-3.30584000	-2.91986800	9.74321800
H	1.88612800	2.26577200	-10.28072500
H	10.04501800	-3.05971400	-2.02984300
H	1.56133000	8.40704800	6.42404000
H	-0.62208900	-9.24042300	-5.34902500
H	-9.56454500	4.54718600	1.49233700
H	-3.64803800	-1.59250000	7.64175700
H	1.45189700	0.58068300	-8.47322200
H	-7.10937600	4.43912200	1.99791400
H	0.75392900	-7.36723400	-4.40410500
H	-2.31613100	-0.94732300	8.59456700
H	3.05578500	1.28810000	-8.31453200
H	-7.96561800	3.06832900	2.69494700
H	-0.19551500	-6.82836800	-5.78477600
H	7.71529800	-3.84328500	-2.41491200
H	-0.29381700	7.26254600	5.22470700
H	7.85261500	-2.13816400	-2.82937900
H	0.69897200	6.07809400	6.06703400
H	1.84609500	5.46300800	3.97953300
H	6.81378800	-1.47318400	-0.70087600
H	-2.08789200	-2.72004000	6.10993400
H	0.57145800	2.02138100	-6.68406000
H	-6.30058100	3.06199400	0.12675100
H	-0.84286600	-6.35315900	-2.83128100
H	-4.21615100	3.16736100	0.88181400
H	0.64135400	-4.81436000	-2.23524200
H	-2.62898800	-1.33906100	4.45881600
H	0.33067400	0.28017000	-5.32896500
H	4.99396800	-0.75935400	-1.75196100

H	0.87914300	3.46524400	3.97553700
H	5.41261600	1.21782800	-4.49318800
H	5.66380000	-0.55173800	-4.30710300
H	4.56834300	0.09338400	-5.58475500
H	-1.15870700	-2.51537900	-6.58016200
H	0.49275300	-1.86907000	-6.87007600
H	0.25465000	-3.55362500	-6.27494000
H	3.80277400	-5.85103100	-1.50751800
H	2.98684900	-5.69200300	-3.10064800
H	4.55273500	-4.85871000	-2.78091900
H	0.89092800	1.68637300	6.87972800
H	-0.08856000	3.12039100	6.41790400
H	-0.88444100	1.60388300	6.97949100
H	-5.93607900	-0.75336700	3.89405600
H	-4.87367300	-0.14933000	5.21144700
H	-5.79947600	0.99898300	4.17565400
H	-3.01153300	6.21557600	1.80708500
H	-4.18116700	5.14175000	2.64847500
H	-2.69181100	5.71608500	3.48547000

1c, Charge = 0, singlet, B3LYP/6-31G(d), E = –7894.17461608 Hartree, ZPVE = 1.832249 Hartree / Particle.

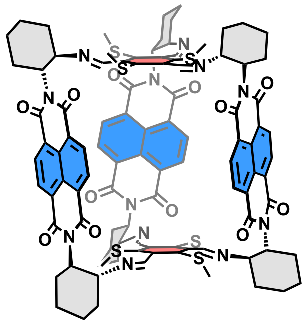

C	-5.07233000	7.34175700	-1.15912400
C	-4.70648000	5.85016800	-1.29802600
C	-4.97487500	5.02070500	-0.01501400
C	-6.40933600	5.28917400	0.49184800
C	-6.72586900	6.77931500	0.65786600
C	-6.51274700	7.52413300	-0.66482000
N	-3.30661700	5.68331200	-1.79149000
C	-2.24092100	6.12332000	-0.98878600
C	-0.86301700	5.93837000	-1.51770100
C	-0.66161300	5.27110300	-2.74621800
C	-1.75852800	4.85624500	-3.53462800
C	-3.14693700	5.15311600	-3.09075100
C	-1.53675700	4.18425600	-4.72664900
C	-0.22612000	3.87768900	-5.13981900
C	0.86301700	4.28355600	-4.38392700
C	0.66161300	5.01384800	-3.19179800
C	0.22612000	6.39005900	-0.78826500
C	1.53675700	6.18552600	-1.26034400
C	1.75852800	5.48920200	-2.43831600
C	3.14693700	5.25322900	-2.91735100
N	3.30661700	4.39313400	-4.02614600
C	2.24092100	3.91797600	-4.80855500
C	4.70648000	4.04921100	-4.41737900
C	4.97487500	2.52335700	-4.34054900
C	6.40933600	2.21863700	-4.82648200
C	6.72586900	2.81993300	-6.19999100

C	6.51274700	4.33782100	-6.18367900
C	5.07233000	4.67471300	-5.77858400
N	4.89367700	2.07040900	-2.95078700
C	3.97738900	1.24726200	-2.62990700
C	3.91217400	0.63662900	-1.26360100
C	3.90346000	1.40529400	-0.08082900
C	3.91217400	0.77599600	1.18313700
C	3.90346000	-0.63264700	1.25743500
C	3.91217400	-1.41262500	0.08046400
C	3.90346000	-0.77264700	-1.17660600
C	3.97738900	-2.90119700	0.23479200
N	4.89367700	-3.59066200	-0.31763300
C	4.97487500	-5.02070500	-0.01501600
C	4.70648000	-5.85016700	-1.29802900
C	5.07233000	-7.34175600	-1.15912800
C	6.51274700	-7.52413300	-0.66482400
C	6.72586900	-6.77931600	0.65786200
C	6.40933600	-5.28917400	0.49184600
C	3.97738900	1.65393500	2.39511500
N	4.89367700	1.52025200	3.26842000
C	4.97487500	2.49734800	4.35556700
C	4.70648000	1.80095700	5.71540900
C	5.07233000	2.66704400	6.93771200
C	6.51274700	3.18631300	6.84850200
C	6.72586900	3.95938400	5.54212800
C	6.40933600	3.07053800	4.33463600
N	3.30661700	1.29017800	5.81763900
C	2.24092100	2.20534300	5.79734500
C	0.86301700	1.65481400	5.90163200
C	0.66161300	0.25725400	5.93801800
C	1.75852800	-0.63295700	5.97294600
C	3.14693700	-0.10011400	6.00810500
C	1.53675700	-2.00127300	5.98699600
C	0.22612000	-2.51237200	5.92808500
C	-0.86301700	-1.65481700	5.90163100
C	-0.66161300	-0.25725800	5.93801800
C	-0.22612000	2.51236800	5.92808700
C	-1.53675700	2.00127000	5.98699700
C	-1.75852800	0.63295400	5.97294600
C	-3.14693700	0.10011100	6.00810500
N	-3.30661700	-1.29018100	5.81763900
C	-2.24092100	-2.20534600	5.79734400
C	-4.70648000	-1.80096000	5.71540800
C	-4.97487500	-2.49735000	4.35556500
C	-6.40933600	-3.07054000	4.33463400
C	-6.72586900	-3.95938700	5.54212600
C	-6.51274700	-3.18631600	6.84850000
C	-5.07233000	-2.66704800	6.93771000
N	3.30661700	-5.68331100	-1.79149300
C	2.24092100	-6.12332000	-0.98879000
C	0.86301700	-5.93837000	-1.51770400
C	0.66161300	-5.27110100	-2.74622000
C	1.75852800	-4.85624300	-3.53463000
C	3.14693700	-5.15311400	-3.09075300
C	-0.66161300	-5.01384600	-3.19180000
C	-1.75852800	-5.48920000	-2.43831800
C	-1.53675700	-6.18552600	-1.26034800
C	-0.22612000	-6.39005900	-0.78826900
C	-3.14693700	-5.25322700	-2.91735300
N	-3.30661700	-4.39313200	-4.02614800

C	-2.24092100	-3.91797400	-4.80855700
C	-0.86301700	-4.28355400	-4.38392900
C	1.53675700	-4.18425400	-4.72665100
C	0.22612000	-3.87768700	-5.13982100
C	-4.70648000	-4.04920900	-4.41738100
C	-4.97487500	-2.52335500	-4.34055100
C	-6.40933600	-2.21863400	-4.82648300
C	-6.72586900	-2.81993000	-6.19999300
C	-6.51274700	-4.33781800	-6.18368100
C	-5.07233000	-4.67471000	-5.77858600
N	-4.89367700	-2.07040700	-2.95078800
C	-3.97738900	-1.24726100	-2.62990700
C	-3.91217400	-0.63662800	-1.26360100
C	-3.90346000	0.77264700	-1.17660600
C	-3.91217400	1.41262500	0.08046400
C	-3.90346000	0.63264600	1.25743500
C	-3.91217400	-0.77599700	1.18313700
C	-3.90346000	-1.40529400	-0.08082900
C	-3.97738900	-1.65393600	2.39511400
N	-4.89367700	-1.52025400	3.26842000
C	-3.97738900	2.90119700	0.23479400
N	-4.89367700	3.59066200	-0.31763100
O	-2.43105800	-3.22101800	-5.79741000
O	2.43105800	-6.63121300	0.10921800
O	-4.10808700	-5.77608000	-2.37254700
O	4.10808700	-4.94272400	-3.81596000
O	2.43105800	3.22102100	-5.79740800
O	-2.43105800	-3.41019600	5.68818800
O	4.10808700	5.77608100	-2.37254400
O	-4.10808700	0.83335200	6.18850500
O	-2.43105800	6.63121300	0.10922200
O	2.43105800	3.41019300	5.68819000
O	-4.10808700	4.94272600	-3.81595800
O	4.10808700	-0.83335500	6.18850500
H	6.07948800	2.36790700	-6.96559900
H	-6.07948800	-4.84843600	5.53346500
H	6.07948800	4.84843300	5.53346700
H	-6.07948800	7.21633800	1.43213600
H	6.07948800	-7.21633900	1.43213200
H	-6.07948800	-2.36790400	-6.96560100
H	7.21898700	4.79794400	-5.47723200
H	-7.21898700	-2.34445400	6.89375500
H	7.21898700	2.34445100	6.89375700
H	-7.21898700	7.14239400	-1.41652100
H	7.21898700	-7.14239300	-1.41652500
H	-7.21898700	-4.79794100	-5.47723400
H	6.72679600	4.77056700	-7.16859800
H	-6.72679600	-3.82290800	7.71572900
H	6.72679600	3.82290400	7.71573100
H	-6.72679600	8.59347100	-0.54712900
H	6.72679600	-8.59347100	-0.54713300
H	-6.72679600	-4.77056300	-7.16860000
H	4.93719700	5.76089500	-5.70364000
H	-4.93719700	-2.05905500	7.84090100
H	4.93719700	2.05905100	7.84090200
H	-4.93719700	7.81994600	-2.13725700
H	4.93719700	-7.81994500	-2.13726100
H	-4.93719700	-5.76089200	-5.70364300
H	4.38558100	4.31020600	-6.54749100
H	-4.38558100	-3.51519500	7.00649100

H	4.38558100	3.51519100	7.00649300
H	-4.38558100	7.82539700	-0.45899800
H	4.38558100	-7.82539700	-0.45900200
H	-4.38558100	-4.31020300	-6.54749300
H	5.31786100	4.52081900	-3.64688300
H	-5.31786100	-0.89788700	5.73858500
H	-5.31786100	5.41870300	-2.09170000
H	5.31786100	0.89788400	5.73858500
H	5.31786100	-5.41870200	-2.09170200
H	-5.31786100	-4.52081700	-3.64688500
H	-0.04781300	3.31700800	-6.05095900
H	0.04781300	-3.58178400	5.89809000
H	-0.04781300	3.58178000	5.89809200
H	0.04781300	6.89878800	0.15287100
H	-0.04781300	-6.89878800	0.15286700
H	0.04781300	-3.31700400	-6.05096100
H	2.39076800	6.55174000	-0.70164300
H	-2.39076800	2.66822600	6.02479700
H	2.39076800	-3.88351200	-5.32315200
H	-2.39076800	-6.55174000	-0.70164700
H	-2.39076800	3.88351400	-5.32315000
H	2.39076800	-2.66822900	6.02479500
H	-7.75936500	-4.32078900	5.47206800
H	7.75936500	2.57856000	-6.47794500
H	7.75936500	4.32078600	5.47207000
H	-7.75936500	6.89934400	1.00587900
H	7.75936500	-6.89934500	1.00587500
H	-7.75936500	-2.57855700	-6.47794700
H	-6.54393300	-3.62348100	3.39689600
H	6.54393300	1.13006100	-4.83647500
H	-6.54393300	-1.13005800	-4.83647500
H	6.54393300	-4.75354000	1.43957600
H	-7.11127800	-2.22633400	4.30234200
H	7.11127800	2.61277300	-4.07923200
H	-7.11127800	-2.61277100	-4.07923400
H	7.11127800	-4.83910500	-0.22311100
H	7.11127800	2.22633200	4.30234400
H	-7.11127800	4.83910500	-0.22310900
H	6.54393300	3.62347900	3.39689800
H	-6.54393300	4.75353900	1.43957800
H	-4.25629600	5.31444700	0.76095000
H	4.25629600	3.31622400	4.22197200
H	-4.25629600	-3.31622600	4.22197000
H	4.25629600	1.99822300	-4.98291900
S	3.80528600	-1.47063000	2.85040600
S	-3.80528600	1.73321100	-2.69880500
S	3.80528600	-1.73320900	-2.69880600
S	-3.80528600	-3.20383900	-0.15160200
H	-4.25629600	-1.99822100	-4.98292000
H	4.25629600	-5.31444700	0.76094800
H	-3.23712800	-0.88570200	-3.35393300
H	3.23712800	-3.34744300	0.90992400
H	-3.23712800	-2.46174000	2.44400700
H	3.23712800	0.88570400	-3.35393300
S	-3.80528600	1.47062800	2.85040600
S	3.80528600	3.20383900	-0.15160000
H	3.23712800	2.46173900	2.44400900
H	-3.23712800	3.34744300	0.90992600
C	5.57841200	3.66934800	-0.14735300
H	5.60039600	4.75592800	-0.26167600

H	6.05587400	3.37919100	0.79230000
H	6.06695900	3.20066600	-1.00271900
C	5.57841200	-1.96228500	-3.10407200
H	5.60039600	-2.60458200	-3.98791700
H	6.05587400	-1.00344300	-3.32261500
H	6.06695900	-2.46871200	-2.27049900
C	5.57841200	-1.70706300	3.25142500
H	5.60039600	-2.15134600	4.24959300
H	6.05587400	-2.37574700	2.53031500
H	6.06695900	-0.73195300	3.27321700
C	-5.57841200	1.70706100	3.25142600
H	-5.60039600	2.15134400	4.24959400
H	-6.05587400	2.37574600	2.53031700
H	-6.06695900	0.73195100	3.27321700
C	-5.57841200	-3.66934800	-0.14735500
H	-5.60039600	-4.75592800	-0.26167800
H	-6.05587400	-3.37919200	0.79229800
H	-6.06695900	-3.20066500	-1.00272100
C	-5.57841200	1.96228700	-3.10407100
H	-5.60039600	2.60458400	-3.98791500
H	-6.05587400	1.00344500	-3.32261500
H	-6.06695900	2.46871300	-2.27049700

4.2. Geometries of bridges

The crowded substitution in trialdehydes **3c** forces the formyl and (thio)ether groups to rotate out of the plane of the benzene ring, while the geometry in 4-(methylthio)benzaldehyde shows clearly that reducing the steric bulk prefers the thioether groups to be in plane with the benzene ring (Figure S74). This is unlike in the trialdehyde **3b**. Consequently, the lower reactivity of trialdehyde **3c** compared to **3b** can be attributed to differences in steric repulsion between the EtS (or MeO) substituents and the formyl groups in the two aldehydes. For a nucleophilic attack on **3c** to occur, the formyl groups must rotate to permit the access of the nucleophile, which necessarily increases the reaction barrier for the imine formation.

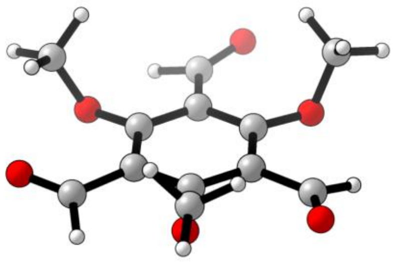

3b

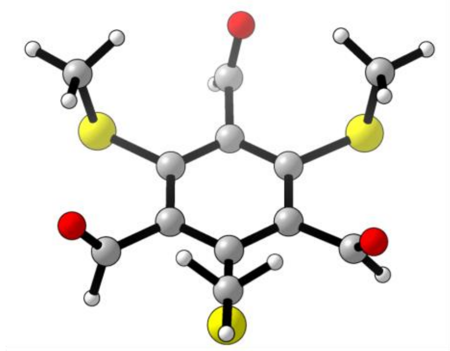

3c

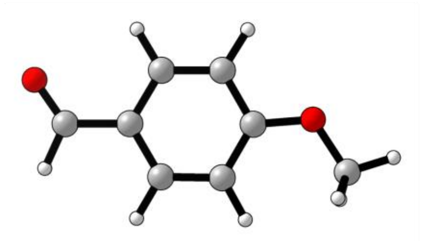

4-methoxybenzaldehyde

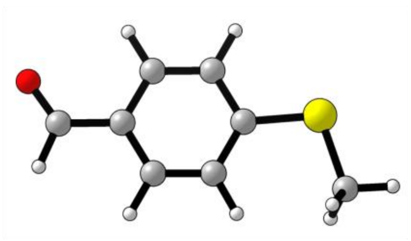

4-(methylthio)benzaldehyde

Fig. S74 Comparison of the geometries of **3b**, **3c**, 4-methoxybenzaldehyde, and 4-(methylthio)benzaldehyde. Structures are obtained at B3LYP/6-31G(d) level of theory.

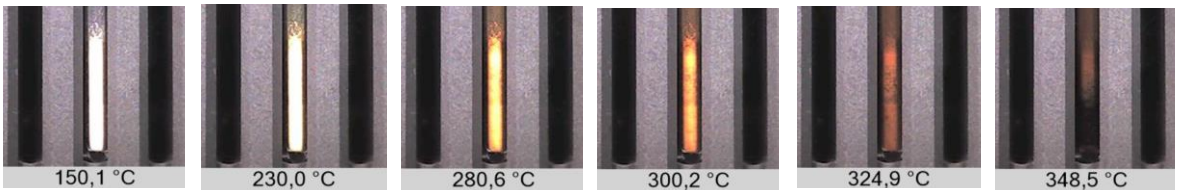

Fig. S75 Snapshots of the sample of crystalline **2b** at different temperatures during melting. The sample starts decomposing <300 °C, while a melting process appears to occur at ~340 °C when the majority of the sample underwent decomposition.

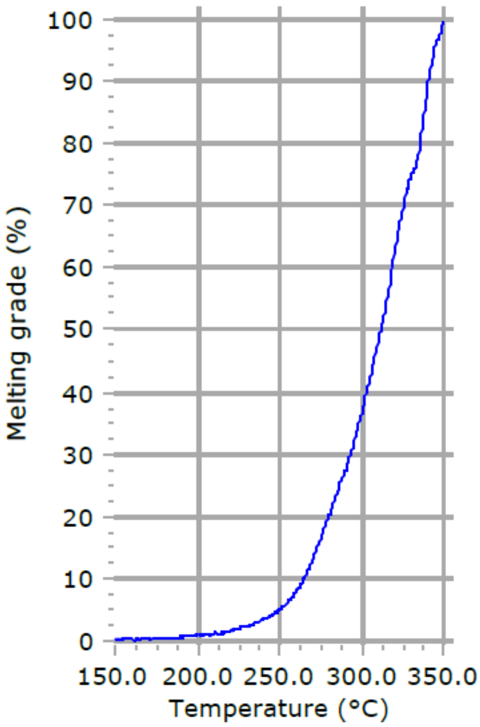

Fig. S76 The melting profile of crystalline sample of cage **2b** demonstrating decomposition of the sample before reaching the melting point. Similar results were obtained also for cages **1**.

5. References

(1) Larrow, J. F.; Jacobsen, E. N. (R,R)-N,N'-Bis(3,5-Di-Tert-Butylsalicylidene)-1,2-Cyclohexyldiamine Manganese(III) Chloride, a Highly Enantioselective Epoxidation Catalyst. *Org. Synth.* **1998**, *75*, 1. https://doi.org/10.15227/orgsyn.075.0001.

(2) Muth, A.; Madan, M.; Archer, J. J.; Ocampo, N.; Rodriguez, L.; Phanstiel, O. I. Polyamine Transport Inhibitors: Design, Synthesis, and Combination Therapies with Difluoromethylornithine. *J. Med. Chem.* **2014**, *57* (2), 348–363. https://doi.org/10.1021/jm401174a.

(3) Lauer, J. C.; Zhang, W.-S.; Rominger, F.; Schröder, R. R.; Mastalerz, M. Shape-Persistent [4+4] Imine Cages with a Truncated Tetrahedral Geometry. *Chem. Eur. J.* **2018**, *24* (8), 1816–1820. https://doi.org/10.1002/chem.201705713.

(4) Halder, A.; Karak, S.; Addicoat, M.; Bera, S.; Chakraborty, A.; Kunjattu, S. H.; Pachfule, P.; Heine, T.; Banerjee, R. Ultrastable Imine-Based Covalent Organic Frameworks for Sulfuric Acid Recovery: An Effect of Interlayer Hydrogen Bonding. *Angew. Chem. Int. Ed.* **2018**, *57* (20), 5797–5802. https://doi.org/10.1002/anie.201802220.

(5) Nalluri, S. K. M.; Liu, Z.; Wu, Y.; Hermann, K. R.; Samanta, A.; Kim, D. J.; Krzyaniak, M. D.; Wasielewski, M. R.; Stoddart, J. F. Chiral Redox-Active Isosceles Triangles. *J. Am. Chem. Soc.* **2016**, *138* (18), 5968–5977. https://doi.org/10.1021/jacs.6b02086.

(6) Šolomek, T.; Powers-Riggs, N. E.; Wu, Y.-L.; Young, R. M.; Krzyaniak, M. D.; Horwitz, N. E.; Wasielewski, M. R. Electron Hopping and Charge Separation within a Naphthalene-1,4:5,8-Bis(Dicarboximide) Chiral Covalent Organic Cage. *J. Am. Chem. Soc.* **2017**, *139* (9), 3348–3351. https://doi.org/10.1021/jacs.7b00233.

(7) Huang, H.-H.; Song, K. S.; Prescimone, A.; Aster, A.; Cohen, G.; Mannancherry, R.; Vauthey, E.; Coskun, A.; Šolomek, T. Porous Shape-Persistent Rylene Imine Cages with Tunable Optoelectronic Properties and Delayed Fluorescence. *Chem. Sci.* **2021**, *12* (14), 5275–5285. https://doi.org/10.1039/D1SC00347J.

(8) Percec, V.; Wilson, D. A.; Leowanawat, P.; Wilson, C. J.; Hughes, A. D.; Kaucher, M. S.; Hammer, D. A.; Levine, D. H.; Kim, A. J.; Bates, F. S.; Davis, K. P.; Lodge, T. P.; Klein, M. L.; DeVane, R. H.; Aqad, E.; Rosen, B. M.; Argintaru, A. O.; Sienkowska, M. J.; Rissanen, K.; Nummelin, S.; Ropponen, J. Self-Assembly of Janus Dendrimers into Uniform Dendrimersomes and Other Complex Architectures. *Science* **2010**, *328* (5981), 1009–1014. https://doi.org/10.1126/science.1185547.

(9) Hong, S.; Rohman, Md. R.; Jia, J.; Kim, Y.; Moon, D.; Kim, Y.; Ko, Y. H.; Lee, E.; Kim, K. Porphyrin Boxes: Rationally Designed Porous Organic Cages. *Angew. Chem. Int. Ed.* **2015**, *54* (45), 13241–13244. https://doi.org/10.1002/anie.201505531.

(10) Sheldrick, G. M. It SHELXT – Integrated Space-Group and Crystal-Structure Determination. *Acta Crystallogr. A* **2015**, *71* (1), 3–8. https://doi.org/10.1107/S2053273314026370.

(11) Dolomanov, O. V.; Bourhis, L. J.; Gildea, R. J.; Howard, J. A. K.; Puschmann, H. It OLEX2: A Complete Structure Solution, Refinement and Analysis Program. *J. Appl. Cryst.* **2009**, *42* (2), 339–341. https://doi.org/10.1107/S0021889808042726.

(12) Sheldrick, G. M. Crystal Structure Refinement with It SHELXL. *Acta Crystallogr. C* **2015**, *71* (1), 3–8. https://doi.org/10.1107/S2053229614024218.

(13) Frisch, M. J.; Trucks, G. W.; Schlegel, H. B.; Scuseria, G. E.; Robb, M. A.; Cheeseman, J. R.; Scalmani, G.; Barone, V.; Petersson, G. A.; Nakatsuji, H.; Li, X.; Caricato, M.; Marenich, A. V.; Bloino, J.; Janesko, B. G.; Gomperts, R.; Mennucci, B.; Hratchian, H. P.; Ortiz, J. V.; Izmaylov, A. F.; Sonnenberg, J. L.; Williams-Young, D.; Ding, F.; Lipparini, F.; Egidi, F.; Goings, J.; Peng, B.; Petrone, A.; Henderson, T.; Ranasinghe, D.; Zakrzewski, V. G.; Gao, J.; Rega, N.; Zheng, G.; Liang, W.; Hada, M.; Ehara, M.; Toyota, K.; Fukuda, R.; Hasegawa, J.; Ishida, M.; Nakajima, T.; Honda, Y.; Kitao, O.; Nakai, H.; Vreven, T.; Throssell, K.; Montgomery, J. A., Jr.; Peralta, J. E.; Ogliaro, F.; Bearpark, M. J.; Heyd, J. J.; Brothers, E. N.; Kudin, K. N.; Staroverov, V. N.; Keith, T. A.; Kobayashi, R.; Normand, J.; Raghavachari, K.; Rendell, A. P.; Burant, J. C.; Iyengar, S. S.; Tomasi, J.; Cossi, M.; Millam, J. M.; Klene, M.; Adamo, C.; Cammi, R.; Ochterski, J. W.; Martin, R. L.; Morokuma, K.; Farkas, O.; Foresman, J. B.; Fox, D. J. Gaussian˜16 Revision C.02, 2016.
